# Pnictogen-Bonding Catalysis: Copolymerization of CO_2_ and Epoxides on Antimony(V) Platforms

**DOI:** 10.1021/acscatal.5c03781

**Published:** 2025-10-15

**Authors:** Chenggang Jiang, Eryn Lee, Jonathan Schaefer, Matthew W. Holtcamp, Tzu-Pin Lin, François P. Gabbaï

**Affiliations:** † Department of Chemistry, Texas A&M University, College Station, Texas 77843, United States; ‡ ExxonMobil Technology and Engineering Company, Baytown, Texas 77520, United States

**Keywords:** antimony, pnictogen bonding, polymer, epoxide, carbon dioxide

## Abstract

The copolymerization of CO_2_ and epoxides to access polycarbonates represents a promising strategy for CO_2_ utilization and for the production of useful polymers. Aiming to explore alternative transition-metal-free approaches that support this chemistry, we have investigated a series of triaryl-catecholatostiboranes as pnictogen-bonding platforms for the copolymerization of CO_2_ and cyclohexene oxide (CHO). Our survey of these antimony species has identified motifs that promote this polymerization reaction efficiently, provided that bis­(triphenylphosphine)­iminium chloride is administered as an activator. By coupling these polymerization studies with a careful assessment of the structure, electronic attributes and Lewis acidity of the catecholatostiboranes, this work shows that high activity is generally observed with the weakest pnictogen-bond donors or Lewis acids investigated. Mechanistic studies, which indicate that the polymerization reaction is first order in stiborane, reveal a nonlinear dependence on the CO_2_ pressure. This nonlinear dependence could be satisfactorily modeled based on a pre-equilibrium process involving the reversible insertion of the gaseous monomer into the growing chain. Altogether these findings greatly expand the reach of pnictogen bond catalysis while also providing an entry for the use of heavy group 15 elements as competent platforms for CO_2_ utilization.

## Graphical abstract

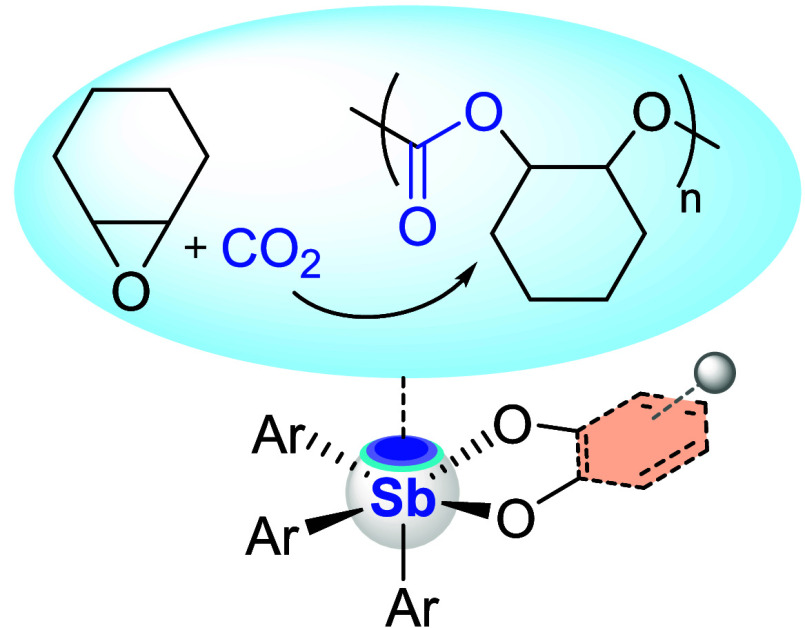

## Introduction

Pnictogen-bonding catalysis has recently emerged as a counterpart of Lewis acid catalysis, allowing for the mild activation of organic substrates in various reactions.^1–4^ Because pnictogen bonds are typically weaker than classical dative bonds, the use of pnictogen bond donors as activators may prevent the formation of strong adducts whose stability could limit or even prevent reaction turnover. To date much progress has been made with antimony derivatives^5–13^ which have been used both in the trivalent^14^ and pentavalent state^15–27^ as catalysts. The prevalence of antimony in this chemistry may be correlated to the unique electronic features and size of this element which promote interaction with electron-rich substrates.^28–30^ The association of antimony derivatives with electron-rich substrates is often correlated to the presence of electron-poor regions that exist on the electrostatic potential surface of the compounds above the group 15 element, trans from a polarized σ-bond.^31^ These regions are typically referred to as σ-holes (Figure 1). The depth of these holes can be readily adjusted using various strategies, leading to an increase in the stability of the resulting pnictogen bond.^32^ Our group introduced a strategy based on the oxidation of triarylstibines into their pentavalent analogs (Figure 1).^18^ Such a strategy enhances the pnictogen bond donor properties of the antimony center, allowing for greater catalytic activity. These favorable effects have been observed in transfer hydrogenation reactions and anion-binding reactions, including Ritter-like reactions.^18^

**Figure 1:**
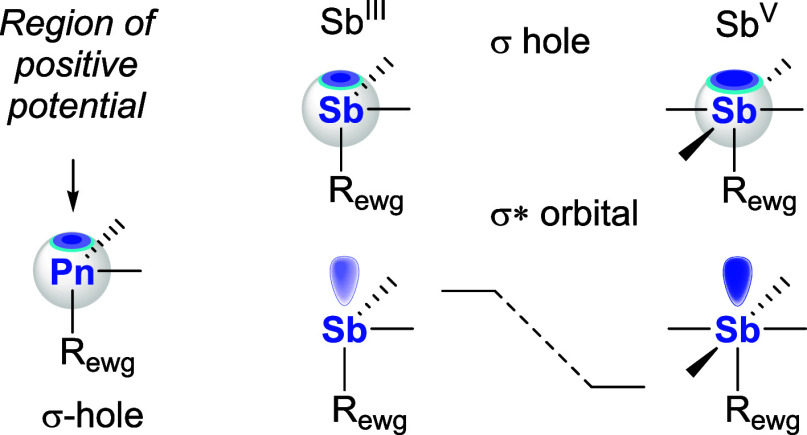
Representations of the σ-hole on a pnictine (left) and oxidation-induced deepening of the σ-hole/stabilization of the σ* orbital in the case of stibines. *R*_ewg_ = electron-withdrawing substituent.

More recently, stiboranes have emerged as unique catalysts for epoxide-opening polyether cyclizations, providing access to selectivities distinct from those observed with conventional Brønsted or Lewis acid catalysts (Scheme 1a).^19–23^ Mechanistic investigations suggest coordination of the epoxide to the antimony center primes the three-member ring for nucleophilic attack by the dangling alcohol. This reactivity is reminiscent of that observed several decades ago with stibonium cations as catalysts for the nucleophilic addition of amines among other nucleophiles^33^ to epoxide (Scheme 1b).^34^ Although not framed in the context of pnictogen bonding catalysis, this early report unambiguously proposed that the stibonium cation engages the epoxide and activates it toward nucleophilic attack by the amine. Another important and often ignored set of reactions promoted by organoantimony­(V) halides includes the cycloaddition of CO_2_ and epoxide to generate cyclic carbonates (Scheme 1c).^35–37^ Analogous reactions of heterocumulenes, such as isocyanates, have also been widely documented.^38–43^

**Scheme 1:**
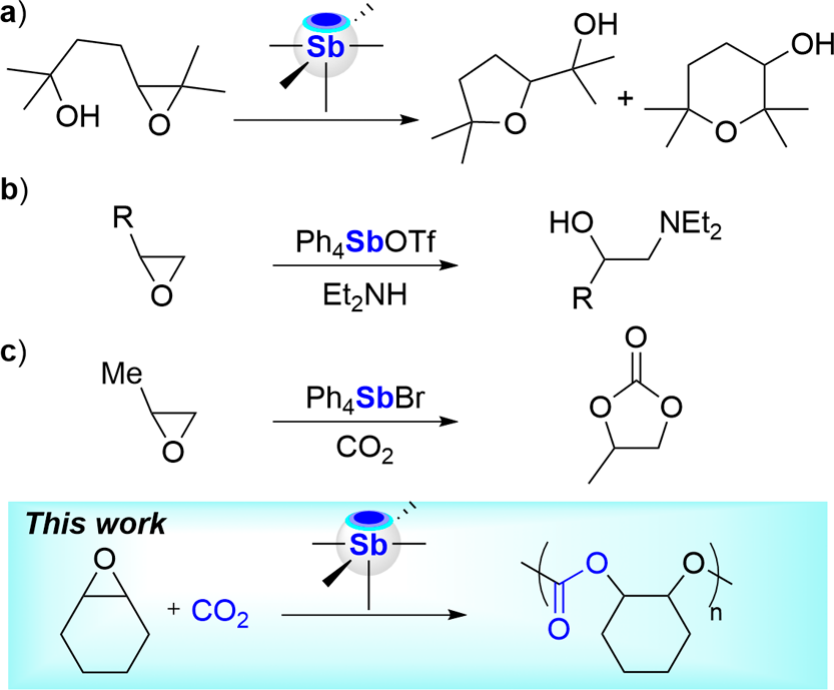
Example of Reactions Involving Epoxides and Antimony­(V) Catalysts^a^

a The objective of the current study is also shown.

Notably absent from the above list of reactions is the copolymerization of CO_2_ and epoxide, a process that continues to attract attention as a possible means to convert the greenhouse gas into valuable polymers.^44,45^ While transition metal catalysts have usually played a dominant role in this area of epoxide chemistry, recent developments have shown that simple organoborane-based systems may also be competent catalysts.^46–48^ Interestingly, these recent efforts found that classical Lewis acids such as B­(C_6_F_5_)_3_ are ill-suited for this chemistry, presumably because of the overwhelming strength with which the boron would engage with epoxide, leading to homopolymerization^48^ or cyclic carbonate formation.^47^ In fact, the success of this approach hinges on the use of much more weakly Lewis acidic boranes such as BEt_3_, suggesting that the interaction of the boron center with the reaction substrate may be akin to a triel bond^49^ rather than a strong polar covalent bond. Inspired by these developments and motivated by modularity and stability considerations, we have now decided to test whether appropriately designed antimony-based pnictogen bond donors could function as catalysts for the copolymerization of CO_2_ and epoxides.^50^ Our results are presented herein.

## Results and Discussion

### The Catalysts

Our initial efforts, which explored the use of simple triaryl stibines such as SbPh_3_ and Sb­(C_6_F_5_)_3_ as possible catalysts, led to no detectable activity in the copolymerization of CO_2_ and cyclohexene oxide. Envisioning that enhanced pnictogen bond donor properties would favorably influence the catalytic activity of these systems, we decided to investigate catecholatostiboranes.^51–53^ To access a set of electronically and sterically differentiated platforms, four triarylstibines and two catecholates were selected as elementary building blocks, leading to catecholatostiboranes **1**–**7** (Scheme 2). This series was expanded upon by the inclusion of **8** and **9**, two additional compounds which were selected to test the effect of increased bulk or increased pnictogen bond donor or, synonymously, Lewis acidic properties. The five stibines involved in these studies were prepared as described in the literature^54–56^ or purchased in the case of SbPh_3_. Their conversion into the final stiboranes also followed established protocols^52,57^ either by combination of *^t^* BuOOH and catechol (route 1, Scheme 2) in the cases of **1**–**4** and **8** or via direct reaction with the corresponding *o*-quinone in the cases of **5**–**7** and **9** (route 2, Scheme 2). The final products are stable to chromatographic purification and isolated in yields ranging from 55 to 75%. While compounds **1** and **9** are known in the literature,^52,57^ the remaining compounds are new and have thus been fully characterized. The ^1^H NMR spectra of all new compounds except **8** exhibit two distinct aromatic resonances corresponding to the aryl substituents, indicating rapid equilibration of the structure on the NMR time scale. This observation is consistent with the molecular flexibility of pentacoordinate antimony­(V) species, which display relatively low barriers to structural reorganization.^28,58,59^

**Scheme 2:**
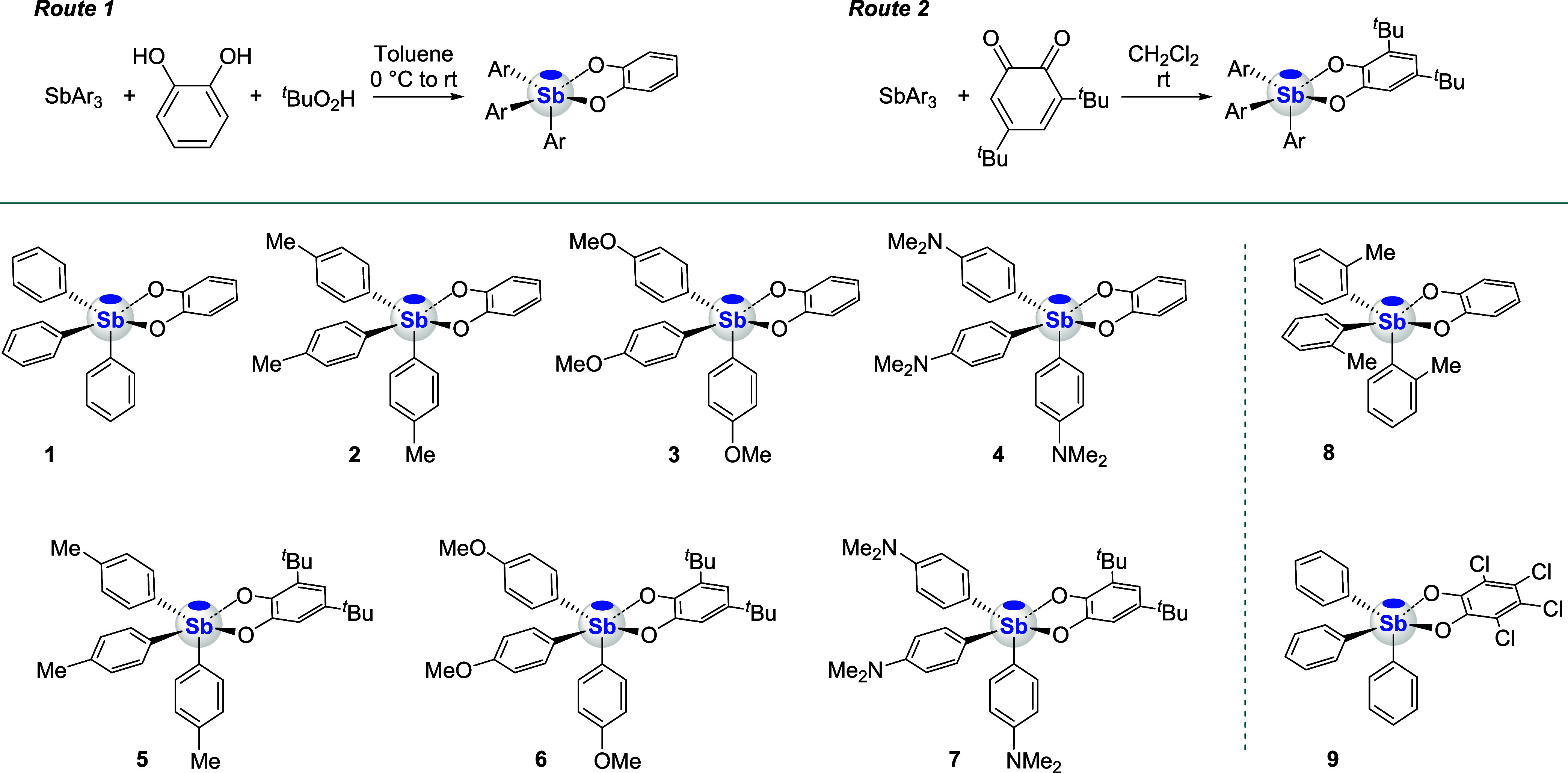
Synthesis and Structures of the Catecholatostiboranes Considered in This Study

All compounds, with the exception of **3** and **5**, were further characterized by single-crystal X-ray diffraction. The structures of compounds **1** and **6** are depicted in Figure 2 as illustrative examples, while the structures of the remaining compounds are available in SI. In the solid state, the coordination geometry of the antimony atom is intermediate between square pyramidal and trigonal bipyramidal, as indicated by the crystallographically determined structural parameter τ_5_, which spans the range of 0.12–0.67.^60^ Derivatives featuring the bulkier 3,5-di-*tert*-butyl catechol are closer to the trigonal bipyramidal geometry, as revealed by a comparison of the τ_5_ value of **7** (0.58) and **4** (0.12).

**Figure 2:**
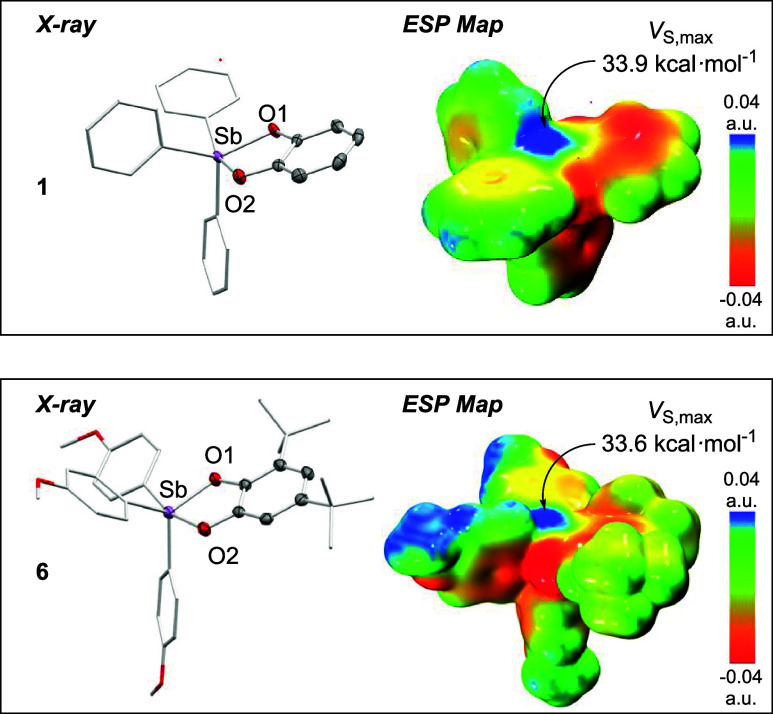
Single-crystal X-ray diffraction structures of **1** (top) and **6** (bottom). Hydrogen atoms have been omitted for clarity. The corresponding electrostatic potential maps, calculated in the fluoride-accepting geometries, are also shown.

### Pnictogen Bond Donor Properties

To assess the Lewis acidity of the antimony center and test its ability to engage with Lewis bases via pnictogen bond formation, we attempted the isolation of the corresponding triethylphosphine oxide (TEPO) adducts. Crystallization experiments carried out using a 1:1 ratio of the stiborane and the phosphine oxide were successful in the case of **1**, **5**, and **6** (Scheme 3), allowing for the structural characterization of the corresponding adducts **1**-TEPO, **5**-TEPO, and **6**-TEPO. The solid-state structure of **1**-TEPO is presented in Figure 3, while those of **5**-TEPO and **6**-TEPO are available in the SI. These adducts display a distorted octahedral geometry about the antimony center, with the oxygen from the phosphine oxide engaged in a pnictogen bond trans to one of the aromatic rings, a site corresponding to the location of the σ-hole, as documented for other Sb­(V) species.^18,61^ The Sb–O_TEPO_ bond of **1**-TEPO 2.2210(18) Å is significantly longer than that measured for (*o*-C_6_Cl_4_O_2_)­(C_6_F_5_)_3_Sb-TEPO (**10**-TEPO, 2.107(2) Å) which possesses a more Lewis acidic antimony center as a result of ligand perhalogenation.^61^ This simple comparison shows that the electronic characteristics of the ligands in these species may be leveraged to adjust the Lewis acidity or pnictogen bond donor properties of the antimony center. This notion is also supported by a comparison of **5**-TEPO (2.2797(11) Å), and **6**-TEPO (2.307(3) Å), with the latter featuring a longer Sb–O_TEPO_ bond, reflecting the more electron-donating features of the *p*-methoxy substituent vs the *p*-methyl substituent.

**Figure 3:**
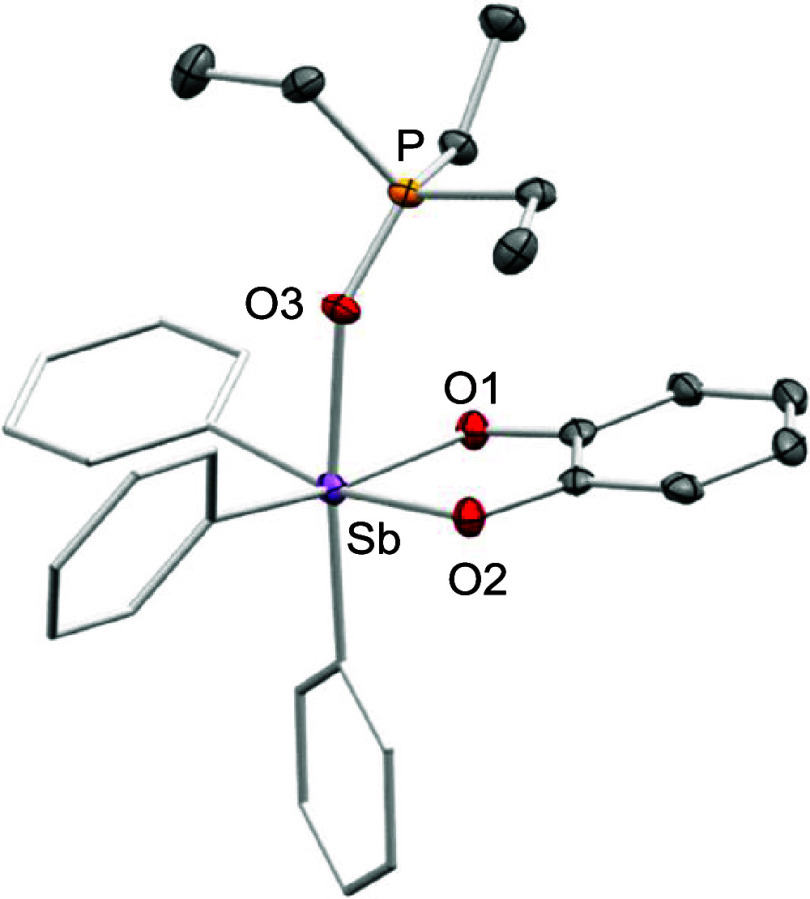
Solid-state structures of **1**-TEPO adduct, hydrogen atoms have been omitted for clarity.

**Scheme 3:**
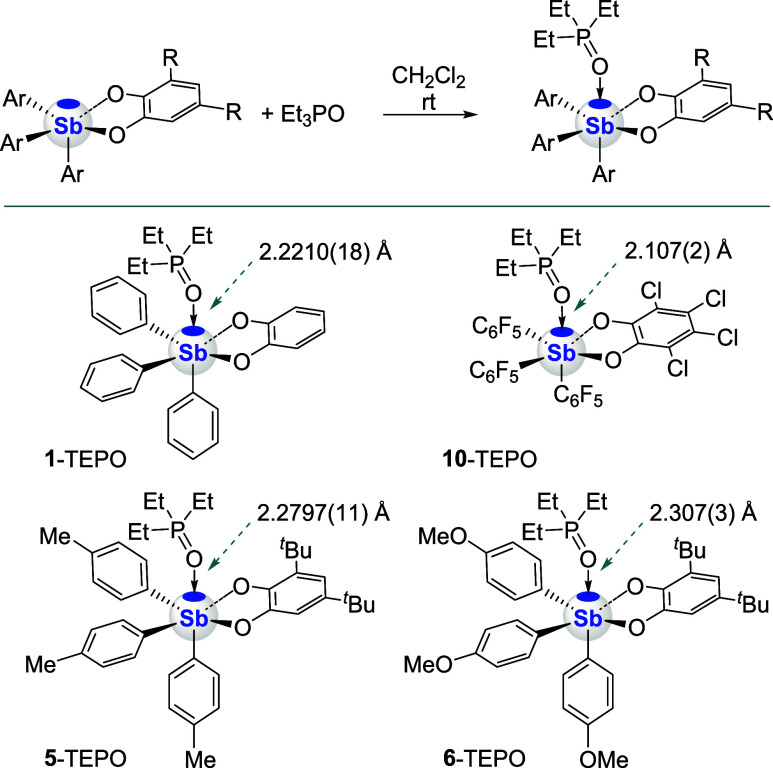
Interaction of the Stiboranes and Triethylphosphine Oxide (Top); Chemical Structures of the Resulting Stiboranes-TEPO Adducts with Respective Sb–O_TEPO_ Bond Lengths (Bottom)

Additional insight into the pnictogen bond donor properties of the catecholatostiboranes was derived from application of the Gutmann-Beckett (GB) method, based on ^31^P NMR measurements carried out on samples containing the stiborane and TEPO in equal concentration (0.1 M).^62^ The resulting downfield shifts (Δ^31^P), with respect to free TEPO, induced by the presence of the stiboranes, are compiled in Table 1. Given the known limitations of the GB method,^18,63^ we complemented these results by determining the TEPO binding constant (*K*_a_) using a reverse titration experiment also monitored by ^31^P NMR spectroscopy.^18^ Figure 4 shows the NMR data obtained with **1** along with the corresponding 1:1 binding isotherm, while all *K*_a_ values are provided in Table 1. The Δ^31^P and *K*_a_ parameters show the expected correlation, with both decreasing along the series **1**–**4** as the aryl ligand becomes more electron-releasing. These effects are also accounted for in the properties of the electrostatic potential maps of these derivatives, which show that the *V*_S,max_ values associated with the σ-hole responsible for pnictogen bonding generally decrease along the same series. The same trend is observed for **5**–**7**, further supporting the influence of subtle electronic effects on the pnictogen bond properties of the stiboranes. Another important conclusion concerns the role of steric effects as evinced by a comparison of the Δ^31^P and *K*_a_ values of stiboranes **2**–**4** to those of stiboranes **5**–**7**. The lower Δ^31^P and *K*_a_ measured for **5**–**7** show that the introduction of bulky tert-butyl groups on the catechol ligand dampens the binding of TEPO. Steric effects become even more evident in the case of **8**, which does not seem to perturb the chemical shift of TEPO. We will note in passing that a similar limitation was met for **7**, which possesses electron-donating aryl ligands and a sterically encumbered 3,5-di-*tert*-butyl-catecholate. The *K*_a_ values, which span a three-order magnitude range, provide a more contrasted illustration of the Lewis acidity differences observed between the various stiboranes. Finally, the steric effects implicated in the above discussion are readily captured by simple buried volume calculations, which show a clear difference between the catecholate (av. %*V*_Bur_ = 52.1) and 3,5-di-*tert*-butyl-catecholate derivatives (av. %*V*_Bur_ = 54.2). The effective blocking of the coordination site in **8** is consistent with the elevated %*V*_Bur_ value of 59.7.

**Figure 4:**
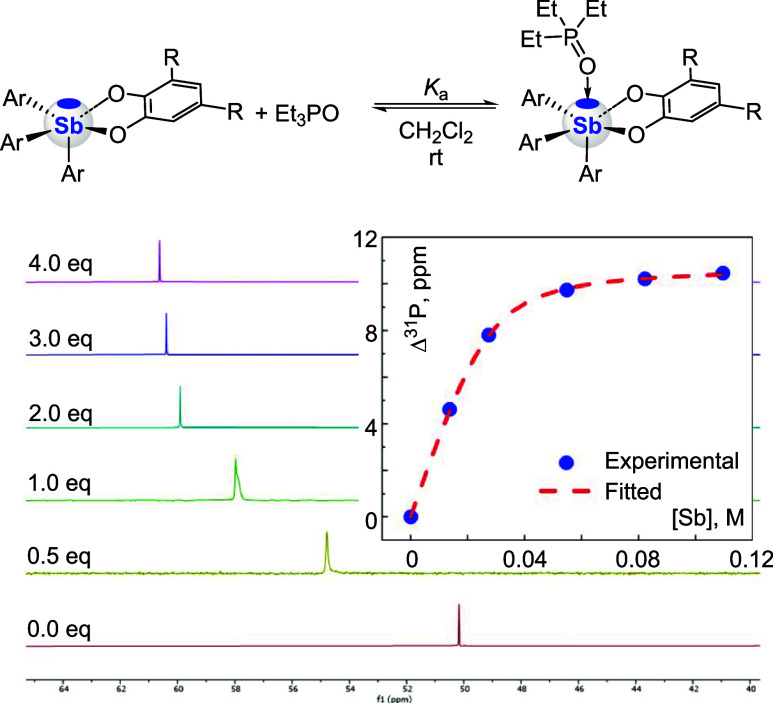
Changes in the ^31^P NMR spectra of TEPO (2.70 × 10^–2^ M) observed upon incremental addition of **1**. The inset shows the corresponding 1:1 binding isotherm fitted with *K*_a_ = 330 ± 18 M^–1^.

**Table 1:** Lewis Acidity Measurement: Δ^31^P, TEPO Binding Constants (*K*_a_), Computed Percent Buried Volume (%*V*_Bur_), and Computed Maximum Surface Electrostatic Potentials (*V*_S,max_)

compound	Δ^31^P (ppm)^a^	*K*_a_ (M^–1^)	%*V*_Bur_	*V*_S,max_ (kcal·mol^–1^)
**1**	9.4	330	52.2	33.9
**2**	7.6	65	52.2	32.9
**3**	7.2	63	52.1	33.1
**4**	1.4	2	52.1	26.2
**5**	4.1	13	54.4	32.4
**6**	3.6	7	54.4	33.6
**7**	0.2	^b^	53.7	24.0
**8**	0.0	^b^	59.7	36.3
**9**	12.4	>1000	53.0	47.8

a Determined using a 1:1 stiborane/TEPO mixture (0.1M) at 298 K.

b The TEPO binding constants are too small and cannot be determined by using the same experimental setup.

### Catalytic Properties

All antimony compounds were evaluated as potential catalysts for the copolymerization of cyclohexene oxide (CHO) and CO_2_, with bis­(triphenylphosphine)­iminium chloride (PPNCl) as a commonly used and very potent initiator (Scheme 4).^64^ These polymerization reactions were carried out by initially dissolving the stiboranes in neat CHO, followed by addition of PPNCl and subsequently CO_2_. In the absence of PPNCl and CO_2_, no immediate reaction was observed between the stiboranes and CHO, except in the case of **9** which quickly homopolymerized CHO. Using rigorously dried CHO led to similar results, suggesting that proton catalysis is not responsible for the observed reactivity. These initial results suggested that a platform with lower pnictogen bond donor properties may be better suited for the intended purpose of copolymerizing CO_2_ and CHO. With this in mind, we tested the simplest stiborane considered in this study, namely compound **1**, in neat CHO in the presence of PPNCl (one equiv) as an initiator under an atmosphere of CO_2_ at a pressure of 2.76 MPa and a temperature of 80 °C (Table 2, entry 1). Unlike with **9**, the reaction swiftly produced polycyclohexene carbonate (PCHC), validating the notion that weakened pnictogen bond donor properties favor the copolymerization reaction. All other stiboranes shown in Figure 2 also produced PCHC when evaluated under the same conditions, with the exception of **7** and **8**, which showed no activity (Table 2, entry 2-8). The lack of activity seen in the case of **8** is most easily rationalized by the steric blockage of the antimony center by the ortho methyl groups of the three aryl substituents. This lack of activity, which is readily correlated to the inability of this stiborane to engage with TEPO (*vide supra*), provides an important structure–property correlation that highlights the accessibility of the antimony center as a prerequisite for catalytic activity. To complete this initial survey, we revisited the activity of **9** in the presence of CO_2_. While **9** homopolymerized CHO, we found that combining it within a reactor with PPNCl and a premixed CHO/CO_2_ solution led to neither the formation of polyether nor polycarbonate (Table 2, entry 9). We propose that these results stem from the formation of an antimony-bound carbonate intermediate, the stability of which precludes turnover.

**Scheme 4:**
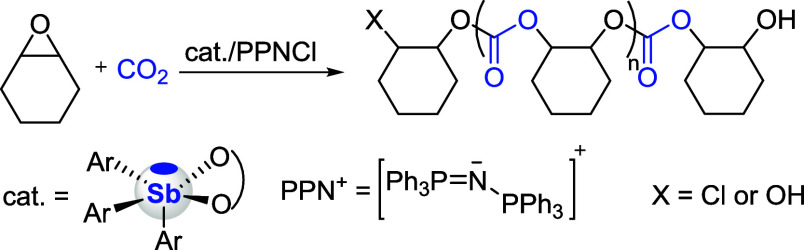
Copolymerization of CO_2_ and Cyclohexene Oxide Catalyzed by a Catecholatostiborane (cat.)

**Table 2:** Stiborane-Catalyzed CHO/CO_2_ Copolymerization

entry	catalyst	PCHC TON^a^	*k*_initial_ (10^–4^ s^–1^)^b^	*M_n_* (g·mol^–1^)^c^	*Đ* ^c^
1	**1**	869	2.15	7,771	1.28
2	**2**	810	3.20	14,312	1.17
3	**3**	1,150	3.83	14,898	1.14
4	**4**	583	3.48	13,516	1.23
5	**5**	1,258	4.79	20,995	1.14
6	**6**	1,134	5.48	19,440	1.14
7	**7**	0			
8	**8**	0			
9	**9**	0			

a Condition A: [CHO]/[catalyst]/[PPNCl] = 2000:1:1, 0.1 mL neat CHO, 3.1 MPa CO_2_, 90 °C, 12 h.

b Condition B: [CHO]/[catalyst]/[PPNCl] = 1000:1:1, 40 mL neat CHO, 2.76 MPa CO_2_, 80 °C.

c Determined by GPC on the isolated polymers from reaction condition B.

To more precisely assess the activity of the other stiboranes, we acquired time-dependent data using *in situ* FTIR spectroscopy, which provided us with initial rates (*k*_initial_) for each of the stiboranes (Table 2). We first carefully inspected the four stiboranes **1**–**4** and observed that the activity increases with the electron-donating properties of the aryl substituent until it becomes excessive in the case of **4**. These simple aryl substituent effects are quite marked, with the *k*_initial_ measured for **3** (3.83 × 10^–4^ s^–1^) almost double that of **1** (2.15 × 10^–4^ s^–1^). Another increase is observed when the catecholate ligand present in **2** and **3** is replaced by its 3,5-di-*tert*-butyl analog as in **5** and **6**, which feature notably higher *k*_initial_ of 4.79 × 10^–4^ s^–1^ and 5.48 × 10^–4^ s^–1^, respectively. These results suggest that further fine-tuning of the pnictogen bond donor properties via steric effect is beneficial. The lack of activity observed in the case of **7** speaks to the excessive electron-donating properties of the para-dimethylamino substituents, which entirely quench the pnictogen bond donor properties of the antimony center. This conclusion is corroborated by the lack of affinity that **7** displays for TEPO (*vide supra*). The turnover numbers and average molecular weight (*M_n_*) data generally scale with initial rate constants, reaching maxima in the case of **5** and **6**.

All copolymerizations yielded the desired polycyclohexene carbonate with bimodal distributions, a feature commonly observed in CO_2_/epoxide copolymerization^65–73^ and generally attributed to the presence of adventitious water.^74^ To test this assumption further, we examined whether residual moisture in CHO or the CO_2_ feed was responsible for the observed bimodal molecular weight distributions. The CHO used in this study was routinely dried by distillation over CaH_2_. To more firmly assert the dryness of the CHO, we carried out a set of experiments using CHO purified by distillation first over CaH_2_ and subsequently over *t*-butyllithium.^74^ The polymerization carried out with this batch of CHO, with compound **5** as a catalyst, afforded a similar initial rate (*k*_initial_ = 4.95 × 10^–4^ s^–1^ vs 4.79 × 10^–4^ s^–1^ for CHO dried over CaH_2_ only) and only a slightly higher *M_n_* (30,600 vs 26,767 g mol^–1^ for CHO dried over CaH_2_ only). Moreover, GPC analysis indicated an almost unchanged bimodality of the distribution. Based on these observations, which led us to conclude that water contamination of the CHO was negligible, we turned our attention to the CO_2_ feed employed. Drying the CO_2_ by bubbling the stream through neat Al­(^*i*^Bu)_3_ ^74^ and analyzing the resulting polymer by matrix assisted laser desorption ionization (MALDI) time-of-flight (TOF) mass spectrometry showed an increased fraction of the chloride-initiated polymer, which became the dominating component (see Figures S40 and S41). These results support the view that water acts as a chain-transfer agent in the copolymerization, and that traces of moisture in the CO_2_ feed, rather than in CHO, are responsible for the experimentally observed M_n_ values being lower than the theoretical values.

Even under unoptimized standard reaction conditions, the turnover numbers we achieved were comparable to those reported for trialkylboranes and metal­(salen) complexes in previous polymerization studies.^47,75,76^ Despite the bimodality of the polymers obtained in this study, the polydispersity remains narrow (1.14–1.28), and no induction period is observed in our kinetic experiments. Collectively, these results are consistent with the notion that polymerization is initiated by the chloride anion during the very early stage of the reaction. The presence of a chlorine atom as an end-group, as implied in Scheme 4, was confirmed by high-resolution MALDI TOF mass spectrometry. MALDI TOF also pinpoints the presence of hydroxide-initiated polymers, confirming the occurrence of water-induced chain transfer. The stereochemistry of the resulting PCHCs was investigated by ^13^C NMR spectroscopy. All copolymers exhibited a resonance at 153.8 ppm, corresponding to the carbonate carbon in isotactic tetrads, along with additional signals at 153.3, 153.2, and 153.1 ppm, which are characteristic of syndiotactic tetrads.^77^ The presence of both isotactic and syndiotactic signals suggested a lack of stereocontrol during the ring-opening step.

The best catalysts, **5** and **6**, were selected for additional investigations aimed at probing the effect of temperature and catalyst loading (Table 3). Irrespective of the loading, it is interesting to note that raising the temperature, from 60 to 90 °C, does not, in general, induce a large change in turnover number. This situation is, however, altered when the reaction temperature is raised to 120 °C, as shown by the data collected with a catalyst loading of 4000:1. In both cases, the TON approaches values in excess of 1500, making these stiboranes comparable or superior to the organoborane catalysts reported in the literature.^47,78^ Finally, we also tested the oxygen tolerance of such catalysts. When the assay in entry 2 was repeated with the addition of 50 psi of air to the vessel, the formation of the polycarbonate polymer was largely unperturbed, as confirmed by the TON of 618 obtained in this experiment. When the same assay was carried out with BEt_3_ as a catalyst and PPNCl as an initiator, no polycarbonate was observed, presumably because of the decomposition of BEt_3_ upon reaction with oxygen.^79^ These comparative results highlight the oxygen tolerance of these new antimony-based catalysts, a property that sets them apart from alkylborane systems. Given that O_2_ is often a contaminant in industrial CO_2_ streams, the robustness of the antimony catalysts herein reported stands as a notable advantage. We also compared the most active catalyst **5** uncovered in this work with two well-known catalysts, namely (*R*,*R*)-*N*,*N*’-bis­(3,5-di-*tert*-butylsalicylidene)-1,2-cyclohexanediaminocobalt­(III) chloride **11** ^80^ and (*R*,*R*)-*N*,*N*’-bis­(3,5-di-*tert*-butylsalicylidene)-1,2-cyclohexanediaminochromium­(III) chloride **12**,^77^ which have been extensively studied for the copolymerization of CO_2_ and epoxides.^64,69,77,80–83^ The results of these comparative studies, shown in Figure 5, indicate that stiborane **5** approaches the activity of the chromium catalyst **12** and significantly exceeds that of the cobalt catalyst **11**. These comparative studies shine a positive light on the antimony systems developed in this work and show that their catalytic performance can rival that of well-established transition metal systems.

**Figure 5:**
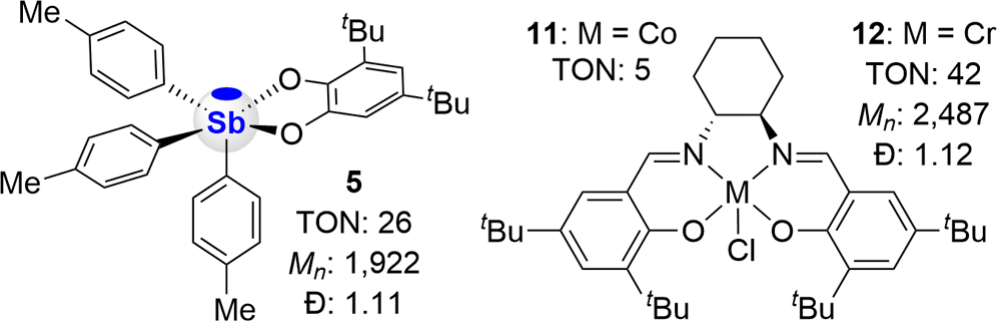
Comparison of stiborane **5** with benchmark catalysts **11** and **12**. Conditions: [CHO]/[catalyst]/[PPNCl] = 100/1/1, 0.1 mL neat CHO, 0.122 MPa CO_2_, 80 °C, 3 h. Catalyst **11** only gave low-molecular-weight oligomers which could not be analyzed by MALDI-TOF.

**Table 3:** Copolymerization Data of CHO/CO_2_ under Various Conditions^a^

entry	catalyst	[CHO]/[Cat]	Temp. (°C)	TON	*M_n_* (g·mol^–1^)^b^	*Đ* ^b^
1	**5**	1000:1	60	634	18,810	1.11
2	**5**	1000:1	90	660	17,559	1.13
3	**5**	2000:1	90	1,258	19,949	1.13
4	**5**	4000:1	60	683	5,782	1.14
5	**5**	4000:1	90	1,408	11,319	1.17
6	**5**	4000:1	120	1,506	8,145	1.12
7	**6**	1000:1	60	663	19,111	1.14
8	**6**	1000:1	90	590	16,715	1.22
9	**6**	2000:1	90	1,134	18,352	1.15
10	**6**	4000:1	60	814	6,284	1.26
11	**6**	4000:1	90	929	9,060	1.17
12	**6**	4000:1	120	1,564	5,969	1.36

a Conditions: [CHO]/[catalyst]/[PPNCl] = 2000:1:1, 0.1 mL neat CHO, 0.122 MPa CO_2_, 90 °C, 12 h.

b Determined by GPC on the isolated polymers.

### Proposed Polymerization Mechanism

Since this work marks the first observation of stiboranes functioning as catalysts in the copolymerization of CO_2_ and epoxides, mechanistic investigations were considered using **5**, one of the two best catalysts identified in this study. The polymerization rate exhibited a first-order dependence on catalyst concentration when varying the loading from 0.025 to 0.1 mol % (Figure 6a). Since the cocatalyst was maintained in a 1:1 stoichiometric ratio with the stiborane, the reaction kinetics should be considered first-order with respect to the combined stiborane/cocatalyst concentration. Further, we examined the polymerization rate under different CO_2_ pressures ranging from 0.69 to 5.17 MPa. Unlike many reported organocatalytic CO_2_/epoxide copolymerization systems, which typically exhibit zero-order dependence on CO_2_ pressure,^84^ our system showed a continuous increase in rate with rising CO_2_ pressure (Figure 6b). The rate dependence in CO_2_ is unusual, especially since saturation kinetics were not reached, even at 5 MPa of CO_2_ pressure. Importantly, we observed first-order rate dependence in CHO in all the variable CO_2_ pressure kinetic experiments, consistent with the notion that CHO is involved in the rate-determining step. This observation suggests that a rapid CO_2_ insertion equilibrium existed between catalyst-bound alkoxide and catalyst-bound carbonate species. The kinetic data were successfully fitted into our simplified mechanistic model, allowing the estimation of the CO_2_ insertion equilibrium constant *K*_eq_ as 0.53 MPa^–1^ (See SI). Prior studies have suggested that catalysts with larger CO_2_ insertion equilibrium constants might perform more consistently at lower CO_2_ pressures and reach saturation at lower pressures, which aligns with our observations.^85^ Unlike these catalytic systems, where the carbonate species dominates at high CO_2_ pressures, our kinetic data indicate that this equilibrium does not entirely shift toward the carbonate form.

**Figure 6:**
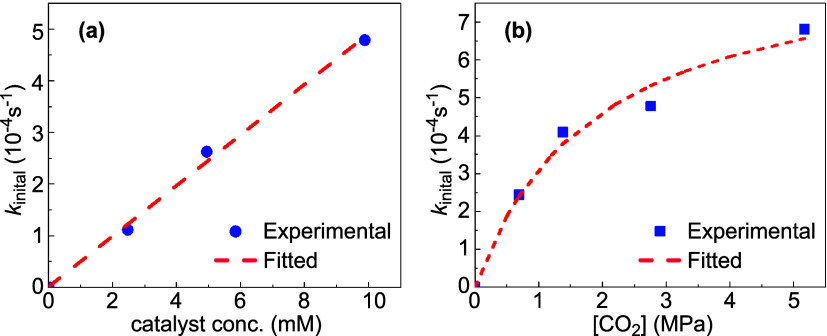
Plots used to determine the rate dependence on (a) the catalyst concentration, and (b) the CO_2_ pressure.

Based on these findings, we propose the mechanism for CO_2_/CHO copolymerization shown in Figure 7. Polymerization is initiated via nucleophilic attack by chloride ion (Cl^–^) on the epoxide coordinated to the antimony center, generating a catalyst-bound alkoxide intermediate (T1). This alkoxide undergoes CO_2_ insertion, forming a catalyst-bound carbonate species (T2), and establishing a rapid equilibrium between these two species. The polymerization cycle proceeds as the catalyst-bound carbonate species reacts with an incoming epoxide, facilitating ring-opening and chain propagation. While our findings support the presence of a CO_2_ insertion equilibrium within the catalytic cycle, the precise mode of interaction between the antimony center and the polymer chain has not yet been unambiguously determined. However, it appears clear that the best catalysts identified in this study are those with weaker pnictogen bond donor properties, as in the case of **5** and **6** whose Lewis acidity is tamed by relatively electron-rich aryl ligands and remote steric bulk imposed by the *t*-butyl groups at the 3- and 5-position of the catecholate ligand. The correlation observed between the activity of the catalysts and their pnictogen-bond donor properties may result from the reduced propensity of these stiboranes to form overly stable Lewis adducts with either the alkoxide or carbonate end group, as suspected in the case of **9**. Forming such thermodynamically stable species would potentially lead to a higher activation energy for the rate-determining, ring-opening step.

**Figure 7:**
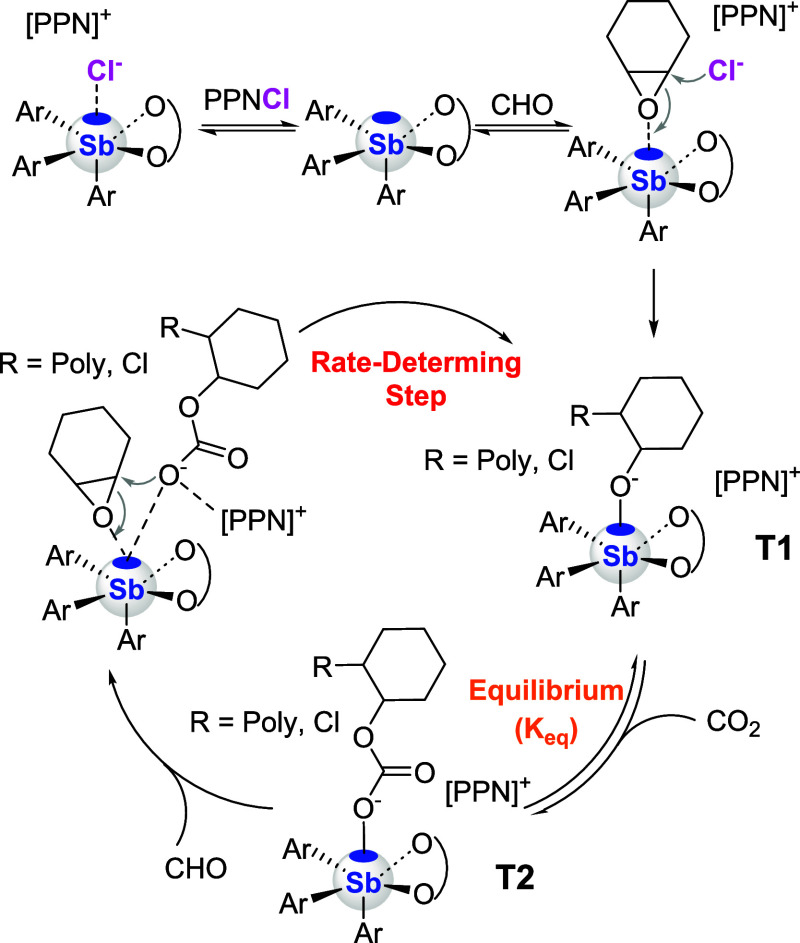
Proposed mechanism for the copolymerization of CO_2_ and CHO catalyzed by a stiborane in the presence of PPNCl.

To investigate the proposed intermediates experimentally, we selected complex **1** as a representative catalyst. Combining CHO with PPNCl in a 1:1 ratio at 60 °C did not produce a detectable chloro-alkoxide species. Similarly, no reaction was observed between CHO and complex **1** alone under the same conditions. However, when CHO was treated with a 1:1:1 mixture of complex **1** and PPNCl, new resonances appeared in the ^1^H NMR spectrum, corresponding precisely to those of 2-chlorohexanol or the corresponding alkoxide.^86^ The formation of this species supports the relevance of a stiborane-bound alkoxide species of type T1 (R = Cl) during the early stage of the reaction.

## Conclusions

In this work, we have investigated a series of triaryl antimony­(V) catecholates as pnictogen bonding catalysts for the copolymerization of CO_2_ and CHO. By strategically modifying their electronic and steric properties, we were able to elucidate the relationship between the pnictogen bond donor predisposition of these systems and their catalytic efficiencies. Compared to alkylboranes, these antimony­(V) compounds not only offer greater modularity but also exhibit enhanced stability in the presence of oxygen, a ubiquitous impurity that poisons trivalent borane catalysts. Altogether, our study establishes stiborane-based pnictogen bonding catalysis as a highly tunable approach for the copolymerization of CO_2_ and epoxides, offering a new platform for group 15 catalysis in polymer chemistry.^87–90^

## Methods

### General Procedure for the Preparation of the Catecholatostiboranes

Method 1: Under a nitrogen atmosphere, the desired triarylstibine was treated with catechol (1 equiv) in toluene (10 mL). The resulting solution was cooled to 0 °C using an ice bath. After 15 min of stirring at this temperature, an aqueous solution of *tert*-butyl hydroperoxide (70 wt %, 1.3 equiv) was slowly added over the course of 30 min. The reaction temperature was maintained at 0 °C and the solution was stirred for an additional 2 h. The solvent was then removed under vacuum, affording a residue that was dissolved in CH_2_Cl_2_ and purified by column chromatography, using CH_2_Cl_2_ as the mobile phase and silica as the stationary phase. Method 2: Under a nitrogen atmosphere, the triarylstibine (1 equiv) was dissolved in CH_2_Cl_2_ (5 mL). After stirring for 5 min, the resulting solution was combined with 3,5-di-*tert*-butyl-*o*-benzoquinone (1 equiv) and stirred for an additional 30 min. The addition of hexanes (20 mL) led to the precipitation of the product, which was isolated by filtration and washed with hexanes (5 mL).

### Evaluation of Triethylphosphine Oxide Binding Constants

The Et_3_PO binding constants were determined by a reverse titration experiment in which a solution of Et_3_PO was titrated with increasing amounts of the stiborane. These experiments were carried out by combining an Et_3_PO stock solution (100 mL, 134 mM) with a stock solution of the stiborane. In each case, the volume of the resulting sample was adjusted to 0.5 mL through the addition of pure CH_2_Cl_2_. A capillary containing DMSO-*d*_6_ was inserted in each sample to provide an NMR lock signal. The equilibrium constant *K*_a_ was obtained by fitting the ^31^P NMR chemical shifts to a 1:1 binding isotherm.

### Procedure Adopted for the Polymerization Experiments

The polymerization of CO_2_ and CHO were performed in a stainless-steel vessel. A desired quantity of the catalyst was first dissolved in 100 μL of CHO. The vessel was pressurized with 3.10 MPa CO_2_, isolated, and heated to the respective temperatures for 12 h. The reaction was then brought back to ambient temperature and depressurized. The reaction mixture was then dissolved in 1 mL of CDCl_3_ containing 1,3-bis­(trimethylsilyl)­benzene (5 mM) as an internal standard for quantification. The turnover number (TON), corresponding to the number of epoxides converted into polymers per catalyst, was determined by ^1^H NMR spectroscopy. The polymers were isolated by drying in a vacuum oven. The molecular weights were determined by GPC.

### FTIR Monitoring of the Polymerization Reactions

The copolymerization of CO_2_ and CHO was measured using a ReactIR system (Mettler-Toledo LLC, Columbus, OH) equipped with an Attenuated Total Reflection (ATR) inserted into the base of a Parr reactor. The probe was connected to an FTIR spectrometer, allowing for the *in situ* measurements of CO_2_\epoxide polymerization. The FTIR spectra were collected from 650 to 3500 cm^–1^, with a wavenumber spacing of 4 cm^–1^.

### Computational Methods

Density functional theory (DFT) structural optimizations were performed with the Gaussian 16 program.^91^ In all cases, the crystal structure geometries were optimized using the B3LYP functional^92,93^ and the following mixed basis sets: aug-cc-pVTZ-PP^93–95^ for Sb, 6–31G­(d′)^96,97^ for F, and 6–31G^98,99^ for C, O, N, and H. For all optimized structures, frequency calculations were performed in order to confirm the absence of imaginary frequencies. Single point calculations carried out at the optimized geometry with the B3LYP functional and the following mixed basis sets: aug-cc-pVTZ-pp for Sb and 6–311+g­(2d,p) for C, H, O, and N. The enthalpies used to derive the FIA were obtained by single-point calculations carried out at the optimized geometry. These calculations employed the B3LYP functional and the following mixed basis sets: aug-cc-pVTZ-pp for Sb and 6–311+g­(2d,p) for C, H, O, N, and F. The enthalpy correction term was taken from the above-mentioned frequency calculations.

## Supplementary Material



## Notes

Synthetic, experimental and computational details, characterization data and optimized structures in xyz format (PDF)
