# Supplementary material for: Pnictogen-Bonding Catalysis: Copolymerization of CO2 and Epoxides on Antimony(V) Platforms
Source: ACS Catal. 2025 Oct 15;15(21):17882–92. doi: 10.1021/acscatal.5c03781 (PMC12603998; doi:10.1021/acscatal.5c03781)
Supplement: Supplementary file 1 [file cs5c03781_si_001.pdf]

Supplementary Information

Pnictogen-bonding catalysis: Copolymerization of CO_2 and epoxides on antimony(V) platforms

Chenggang Jiang,[a] Eryn Lee,[b] Jonathan Schaefer,[b] Matthew W. Holtcamp,[b] Tzu-Pin Lin,[b] and François P. Gabbaï*[a]*

[a]Department of Chemistry, Texas A&M University, College Station, TX 77843, United States.
[b]ExxonMobil Technology and Engineering Company, Baytown, Texas 77520, United States.

Corresponding authors' emails: francois@tamu.edu; tzu-pin.lin@exxonmobil.com

Table of Contents

1 Synthesis and Characterization S2
1.1 General Considerations S2
1.2 Synthetic Procedures S2
1.3 NMR Spectra S4
1.4 X-ray Crystallographic Details and Solid State Structures S12
2 Complexation of Et_3PO by the Stiboranes. S16
3 Stiborane-Catalyzed Copolymerization Study S21
3.1 CO_2/Epoxide Copolymerization Measured by FTIR Spectroscopy in a Parr Reactor S21
3.2 Initial Rate Method of Analyzing the Polymerization Rate S24
3.3 First-Order Direct Fitting Method of Analyzing the Polymerization Rate S25
3.4 CO_2 Polymerization Experiments (Small Scale Screening) S26
3.5 Estimation of *K*eq of Based on Our Mechanism Model. S27
3.6 Polymer Characterization S30
4 Computation Studies S37
4.1 Methods S37
4.2 Percent Volume Buried (%V_{Bur}). S37
4.3 Electrostatic Potential Maps and $V_{S,max}$ Calculations S37
4.4 Optimized Structures and Coordinates of Stiborane Compounds S38
4.5 Topographic Steric Maps of Stiborane Catalyst S63
4.6 Visualized Electrostatic Potential Maps. S68
5 References S71

1 Synthesis and Characterization

1.1 General Considerations

All chemicals were purchased from commercially available sources and were used without further purification unless otherwise specified. All solvents were ACS reagent grade and used as received. The tris(*p*-methoxyphenyl)stibine, tris(*p*-tolyl))stibine, tris(*o*-tolyl))stibine, tris(4-*N*,*N*-dimethylaminophenyl)stibine, compound **1**, and **9** were synthesized according to literature procedures with spectra that matched those reported.[1-4] The NMR spectra of compounds **1** and **9** are provided to document their purity. All NMR spectra were recorded at room temperature using an in-house Bruker Ascend 400 NMR spectrometer. The ^{1}H and $^{13}C\{^{1}H\}$ signals were referenced to residual solvent signals. The MALDI-TOF spectra were collected using an in-house Bruker microflex MALDI-TOF spectrometer.

1.2 Synthetic Procedures

General procedure A for the preparation of the catecholatostiboranes.

Under a nitrogen atmosphere, 1 equiv. of the triarylstibine was treated with catechol (1 equiv.) in toluene (10 mL). The resulting solution was cooled to 0 °C using an ice bath. After 15 min of stirring at this temperature, an aqueous solution of *tert*-butyl hydroperoxide (70 wt.%, 1.3 equiv.) was slowly added over the course of 30 min. The reaction, maintained at 0 °C, was stirred for an additional two hours. The solvent was then removed under vacuum, affording a residue that was dissolved in CH_2Cl_2 and purified by silica column chromatography, using CH_2Cl_2 as the mobile phase.

General procedure B for the preparation of the catecholatostiboranes.

Under a nitrogen atmosphere, the triarylstibine (1 equiv.) was dissolved in CH_2Cl_2 (5 mL). After stirring for 5 min, the resulting solution was combined with 3,5-di-*tert*-butyl-*o*-benzoquinone (1 equiv.) and stirred for an additional 30 min. The addition of hexanes (20 mL) led to the precipitation of the product, which was isolated by filtration and washed with hexanes (5 mL).

Synthesis of 2. This complex was prepared according to general procedure A using tri(*p*-tolyl)antimony (1.0 g, 2.5 mmol) as starting material. The product was obtained as a yellow solid. Yield: 0.94 g (74%). ^{1}H NMR (400 MHz, $CDCl_3$): δ 7.67 (d, J = 8.1 Hz, 6H, tolyl-*H*), 7.31 (d, J = 8.0 Hz, 6H, tolyl-*H*), 6.98 (dd, J = 5.8, 3.5 Hz, 2H, Catechol-*H*), 6.69 (dd, J = 5.7, 3.6 Hz, 2H, Catechol-*H*), 2.42 (s, 9H, Ar-CH_3). $^{13}C\{^{1}H\}$ NMR (101 MHz, $CDCl_3$): δ 147.9, 141.6, 135.0, 134.2, 130.1, 118.4, 112.4, 21.6. Bulk purity was determined to be over 98% by NMR spectroscopy.

Synthesis of 3. This complex was prepared according to general procedure A using tris(*p*-methoxyphenyl)antimony (1.0 g, 2.3 mmol) as starting material. The product was obtained as a yellow solid. Yield: 0.68 g (55%). ^{1}H NMR (400 MHz, $CDCl_3$): δ 7.66 (d, J = 8.9 Hz, 6H, Ar-*H*), 6.98 (d, J = 8.8 Hz, 6H, Ar-*H*), 6.92 (dd, J = 5.8, 3.5 Hz, 2H, Catechol-*H*), 6.64 (dd, J = 5.8, 3.5 Hz, 2H, Catechol-*H*), 3.83 (s, 9H, Ar-OCH_3). $^{13}C\{^{1}H\}$ NMR (101 MHz, $CDCl_3$): δ 162.1, 147.9,

136.6, 128.2, 118.4, 115.0, 112.4, 55.4. Bulk purity was determined to be over 98% by NMR spectroscopy.

Synthesis of 4. This complex was prepared according to general procedure A using tris(4-N,N-dimethylaminophenyl)antimony (1.0 g, 2.1 mmol). The product was obtained as a light yellow solid. Yield: 0.88 g (72%). ^{1}H NMR (400 MHz, $CDCl_3$): δ 7.64 (d, *J* = 9.0 Hz, 6H, Ar-*H*), 6.94 (dd, *J* = 5.8, 3.5 Hz, 2H, Ar-*H*), 6.75 (d, *J* = 8.9 Hz, 6H, Catechol-*H*), 6.63 (dd, *J* = 5.8, 3.5 Hz, 2H, Catechol-*H*), 2.99 (s, 18H, Ar-N(CH_3)$_2$). ^{13}C{^{1}H} NMR (101 MHz, $CDCl_3$): δ 147.9, 141.6, 135.0, 134.2, 130.1, 118.4, 112.4, 21.6. Bulk purity was determined to be over 98% by NMR spectroscopy.

Synthesis of 5. This complex was prepared according to general procedure B using tri(*p*-tolyl)antimony (1.0 g, 2.5 mmol). The product was obtained as a bright yellow solid. Yield: 1.1 g (71%). ^{1}H NMR (400 MHz, $CDCl_3$): δ 7.56 (d, *J* = 8.1 Hz, 6H, Ar-*H*), 7.17 (d, *J* = 8.2 Hz, 6H, Ar-*H*), 6.86 (d, *J* = 2.3 Hz, 1H, Quinone-*H*), 6.61 (d, *J* = 2.3 Hz, 1H, Quinone-*H*), 2.29 (s, 9H, Ar-CH_3), 1.37 (s, 9H, C(CH_3)$_3$), 1.20 (s, 9H, C(CH_3)$_3$). ^{13}C{^{1}H} NMR (101 MHz, $CDCl_3$): δ 146.9, 143.2, 141.2, 139.3, 135.0, 134.7, 133.1, 129.9, 112.3, 107.8, 34.7, 34.5, 31.8, 29.8, 21.5. Bulk purity was determined to be over 98% by NMR spectroscopy.

Synthesis of 6. This complex was prepared according to general procedure B using tris(*p*-methoxyphenyl)antimony (1.0 g, 2.3 mmol). The product was obtained as a yellow solid. Yield: 0.96 g (64%). ^{1}H NMR (400 MHz, $CDCl_3$): δ 7.73 (d, *J* = 8.9 Hz, 6H, Ar-*H*), 7.01 (d, *J* = 8.8 Hz, 6H, Ar-*H*), 6.98 (d, *J* = 2.3 Hz, 1H, Quinone-*H*), 6.73 (d, *J* = 2.4 Hz, 1H, Quinone-*H*), 3.85 (s, 9H, Ar-OCH_3), 1.50 (s, 9H, C(CH_3)$_3$), 1.32 (s, 9H, C(CH_3)$_3$). ^{13}C{^{1}H} NMR (101 MHz, $CDCl_3$): δ 161.9, 146.9, 143.3, 139.4, 136.7, 133.1, 128.9, 114.9, 112.4, 107.9, 55.4, 34.8, 34.6, 31.9, 29.9. Bulk purity was determined to be over 98% by NMR spectroscopy.

Synthesis of 7. This complex was prepared according to general procedure B using tris(4-N,N-dimethylaminophenyl)antimony (1.0 g, 2.1 mmol). The product was obtained as a light yellow solid. Yield: 1.1 g (75%). ^{1}H NMR (400 MHz, $CDCl_3$): δ 7.67 (d, *J* = 8.8 Hz, 6H, Ar-*H*), 6.96 (d, *J* = 2.4 Hz, 1H, Quinone-*H*), 6.75 (d, *J* = 8.9 Hz, 6H, Ar-*H*), 6.68 (d, *J* = 2.5 Hz, 1H, Quinone-*H*), 2.99 (s, 18H, Ar-N(CH_3)$_2$), 1.51 (s, 9H, C(CH_3)$_3$), 1.32 (s, 9H, C(CH_3)$_3$). ^{13}C{^{1}H} NMR (101 MHz, $CDCl_3$): δ 151.9, 147.5, 143.8, 138.7, 136.4, 132.7, 123.8, 112.3, 111.8, 107.8, 40.2, 34.8, 34.5, 32.0, 30.0. Bulk purity was determined to be over 98% by NMR spectroscopy.

Synthesis of 8. This complex was prepared according to general procedure A using tri(*o*-tolyl)antimony (1.0 g, 2.5 mmol). The product was obtained as a light yellow solid. Yield: 0.87 g (69%). ^{1}H NMR (400 MHz, $CDCl_3$): δ 7.54 (d, J = 6.2 Hz, 3H, Ar-*H*), 7.38 (t, *J* = 7.5 Hz, 3H, Ar-*H*), 7.30 (d, *J* = 6.6 Hz, 3H, Ar-*H*), 7.24 (t, *J* = 7.5 Hz, 3H, Ar-*H*), 6.91 (dd, *J* = 5.7, 3.6 Hz, 2H, Catechol-*H*), 6.67 (dd, *J* = 5.7, 3.5 Hz, 2H, Catechol-*H*), 2.39 (s, 9H, Ar-CH_3). ^{13}C{^{1}H} NMR (101 MHz, $CDCl_3$): δ 147.9, 142.7, 140.5, 133.5, 131.7, 131.0, 126.5, 118.4, 112.3, 23.8. Bulk purity was determined to be over 98% by NMR spectroscopy.

1.3 NMR Spectra

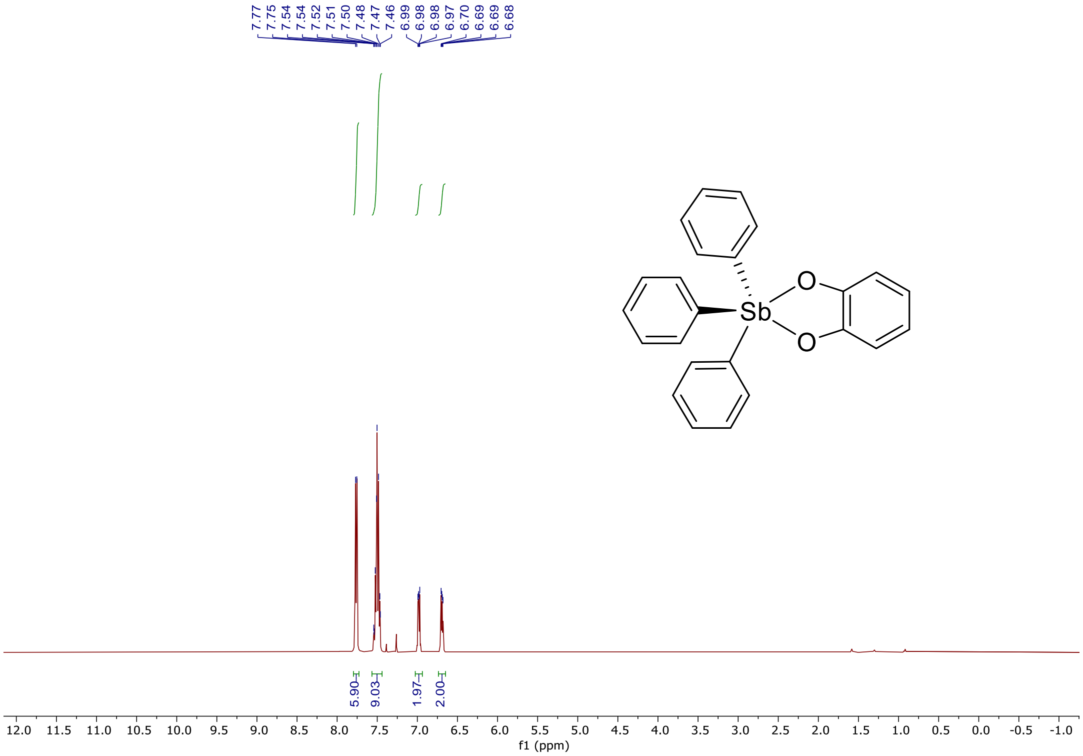

Figure S1. ^{1}H NMR spectrum of **1** recorded at 400 MHz in $CDCl_3$.

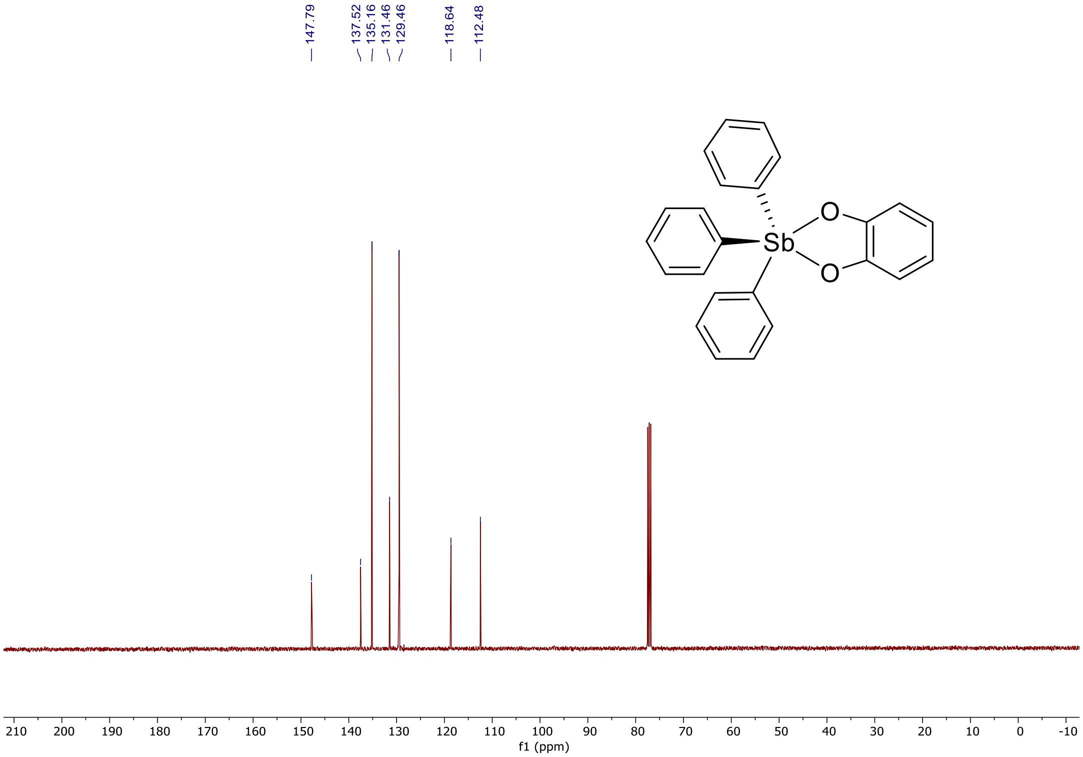

Figure S2. $^{13}C\{^{1}H\}$ NMR spectrum of **1** recorded at 101 MHz in $CDCl_3$.

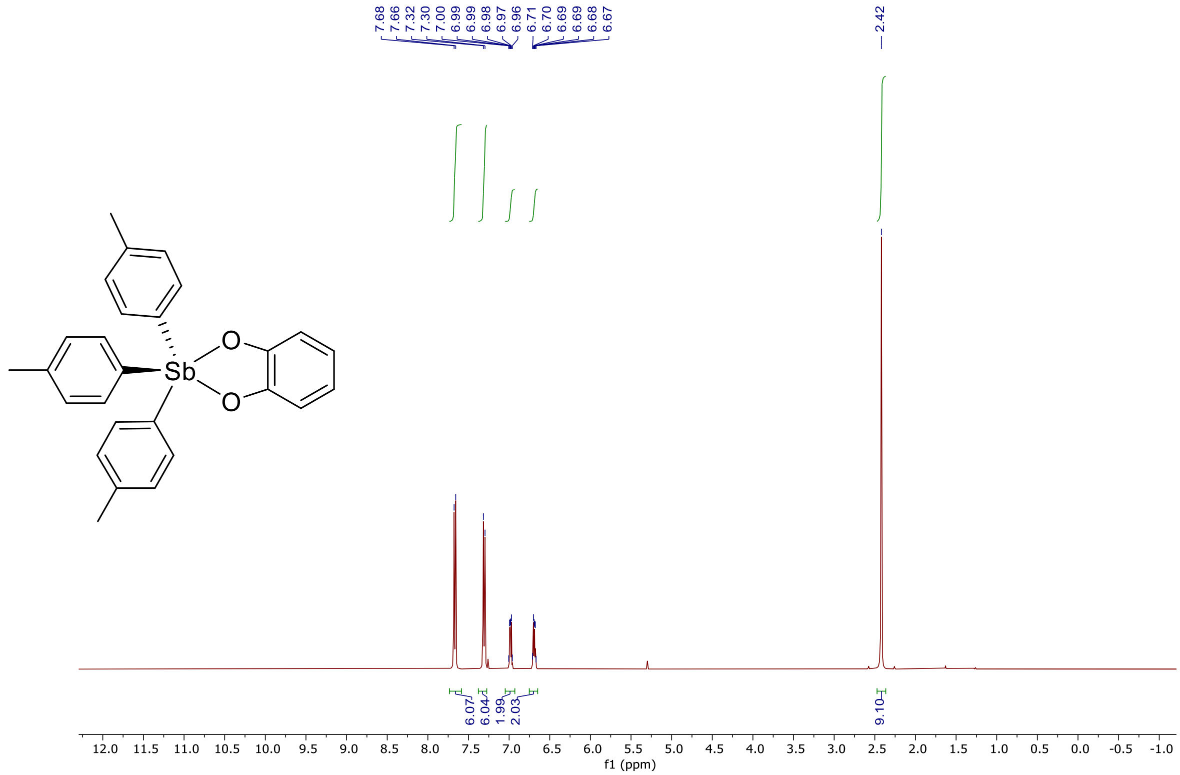

Figure S3. ^{1}H NMR spectrum of **2** recorded at 400 MHz in $CDCl_3$.

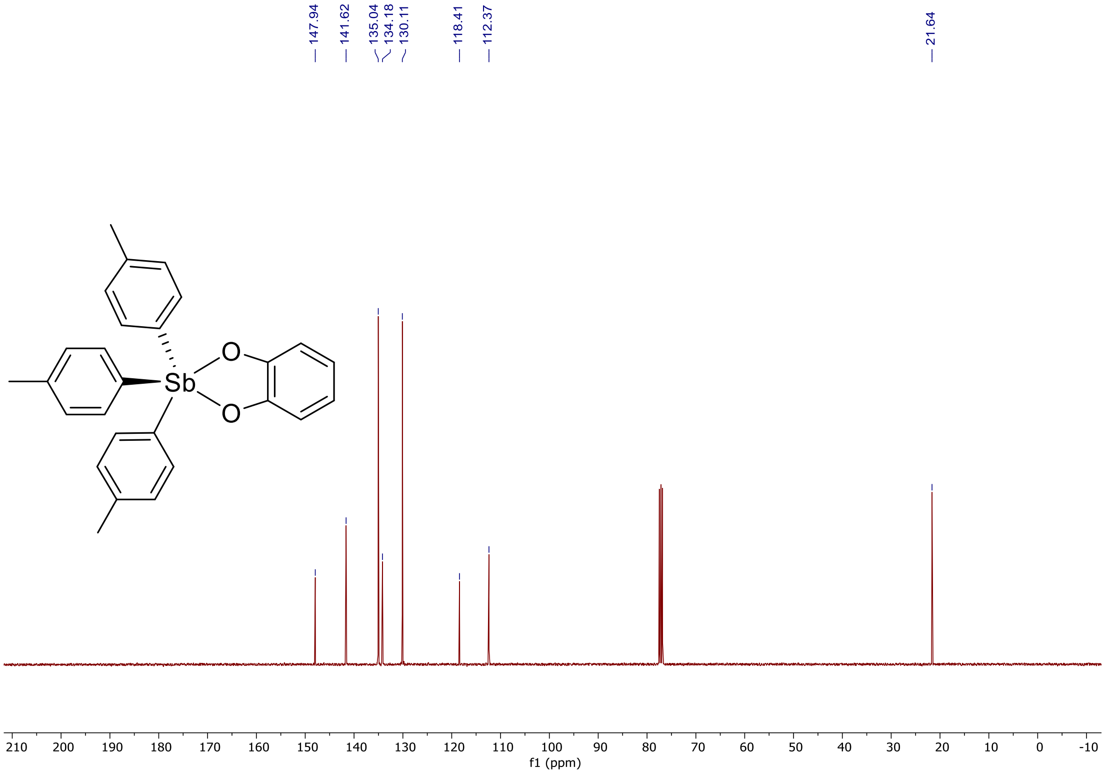

Figure S4. $^{13}C\{^{1}H\}$ NMR spectrum of **2** recorded at 101 MHz in $CDCl_3$.

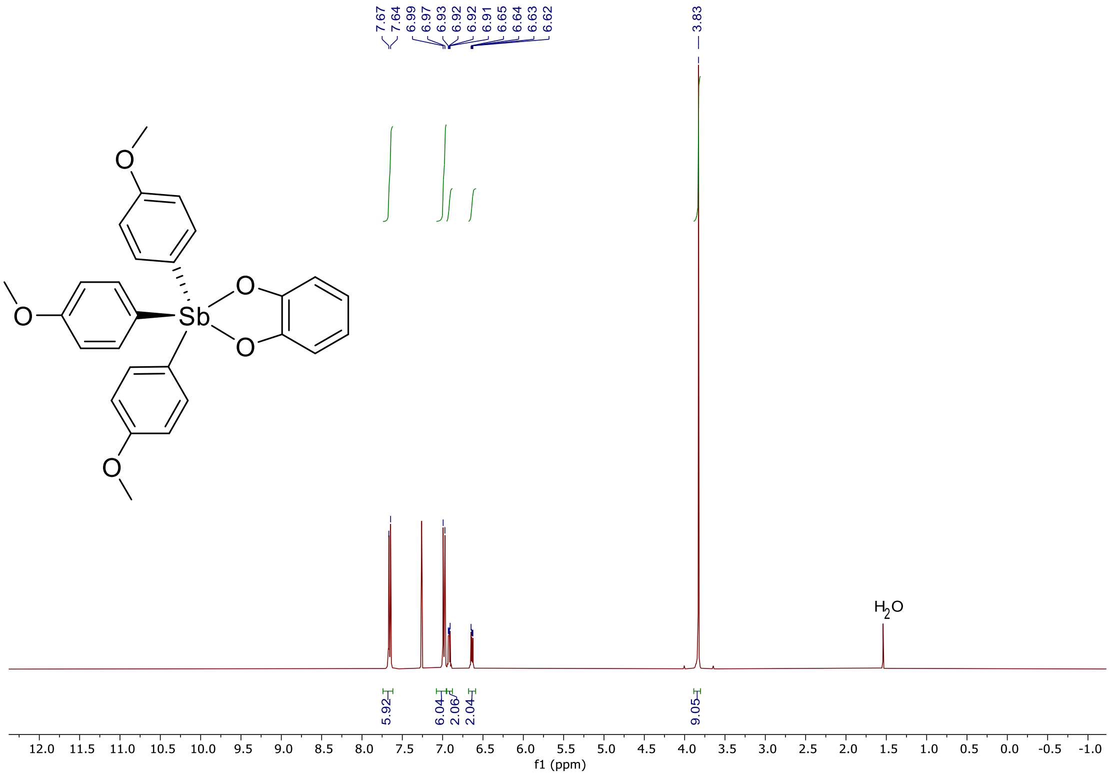

Figure S5. ^{1}H NMR spectrum of **3** recorded at 101 MHz in $CDCl_3$.

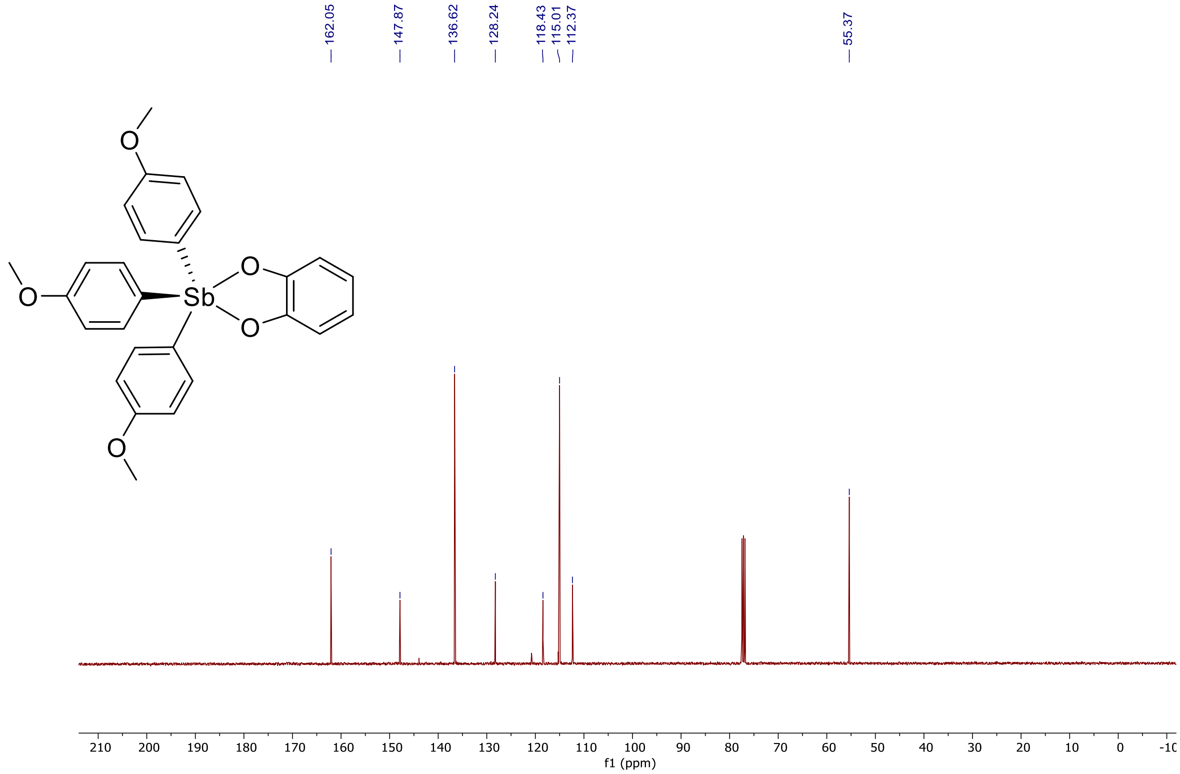

Figure S6. $^{13}C\{^{1}H\}$ NMR spectrum of **3** recorded at 101 MHz in $CDCl_3$.

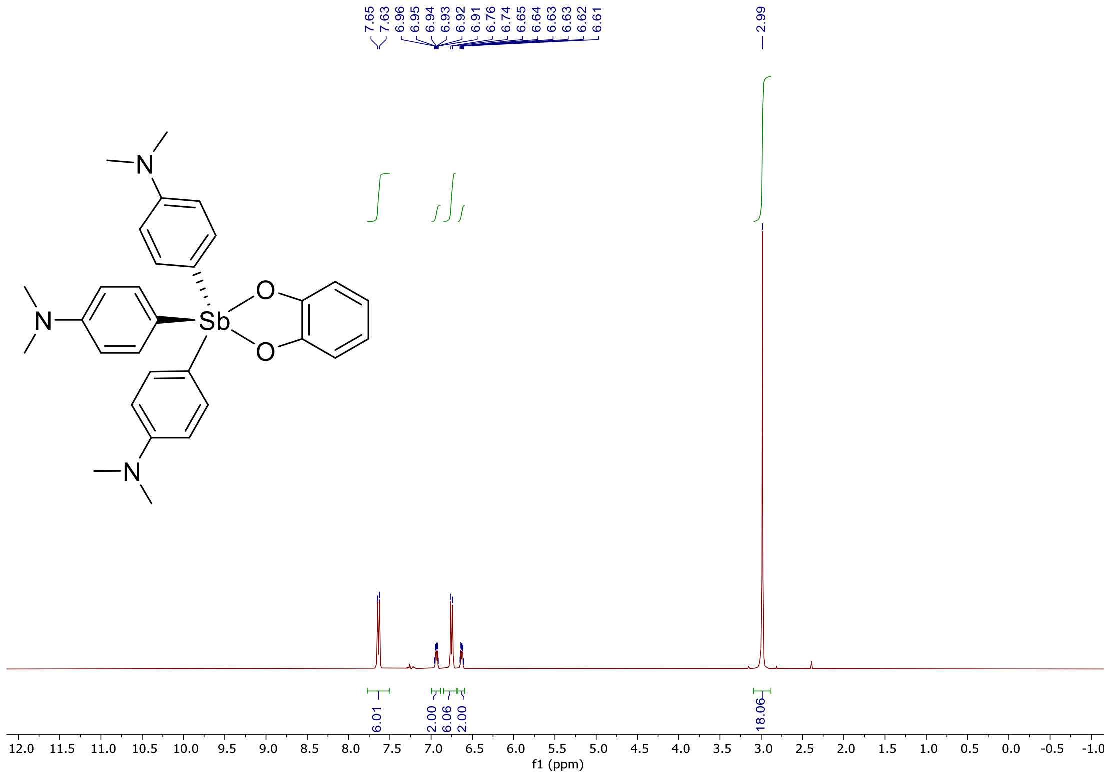

Figure S7. ^{1}H NMR spectrum of **4** recorded at 400 MHz in $CDCl_3$.

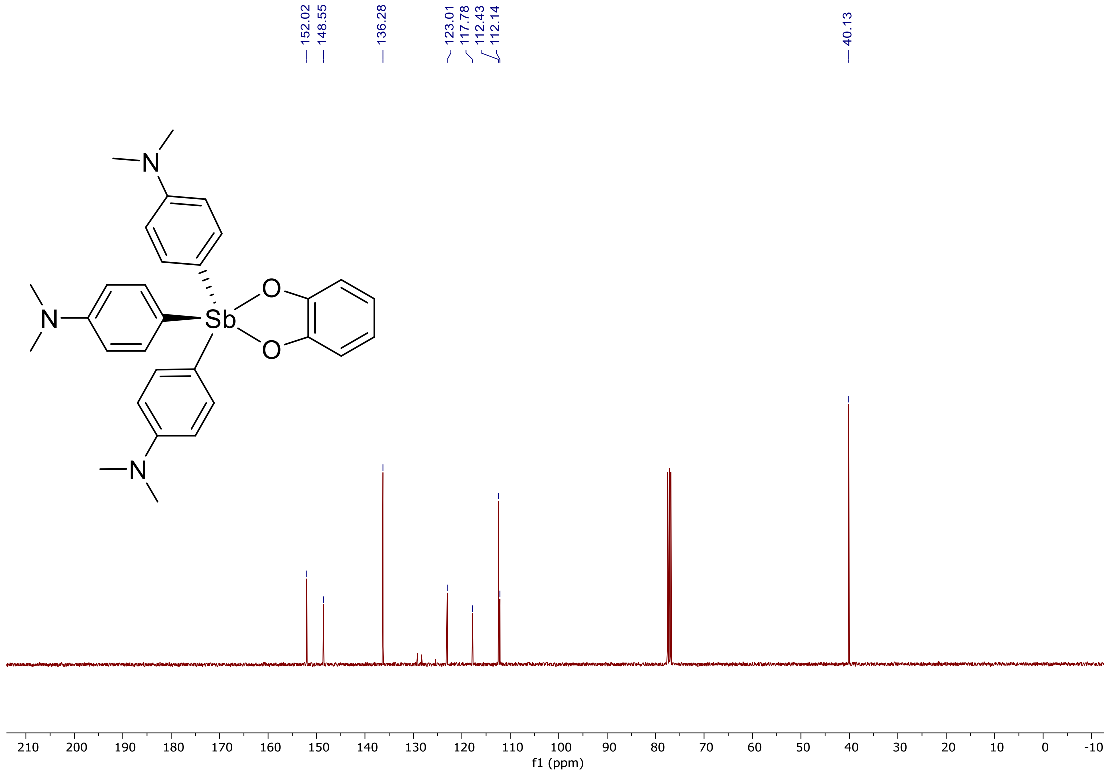

Figure S8. $^{13}C\{^{1}H\}$ NMR spectrum of **4** recorded at 101 MHz in $CDCl_3$.

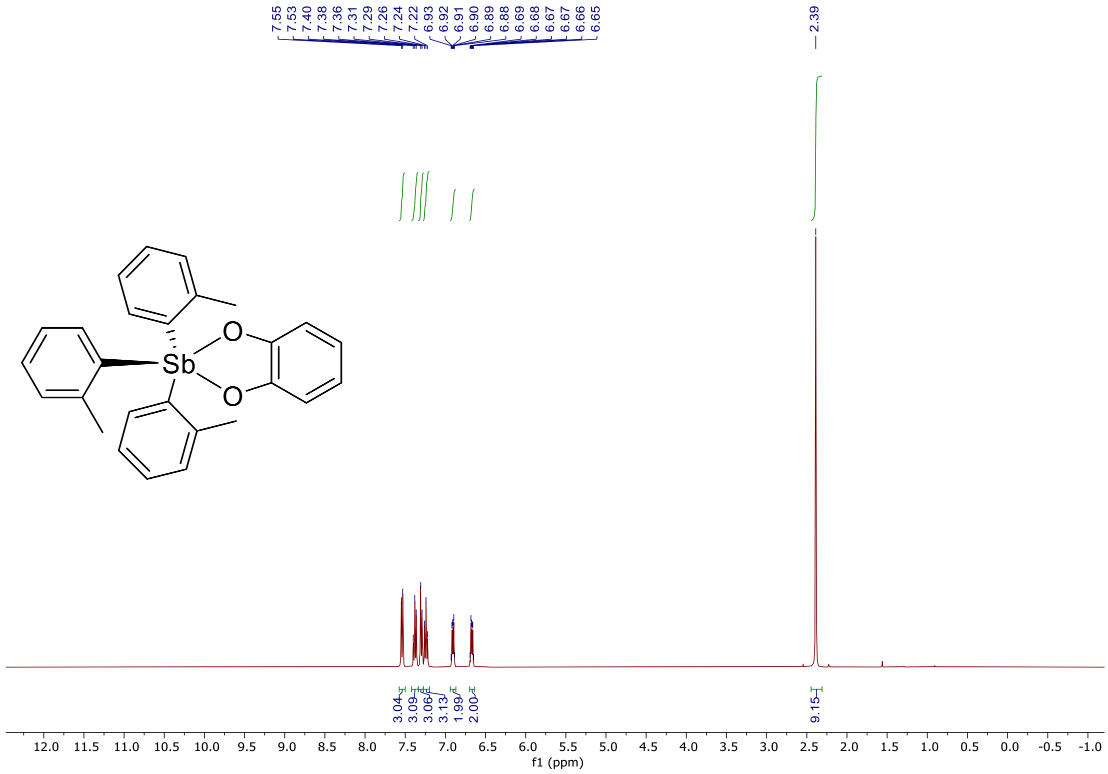

Figure S9. ^{1}H NMR spectrum of **8** recorded at 400 MHz in $CDCl_3$.

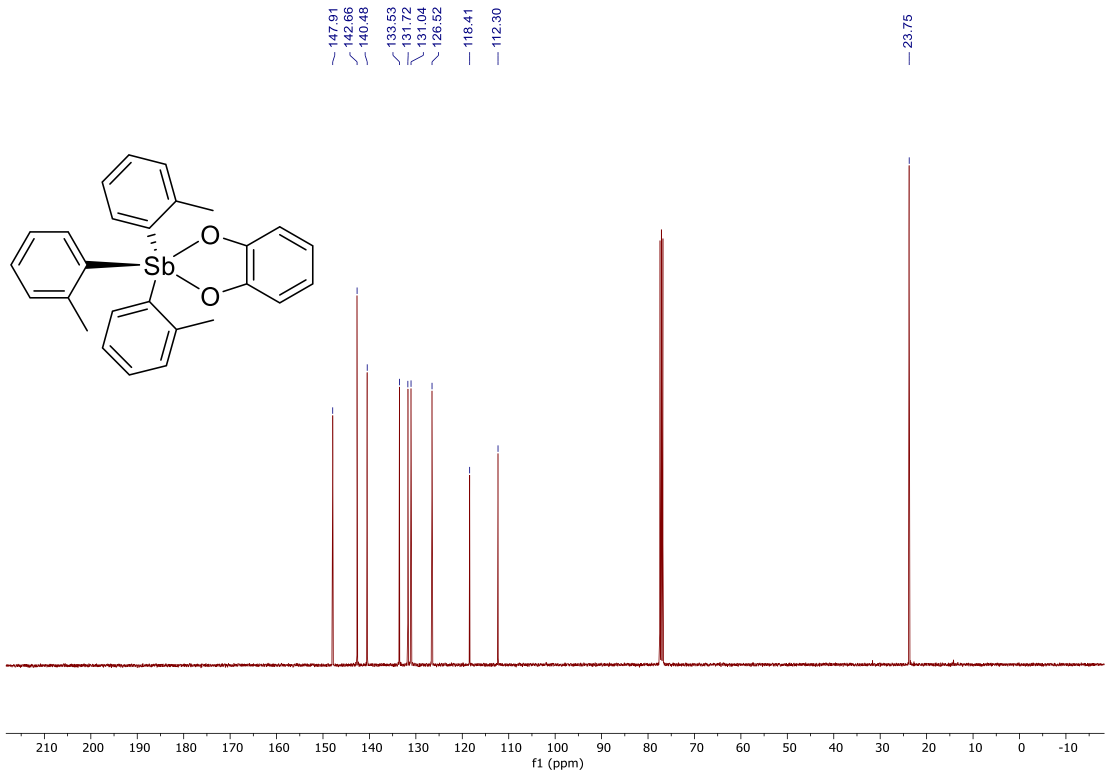

Figure S10. ^{13}C{^{1}H} NMR spectrum of **8** recorded at 101 MHz in $CDCl_3$.

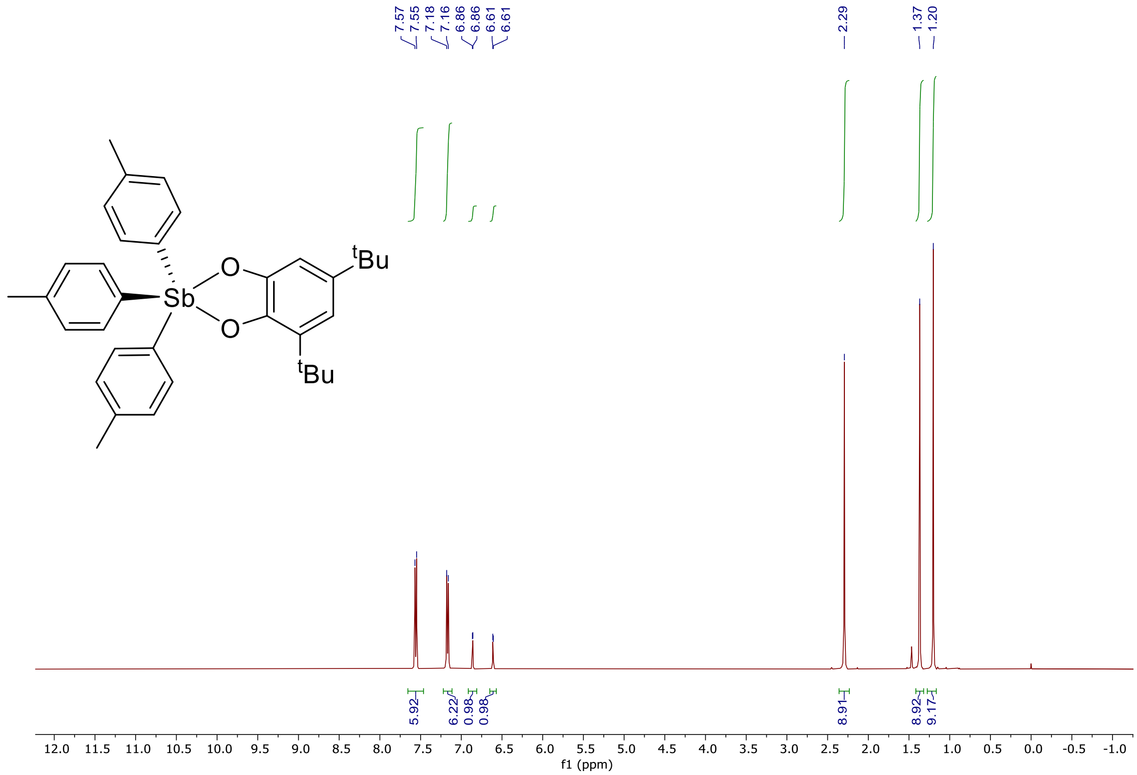

Figure S11. ^{1}H NMR spectrum of **5** recorded at 400 MHz in $CDCl_3$.

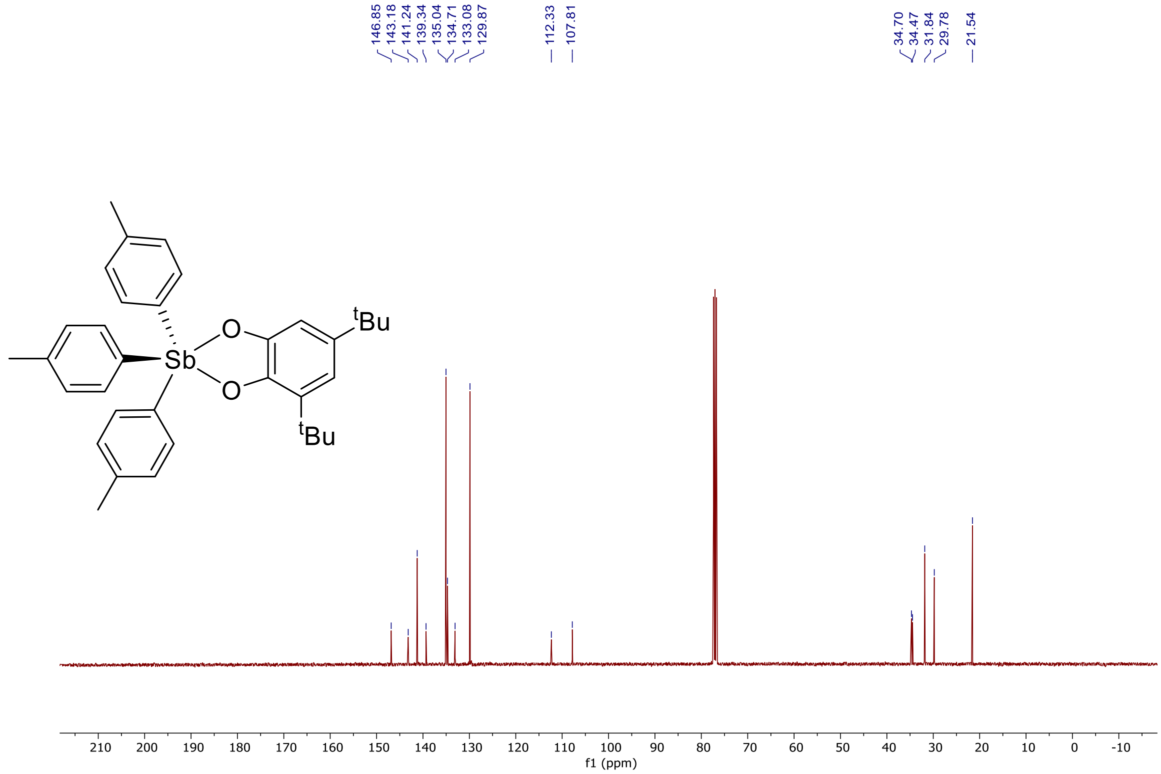

Figure S12. $^{13}C\{^{1}H\}$ NMR spectrum of **5** recorded at 101 MHz in $CDCl_3$.

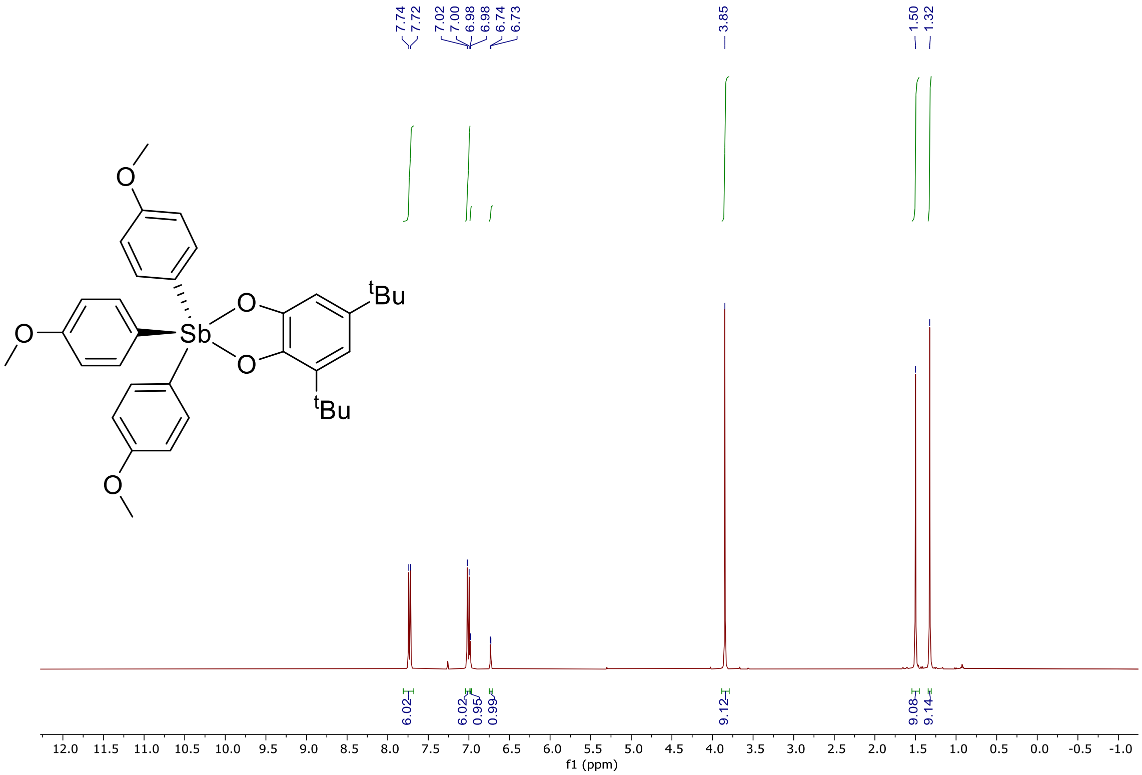

Figure S13. ^{1}H NMR spectrum of **6** recorded at 400 MHz in $CDCl_3$.

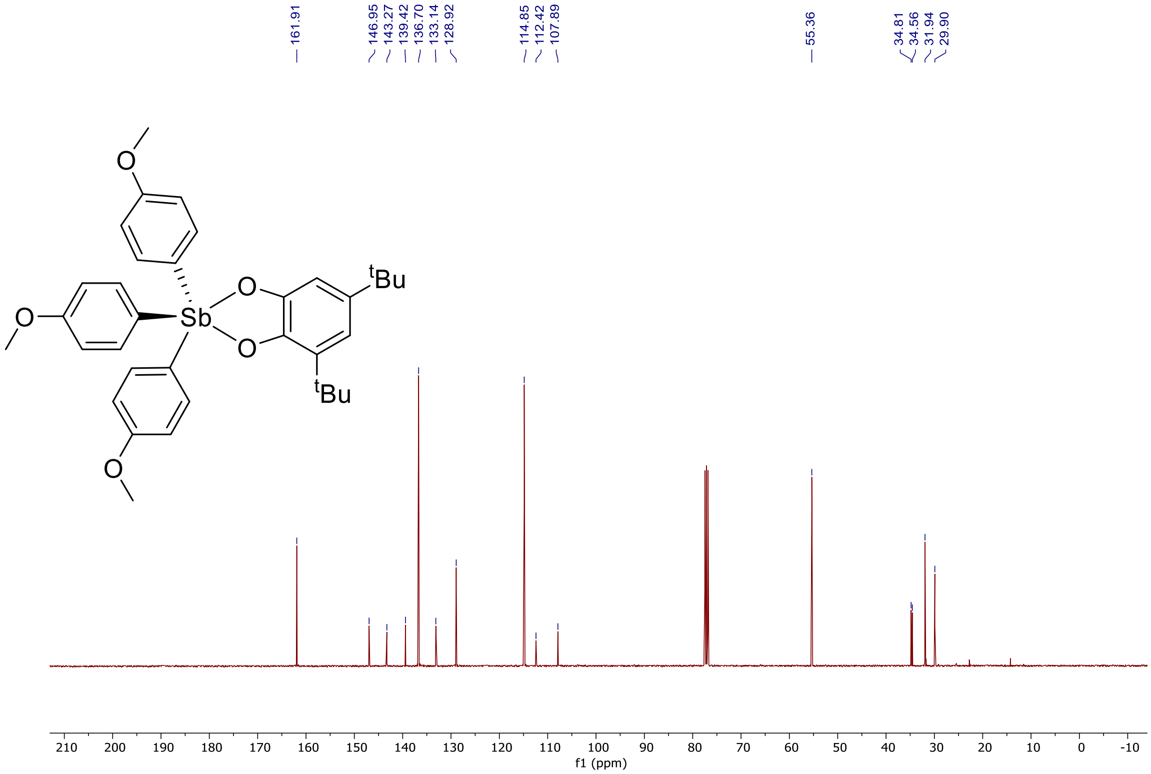

Figure S14. $^{13}C\{^{1}H\}$ NMR spectrum of **6** recorded at 101 MHz in $CDCl_3$.

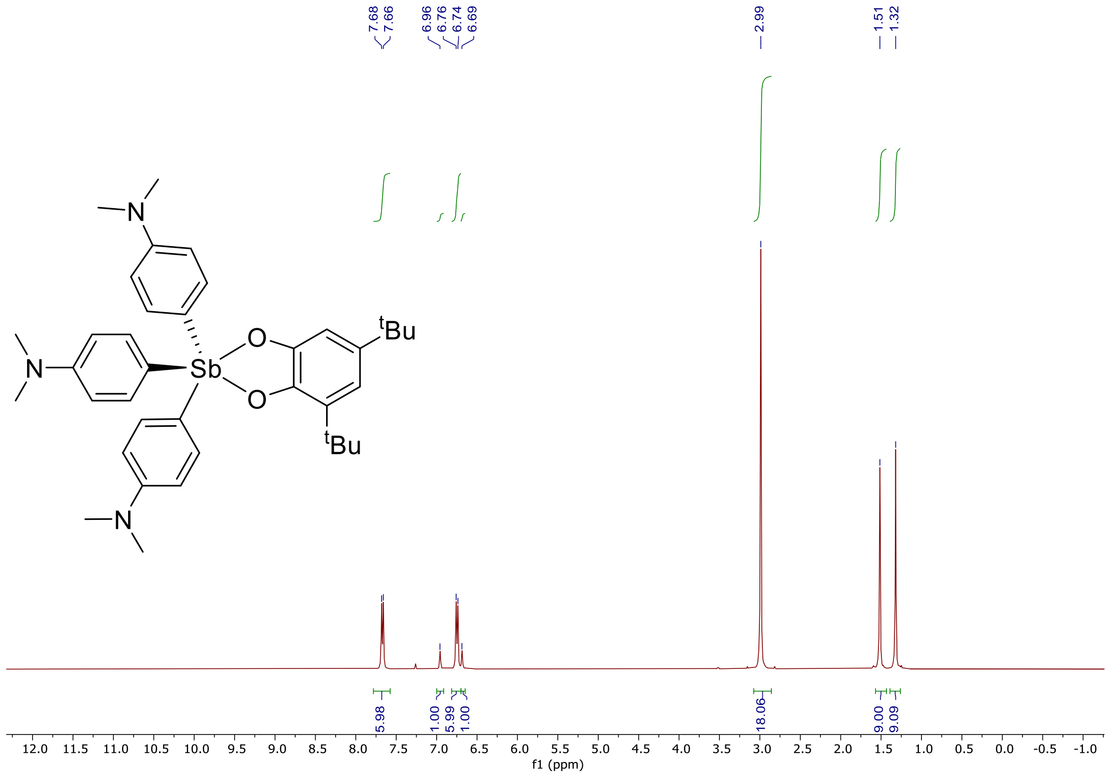

Figure S15. ^{1}H NMR spectrum of **7** recorded at 400 MHz in $CDCl_3$.

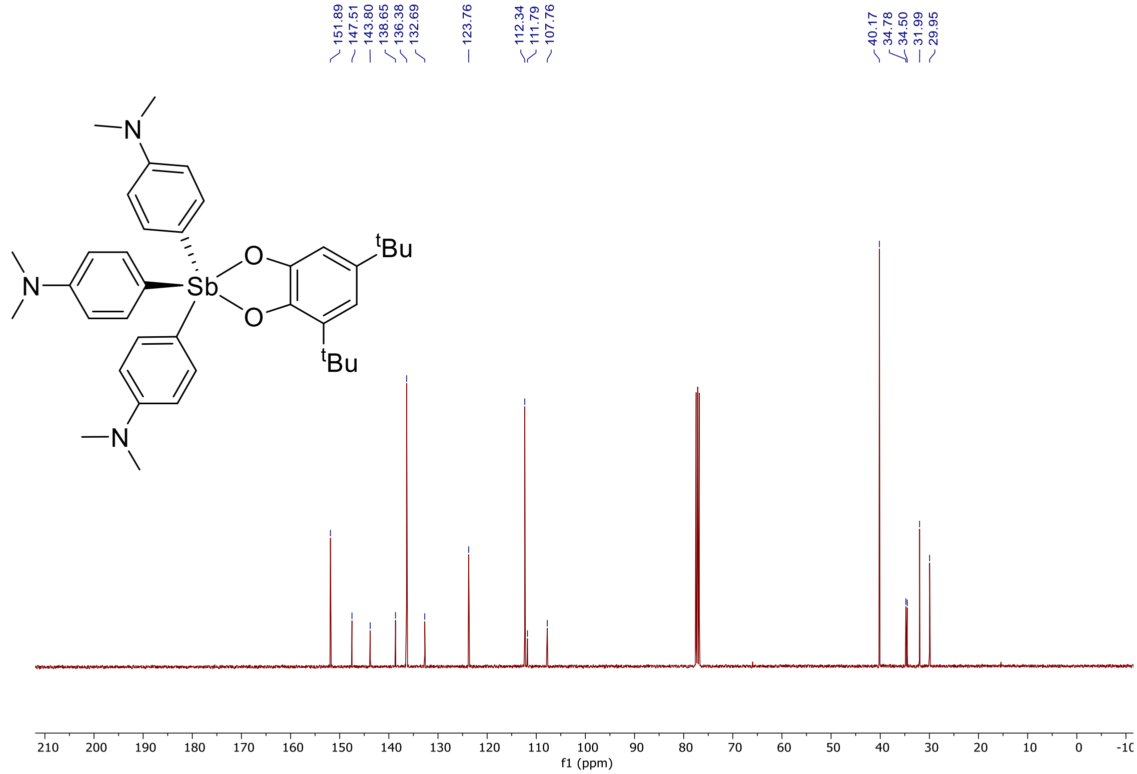

Figure S16. $^{13}C\{^{1}H\}$ NMR spectrum of **7** recorded at 101 MHz in $CDCl_3$.

1.4 X-ray Crystallographic Details and Solid State Structures

Single crystals for compounds **1**, **2**, **4**, **7**, and **8** were grown by layering pentane on nearly saturated $CHCl_3$ solutions of the corresponding derivatives. Single crystals of **1**-TEPO, **5**-TEPO, and **6**-TEPO were obtained by layering hexane on a 1:1 mixture of the corresponding stiborane and Et_3PO in CH_2Cl_2. Crystallographic measurements were performed at 100 K or 110 K using a Bruker D8 QUEST diffractometer (Mo-Kα radiation, λ = 0.71073 Å) or a Rigaku XtaLAB Synergy diffractometer (Mo-Kα radiation, λ = 0.71073 Å, or Cu-Kα radiation, λ = 1.5406 Å). In each case, a specimen of suitable size and quality was selected and mounted onto a MicroMounts cryo-loop. The data was corrected for absorption effects using the empirical method SADABS. The structures were solved by direct methods and refined using the SHELXTL[5] and OLEX2[6] software packages. Hydrogen atoms bonded to carbon were placed in calculated positions using the standard riding model and refined isotropically; all non-hydrogen atoms were refined anisotropically. Visualizations of crystal structures were prepared using the software Mercury. CCDC 2452436-2452444 contain the supplementary crystallographic data for this paper. These data are provided free of charge by The Cambridge Crystallographic Data Centre.

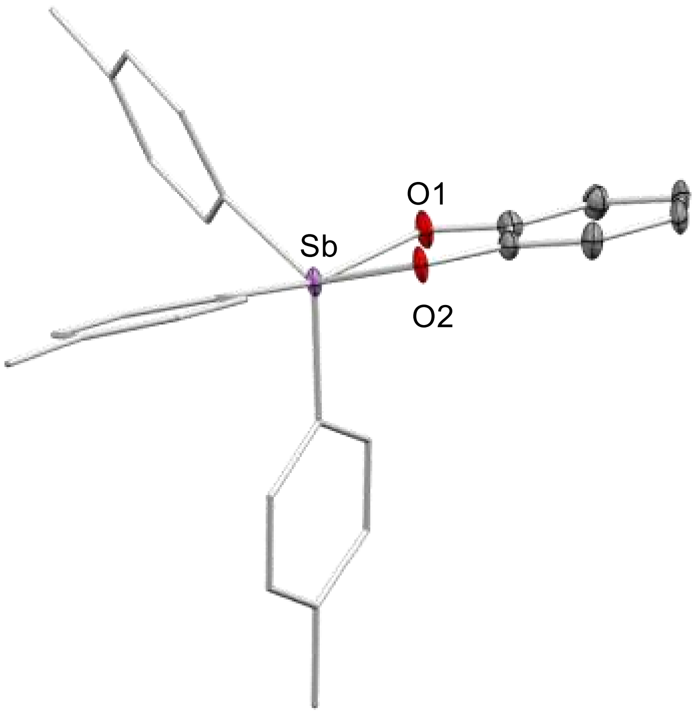

Figure S17. Molecular structure of **2** with hydrogen atoms omitted for clarity.

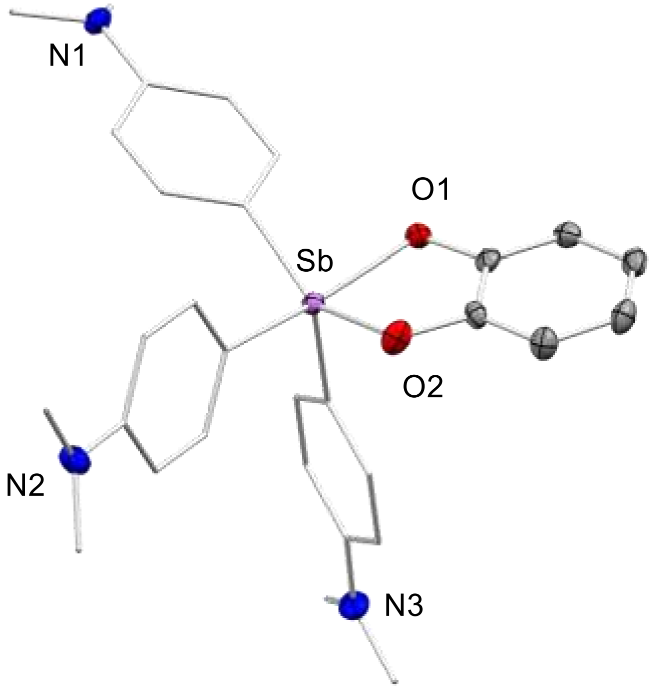

Figure S18. Molecular structure of **4** with hydrogen atoms omitted for clarity.

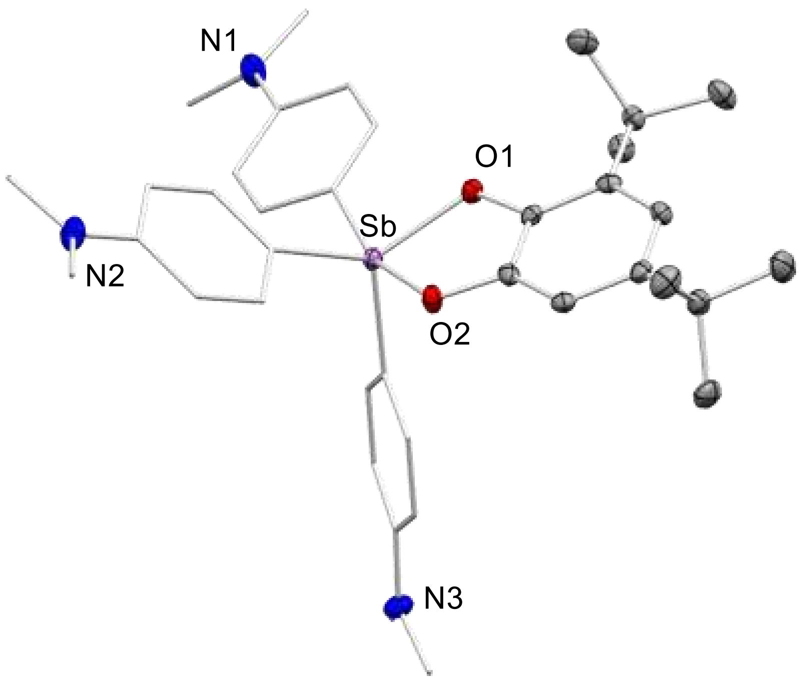

Figure S19. Molecular structure of **7** with hydrogen atoms omitted for clarity.

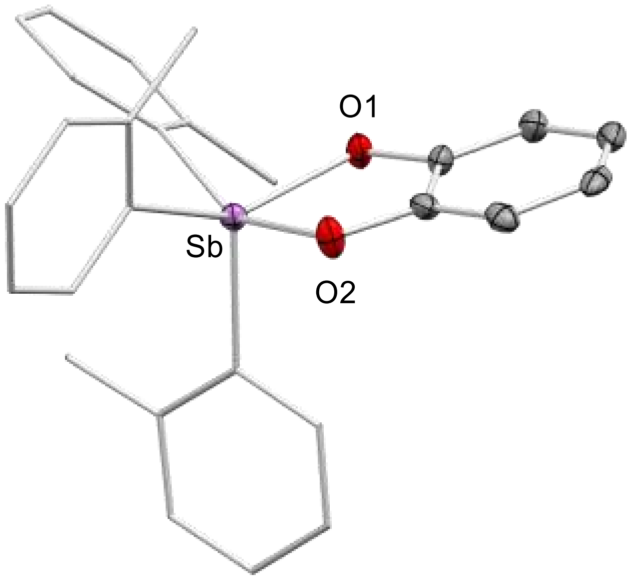

Figure S20. Molecular structure of **8** with hydrogen atoms omitted for clarity.

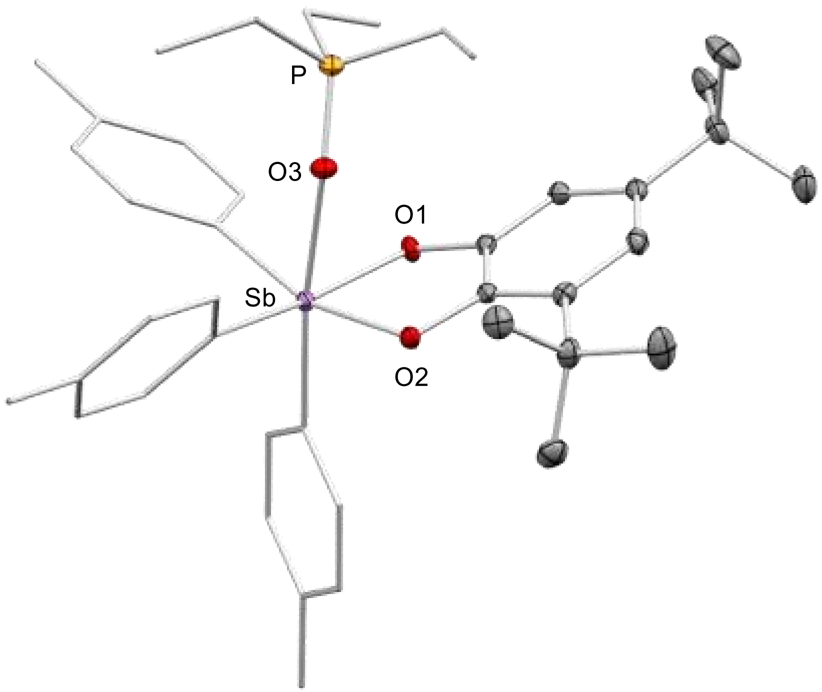

Figure S21. Solid state structure of **5-TEPO** adduct with hydrogen atoms omitted for clarity

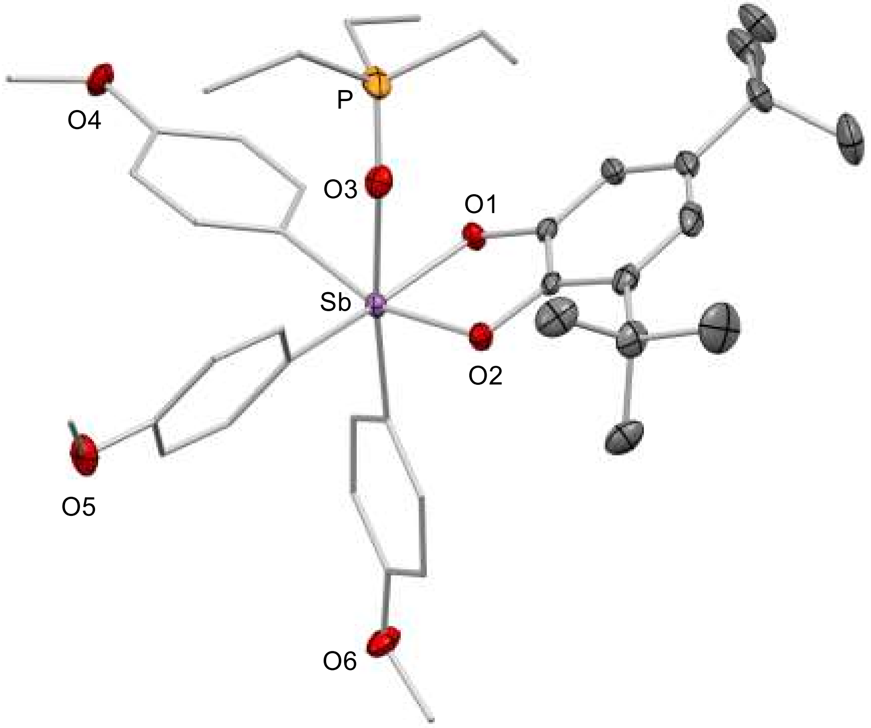

Figure S22. Solid state structures of **6-TEPO** adduct with hydrogen atoms omitted for clarity.

2 Complexation of Et_3PO by the Stiboranes.

The Et_3PO binding constants were determined by a reverse titration experiment in which a solution of Et_3PO was titrated with increasing amounts of the stiborane. These experiments were carried out by combining an Et_3PO stock solution (100 µL, 134 mM) with a stock solution of the stiborane as described in Table S1. In each case, the volume of the resulting sample was adjusted to 0.5 mL by the addition of pure CH_2Cl_2. A capillary containing DMSO-d_6 was inserted in each sample to provide an NMR lock signal. The equilibrium constant *K*a was obtained by fitting the ^{31}P NMR chemical shifts to a 1:1 binding isotherm (eqn 1).

Table S1. $^{31}P\{^1H\}$ NMR chemical shift collected during the titration experiments.

Stiborane/stock solution conc.		Volume added					
		0 µL	50 µL	100 µL	200 µL	300 µL	400 µL
	**1** / 137 mM	50.17	54.78	57.97	59.90	60.38	60.63
	**2** / 137 mM	50.17	53.18	55.17	57.40	58.72	58.95
	**3** / 152 mM	50.11	53.29	55.13	57.43	58.32	58.75
	**4**/ 273 mM	50.17	50.57	50.83	51.29	51.76	52.17
	**5**/ 137 mM	50.17	51.35	52.19	53.50	54.48	55.27
	**6**/ 137 mM	50.11	50.96	51.66	52.72	53.77	54.48
	**9**/ 70 mM	50.42	53.98	57.08	62.43	62.75	62.78

$$\Delta\delta = \frac{\Delta\delta_{max}}{2 \times c_{\mathrm{P}}} \times \left\{ \left(c_{\mathrm{P}} + c_{\mathrm{Sb}} + \frac{1}{Ka} \right) - \sqrt{\left(c_{\mathrm{P}} + c_{\mathrm{Sb}} + \frac{1}{Ka} \right)^2 - 4 \times c_{\mathrm{P}} \times c_{\mathrm{Sb}}} \right\} \quad (1)$$

with:

c_{P} = concentration of the Et_3PO in the NMR tube

c_{Sb} = concentration of the testing stiborane in the NMR tube

K_a = binding constant

Due to the high binding affinity between **9** and Et_3PO, the NMR titration curve saturated rapidly even at lower stiborane concentrations. While the data could not be fit accurately to extract a precise K_a value, the early saturation of the Δ^{31}P chemical shifts confirmed a much higher binding affinity ($K_a > 1000$ M^{-1}) than other stiboranes studied in this paper.

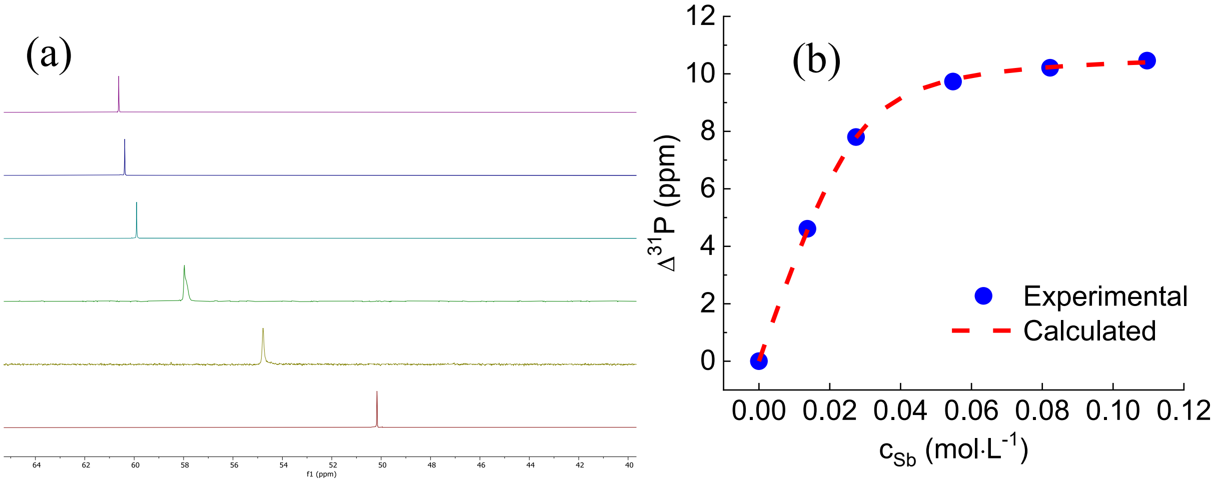

Figure S23. (a) Changes in the $^{31}P\{^{1}H\}$ NMR spectra of **TEPO** (2.70×10^{-2} M) observed upon incremental addition of **1**. (b) The experimental and the calculated 1:1 binding isotherm (Equation 1) for **1** and **TEPO** based on the $^{31}P\{^{1}H\}$ NMR chemical shifts. The data was fitted to yield $K_a = 330 \pm 18\ M^{-1}$, $R^2 = 0.99985$.

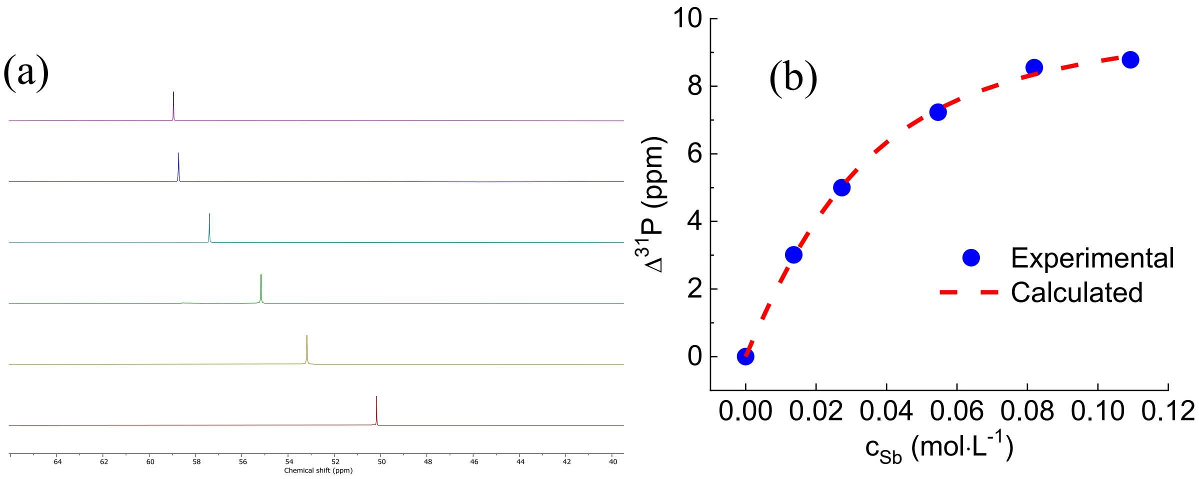

Figure S24. (a) Changes in the $^{31}P\{^{1}H\}$ NMR spectra of **TEPO** (2.70×10^{-2} M) observed upon incremental addition of **2**. (b) The experimental and the calculated 1:1 binding isotherm (Equation 1) for **2** and **TEPO** based on the $^{31}P\{^{1}H\}$ NMR chemical shifts. The data was fitted to yield $K_a = 64.6 \pm 7.5\ M^{-1}$, $R^2 = 0.99861$.

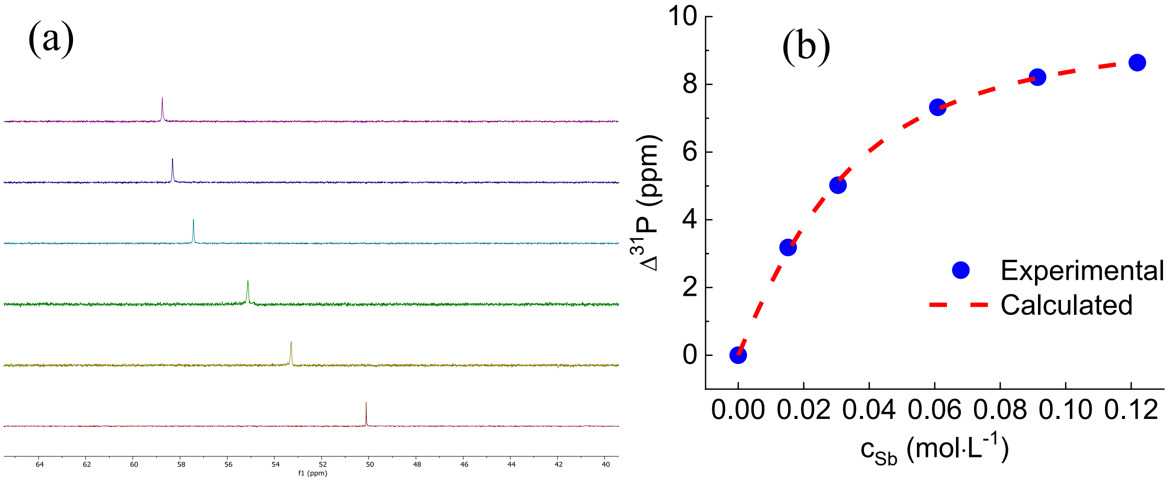

Figure S25. (a) Changes in the $^{31}P\{^1H\}$ NMR spectra of **TEPO** (2.70×10^{-2} M) observed upon incremental addition of **3**. (b) The experimental and the calculated 1:1 binding isotherm (Equation 1) for **3** and **TEPO** based on the $^{31}P\{^1H\}$ NMR chemical shifts. The data was fitted to yield K_a = 62.6 ± 4.8 M^{-1}, R^2 = 0.9993.

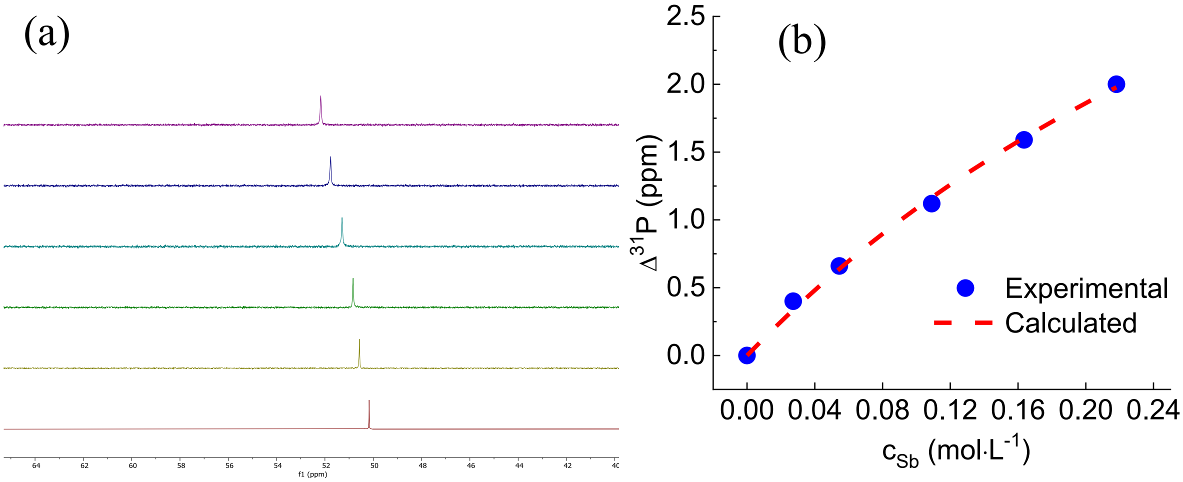

Figure S26. (a) Changes in the $^{31}P\{^1H\}$ NMR spectra of **TEPO** (2.70×10^{-2} M) observed upon incremental addition of **4**. (b) The experimental and the calculated 1:1 binding isotherm (Equation 1) for **4** and **TEPO** based on the $^{31}P\{^1H\}$ NMR chemical shifts. The data was fitted to yield K_a = 2.13 ± 0.54 M^{-1}, R^2 = 0.99677.

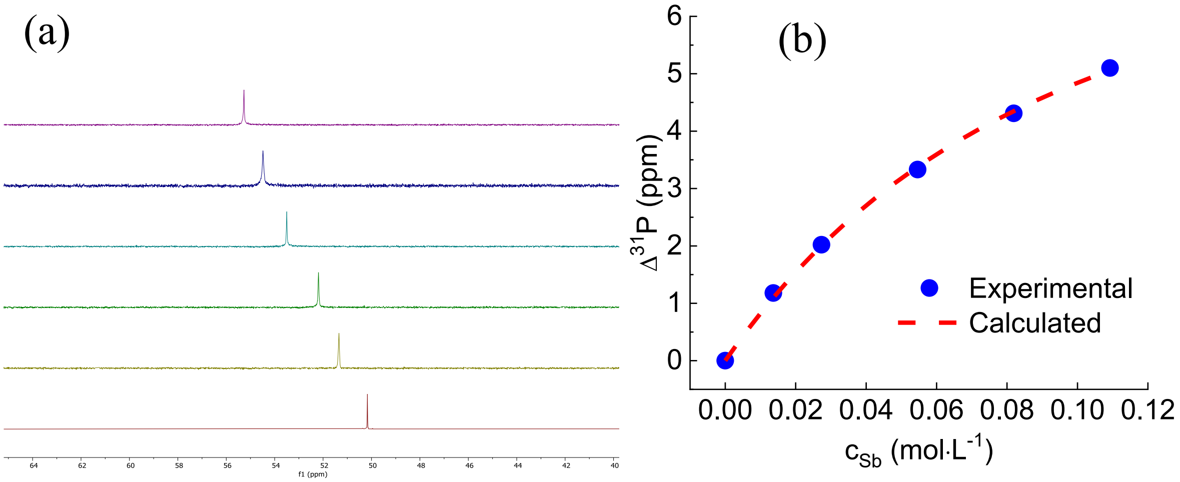

Figure S27. (a) Changes in the $^{31}P\{^{1}H\}$ NMR spectra of **TEPO** (2.70×10^{-2} M) observed upon incremental addition of **5**. (b) The experimental and the calculated 1:1 binding isotherm (Equation 1) for **5** and **TEPO** based on the $^{31}P\{^{1}H\}$ NMR chemical shifts. The data was fitted to yield K_a = 13.1 ± 1.3 M^{-1}, R^2 = 0.99917.

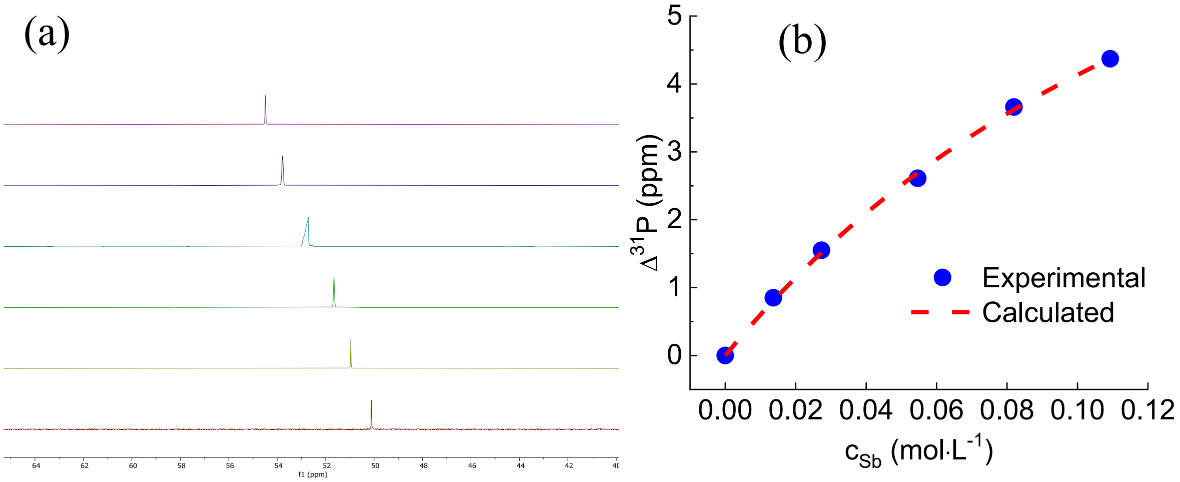

Figure S28. (a) Changes in the $^{31}P\{^{1}H\}$ NMR spectra of **TEPO** (2.70×10^{-2} M) observed upon incremental addition of **6**. (b) The experimental and the calculated 1:1 binding isotherm (Equation 1) for **6** and **TEPO** based on the $^{31}P\{^{1}H\}$ NMR chemical shifts. The data was fitted to yield K_a = 6.99 ± 0.92 M^{-1}, R^2 = 0.99901.

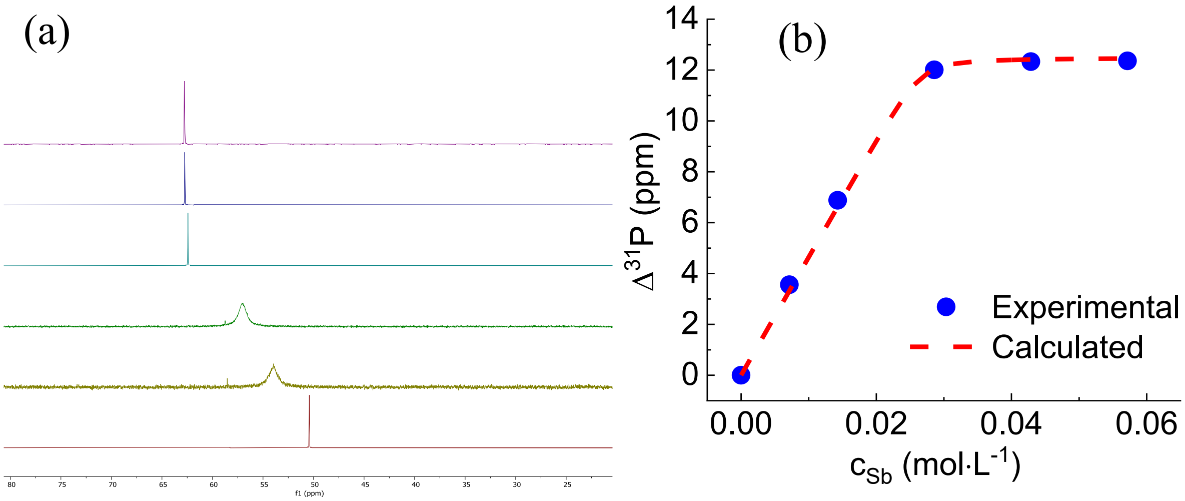

Figure S29. (a) Changes in the $^{31}P\{^1H\}$ NMR spectra of **TEPO** (2.70×10^{-2} M) observed upon incremental addition of **9**. (b) The experimental and the calculated 1:1 binding isotherm (Equation 1) for **9** and **TEPO** based on the $^{31}P\{^1H\}$ NMR chemical shifts. The data was fitted to yield K_a = 10059 ± 6213 M^{-1}, $R^2 = 0.99948$.

3 Stiborane-Catalyzed Copolymerization Study

3.1 CO_2/Epoxide Copolymerization Measured by FTIR Spectroscopy in a Parr Reactor

The copolymerization of CO_2 and epoxide was measured using a ReactIR system (Mettler-Toledo LLC, Columbus, OH) equipped with an Attenuated Total Reflection (ATR) probe possessing a diamond window. The C-22 Hastelloy probe was inserted into the base of a 600 mL Parr reactor and connected to the FTIR spectrometer via a silver halide (AgX) fiber optic cable, allowing for in situ measurements of CO_2/epoxide polymerization. The FTIR spectra were collected using a resolution of 8 and a wavenumber spacing of 4 cm^{-1}, covering a spectral range of 650 to 3500 cm^{-1}. The spectra of the cyclohexene oxide (CHO) before and after polymerization can be seen in Figure S30, where peaks related to the epoxide (COC stretch) and the polycarbonate (C=O stretch) are identified.[7, 8]

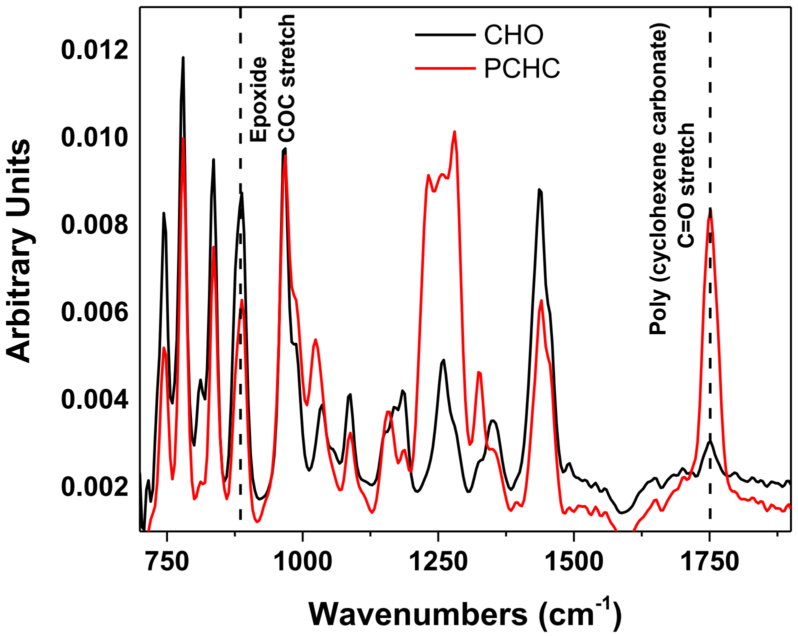

Figure S30. FTIR spectra of CHO and poly (cyclohexene carbonate) (PCHC) with peak assignments for the epoxide and polycarbonate.

FTIR spectra were imported into MATLAB (The MathWorks, Inc., Natick, Massachusetts) and baseline corrected using an asymmetric reweighted penalized least squares algorithm over the spectral range of 652 to 1900 cm^{-1}.[9] Following baseline correction, the spectral regions due to the epoxide (860 – 916 cm^{-1}) and the poly (cyclohexene carbonate) (1668 – 1900 cm^{-1}) were extracted, combined, and normalized to unit area. Unit area normalized regions were resolved into pure spectral components and concentration profiles using window factor analysis (WFA).[10-12] The number of components selected to be modeled was 2, which represents the CHO and poly

(cyclohexene carbonate) (PCHC). Uncalibrated concentration profiles were estimated using the window factor analysis (WFA) equation below.

$$\beta_{nn} s_n^0 c_n' = \left(I - S^0 S^{0'}\right) D = X_n \qquad (2)$$

Equation (2) was used to calculate the uncalibrated concentration profiles, X_n, for the CHO and PCHC components, which were utilized to approximate the spectral components using linear least squares as can be seen below.

$$S = DC'(CC')^{-1} \qquad (3)$$

Where *S* are the resolved spectral components for CHO and PCHC. *D* is the matrix of unit area normalized FTIR spectra consisting of the epoxide and poly (cyclohexene carbonate) regions, and *C* are the uncalibrated concentration profiles calculated using window factor analysis. Spectral and concentration profiles derived from WFA were used as inputs to alternating least squares (ALS) algorithm. Alternating least squares (ALS) was used to apply non-negativity constraints to the spectral and concentration profiles, which was achieved by minimization of the equation given below.

$$min_{W,H}\, f(W,H) = \frac{1}{2}\|D - WH\|_F^2 \;\; s.t. \;\; W, H \geq 0 \qquad (4)$$

W and *H* are the estimated spectral and concentration profiles with initial inputs derived from WFA. Optimization of *W* and *H* in Equation (4) was performed iteratively using the given equations below.

$$H = (W'W)^{-1}W'D \qquad (5)$$

$$W' = (HH')^{-1}HD' \qquad (6)$$

Analysis of the FTIR spectra using ALS produced spectral and concentration profiles for CHO and PCHC, which evolved with time as CO_2 and CHO copolymerized to form PCHC. Spectral profiles were normalized to unit area and used in a linear least squares computation as in Equation (3) to yield concentration profiles as can be seen in Figure S31 for catalyst **5**.

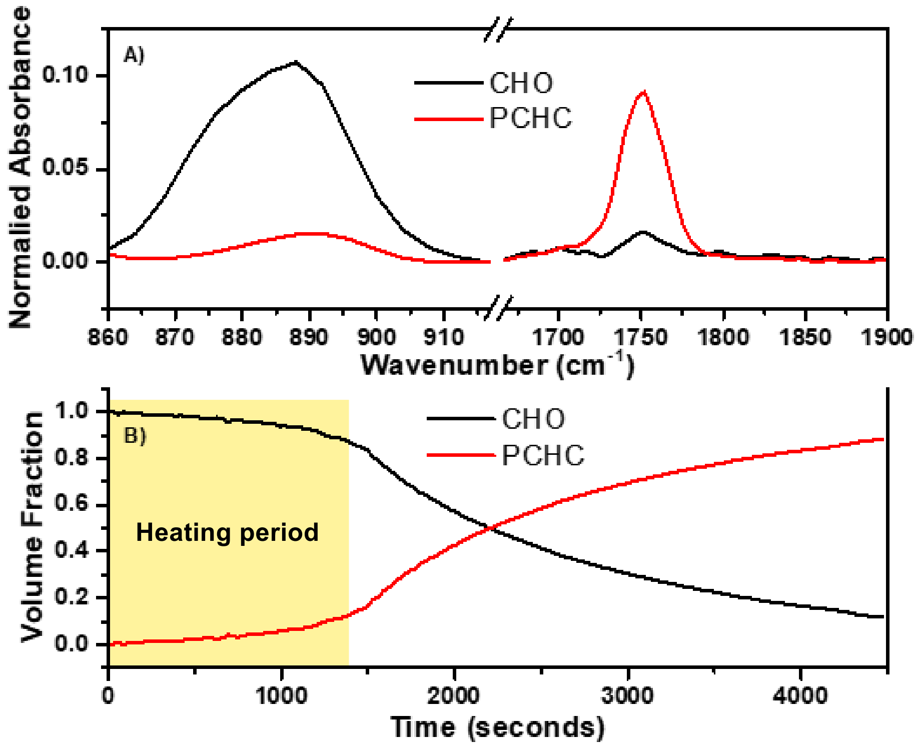

Figure S31. Resolved A) spectra and B) concentration profiles for CHO and PCHC obtained for catalyst **5** using ALS.

3.2 Initial Rate Method of Analyzing the Polymerization Rate

The concentration profiles resolved using ALS are a measurement for the volume fraction of CHO and PCHC present during the copolymerization reaction. In addition to the CHO and PCHC volume fractions, a priori knowledge of monomer volume and catalyst mass were utilized to determine reaction rates and catalyst kinetics for the antimony-based catalyst systems. Furthermore, corrections for changes in volume expansion due to the copolymerization of CHO with CO_2 were applied. Conversion of CHO was measured as a function of time as can be seen in Figure S32, where a linear fit was applied to the initial part of the curve. The slope of the fitted line is equivalent to the initial rate as can be seen in Equation 6 below. Initial reaction rates were used to determine pseudo-first-order rate constants as a function of catalyst and CO_2 concentration in Figure 6 of the main text.

$$Initial\ Rate = \frac{d[CHO]}{dt} = -k_{obs}[CHO] = slope \qquad (7)$$

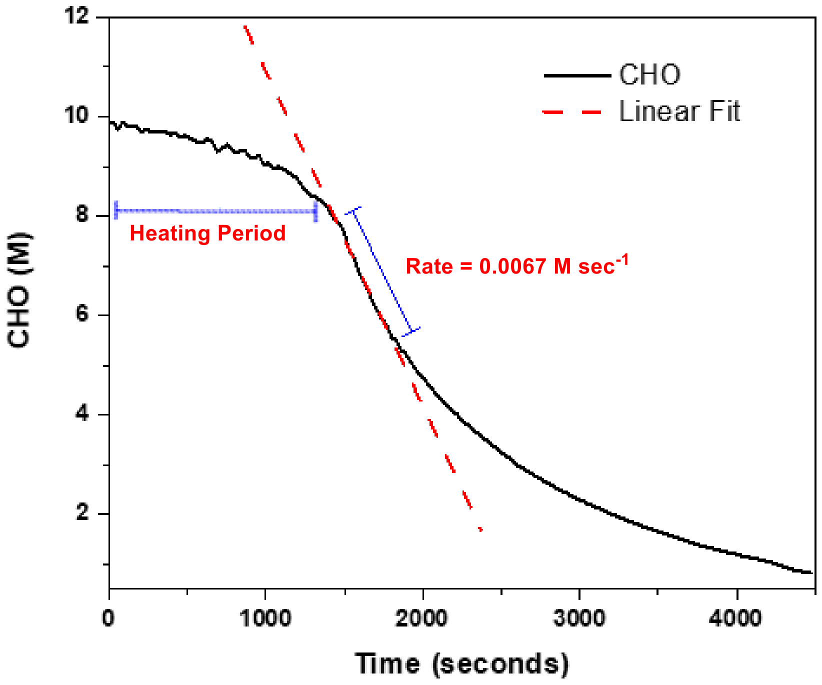

Figure S32. Initial rate analysis of CHO copolymerization reaction with CO_2 (750 psi) using catalyst **5** measured by FTIR spectroscopy at 80 °C.

3.3 First-Order Direct Fitting Method of Analyzing the Polymerization Rate

The ln(A_o/A_t) was plotted for specified catalyst concentrations according to Equation 8 and 9 below:

$$[A]_t = [A]_0 e^{-k_{obs}t} \qquad (8)$$

$$\ln\left(\frac{A_0}{A_t}\right) = k_{obs}t \qquad (9)$$

Three exemplary experiments analyzed via linear regression fit according to Equation 8 are shown in Figure S33.

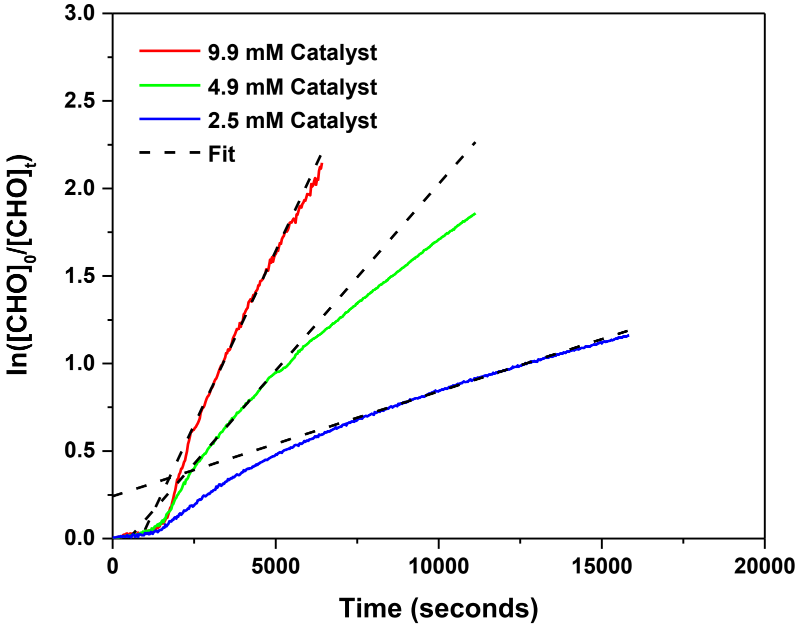

Figure S33. Plots of ln($[CHO]_0$/$[CHO]_t$) vs time obtained for the variable catalyst concentration kinetic experiments wherein the first-order rate constants were fitted according to Equation 9.

3.4 CO_2 Polymerization Experiments (Small Scale Screening)

The polymerization experiments of CO_2 and CHO were performed in a stainless-steel vessel. A desired amount of the catalyst was first dissolved in 100 μL of CHO. The vessel was then pressurized with 3.10 MPa CO_2, isolated, and heated to the desired temperatures for 12 hours. The reaction was then brought back to ambient temperature and depressurized. The reaction mixture was then dissolved in 1 mL $CDCl_3$ containing 1,3-bis(trimethylsilyl)benzene (5 mM) as an internal standard for quantification. The turnover number (TON), corresponding to numbers of epoxides converted into polymers per catalyst, was determined by ^1H NMR spectroscopy. The polymers were isolated by drying in a vacuum oven. The molecular weights were determined by GPC methods using dn/dc value of 0.089 mL/g.

Size Exclusion Chromatography. SEC data were collected using Tosoh BioScience HLC-8320 GPC equipped with an internal differential refractive index (dRI) detector, an 724 internal UV absorbance detector UV-8320 (254 nm absorbance detector), a Wyatt Technology miniDawn TREOS light scattering detector with three angles (45°, 90°, and 135°). For the analysis of poly cyclohexene carbonate, the molar mass was determined using the light scattering detector with a dn/dc value of 0.089 mL/g. The mobile phase was THF with a 1 mL/min flow rate. The GPC pump oven and column oven temperature is 40 °C. The Wyatt Technology's Astra 6.1.7.17 Gel Permeation Chromatography Software was used for data analysis.

3.5 Estimation of *K*eq of Based on Our Mechanism Model.

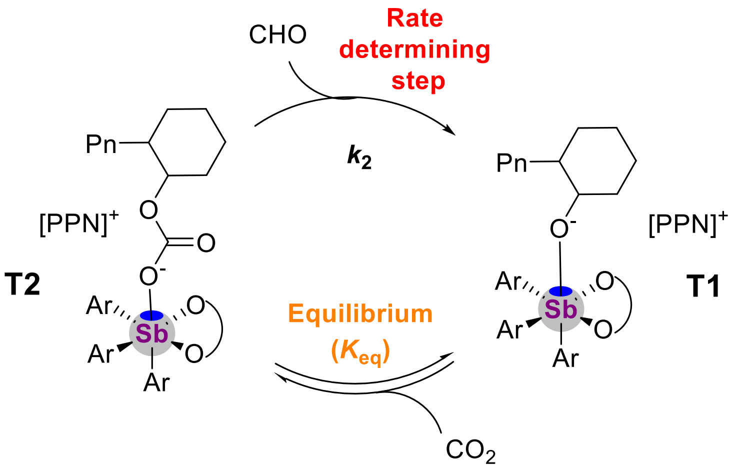

Figure S34. Simplified kinetic scheme of CO_2 epoxide alternating copolymerization.

Steady-state approximations were used to assess the kinetics depicted in Figure S34. The overall catalyst concentration was equal to the sum of the concentrations of the alkoxide (T1) and carbonate intermediates (T2). The equilibrium constant *K*eq was obtained by fitting the kinetic data to the following Equation 12. The pressure of CO_2 is brought into the equations to reflect the concentration of carbon dioxide, which is under the assumption that Harry's law is applicable in this system. Therefore, the unit of the calculated equilibrium constant *K*eq is MPa^{-1}.

$$[\mathrm{T1}] + [\mathrm{T2}] = [\mathrm{Cat.}] \quad (10)$$

$$K\mathrm{eq} = \frac{[\mathrm{T2}]}{[\mathrm{T1}] \cdot [\mathrm{CO2}]} = \frac{[\mathrm{T2}]}{([\mathrm{Cat.}] - [\mathrm{T2}]) \cdot [\mathrm{CO2}]} \quad (11)$$

$$\frac{d[\mathrm{PCHC}]}{dt} = k_{initial} \cdot [\mathrm{CHO}] = k_2 \cdot [\mathrm{T2}] \cdot [\mathrm{CHO}] = k_2 \cdot [\mathrm{CHO}] \left(\frac{K\mathrm{eq} \cdot [\mathrm{CO2}] \cdot [\mathrm{Cat.}]}{1 + K\mathrm{eq} \cdot [\mathrm{CO2}]} \right) \quad (12)$$

With:

[T1] = concentration of alkoxide species

[T2] = concentration of carbonate species

$[Cat.]$ = concentration of catalyst = 9.88×10^{-3} M

$[CO2]$ = concentration of carbon dioxide. (CO_2 pressures were used in data fitting)

k_2 = rate constant for the rate determined step.

K_{eq} = equilibrium constant for CO_2 insertion step.

Table S2. Experimental and calculated rate constant for compound **5** catalyzed CO_2/epoxide polymerization.

CO_2 Pressure (MPa)	$k_{initial}$ (10^{-4} s^{-1})	$k_{cal.}$ (10^{-4} s^{-1})^b
0.0000	0.00	0.00
0.6895	2.44	2.40
1.3790	4.09	3.79
2.7579	4.79	5.32
5.1711	6.81	6.57

^{a}Experimentally observed $k_{initial}$ from varying CO_2 pressure. ^{b}rate constant calculated based on eqn 4, with K_{eq} = 0.53 MPa^{-1}, and k_2 = 0.090623793 M^{-1} s^{-1}.

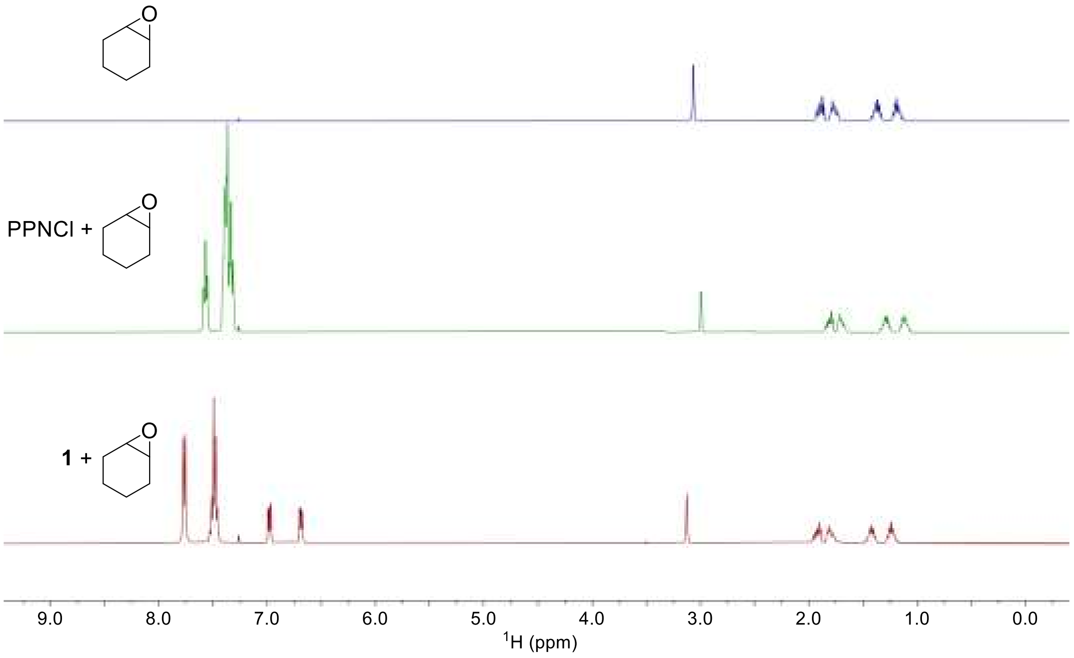

Figure S35. ^{1}H NMR (400 MHz, $CDCl_3$) of cyclohexene oxide (top), a 1:1 mixture of PPNCl and cyclohexene oxide (middle), and a 1:1 mixture of complex **1** and cyclohexene oxide (bottom), after being kept at 60 °C for 30 minutes.

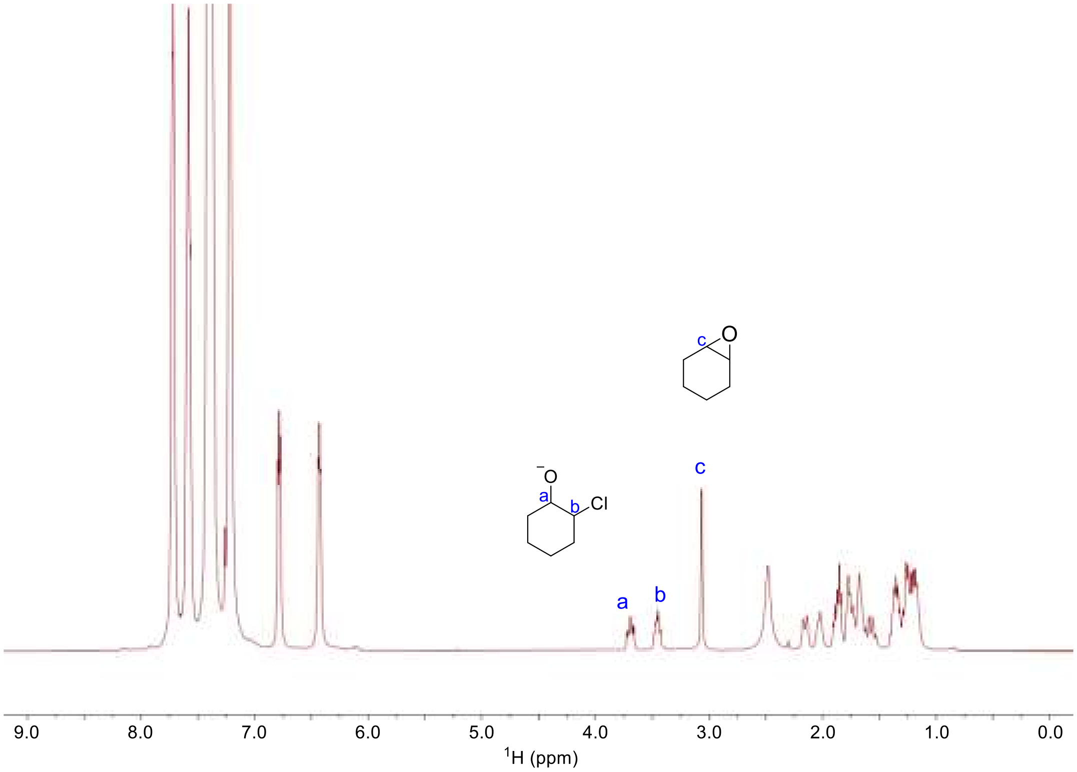

Figure S36. ^{1}H NMR (400 MHz, $CDCl_3$) of a 1:1:1 mixture of cyclohexene oxide, PPNCl, and complex **1** after being kept at 60 °C for 30 minutes. The chemical shifts of the 2-chlorocyclohexanol product match those reported in the literature.[13]

3.6 Polymer Characterization

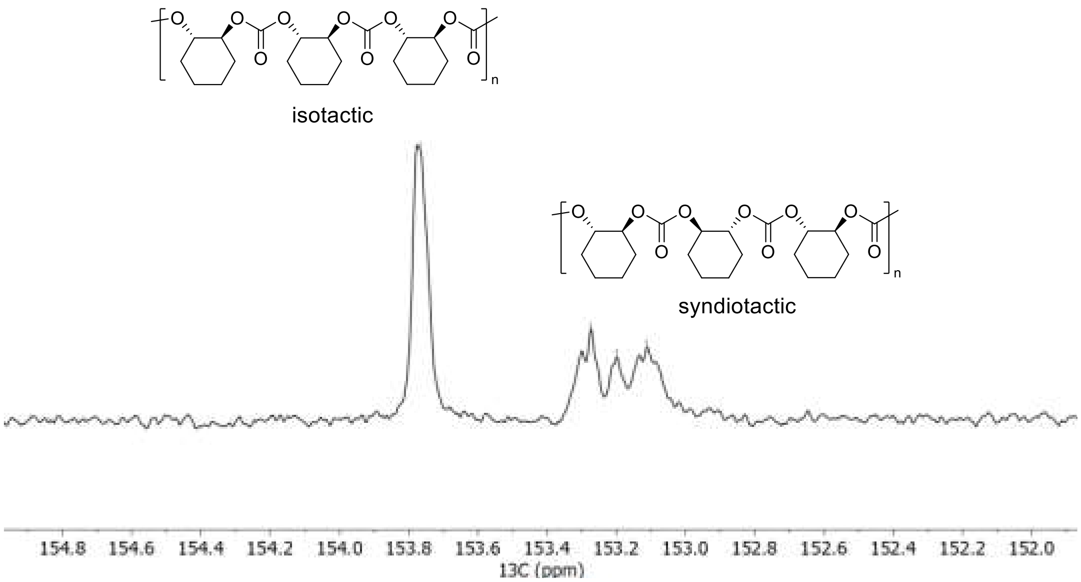

Figure S37. ^{13}C NMR spectrum of the resulting PCHC recorded at 400MHz in $CDCl_3$ (region 152−155 ppm).

The polymer sample for MALDI-TOF mass spectrometry was prepared using a higher catalyst loading to limit the polymer molecular weight. This approach was designed to obtain a better resolution of the mass spectra, allowing for accurate identification of the polymer end groups. The CO_2/epoxide copolymerization was carried out using compound **1** as the catalyst under the following conditions: [CHO]/[catalyst]/[PPNCl] = 100/1/1, 1 mL neat CHO, 0.122 MPa CO_2, at 80 °C for 12 h.

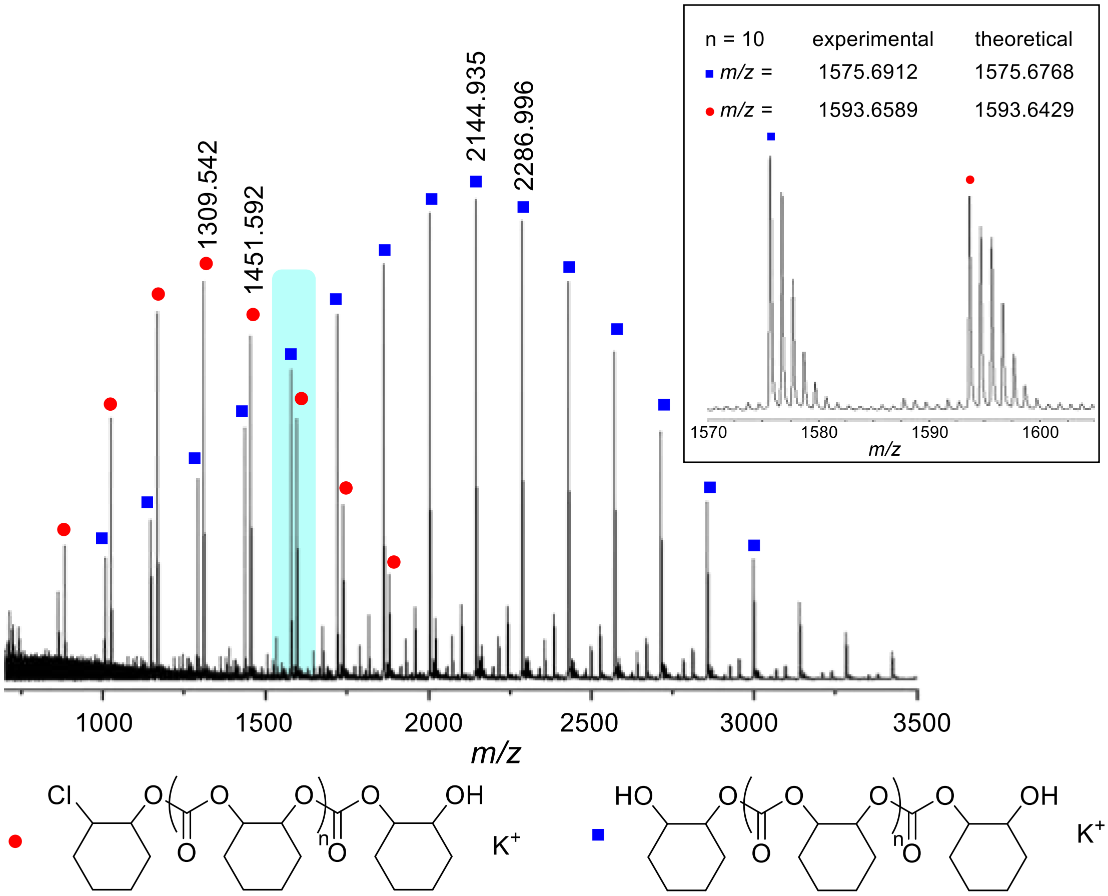

Figure S38. MALDI-TOF spectrum collected for the prepared sample (Inset: zoom from *m/z* = 1570 to *m/z* = 1605).

To determine whether water present in the polymerization system originates from the CO_2 feed and to assess its potential role as a chain transfer agent responsible for the experimentally observed M_n values being lower than the theoretical values, three copolymerization experiments were performed using compound **1** as the catalyst under the following conditions: [CHO]/[catalyst]/[PPNCl] = 100/1/1, 1 mL neat CHO, CO_2 pressure of 0.122 MPa, 80 °C, 16 h. In the control experiment, commercially supplied CO_2 was passed through a column packed with P_2O_5 prior to use. In the second experiment, CO_2 was further dried by sequential passage through a P_2O_5 column followed by bubbling through neat Al(^{i}Bu)$_3$. In the third experiment, 10 μL of deionized water was added directly to the reaction mixture after pressurizing with CO_2 (dried through P_2O_5 as in the control). Upon completion, the reaction mixtures were depressurized, and the resulting polymers were dried under vacuum before characterization by MALDI-TOF mass spectrometry.

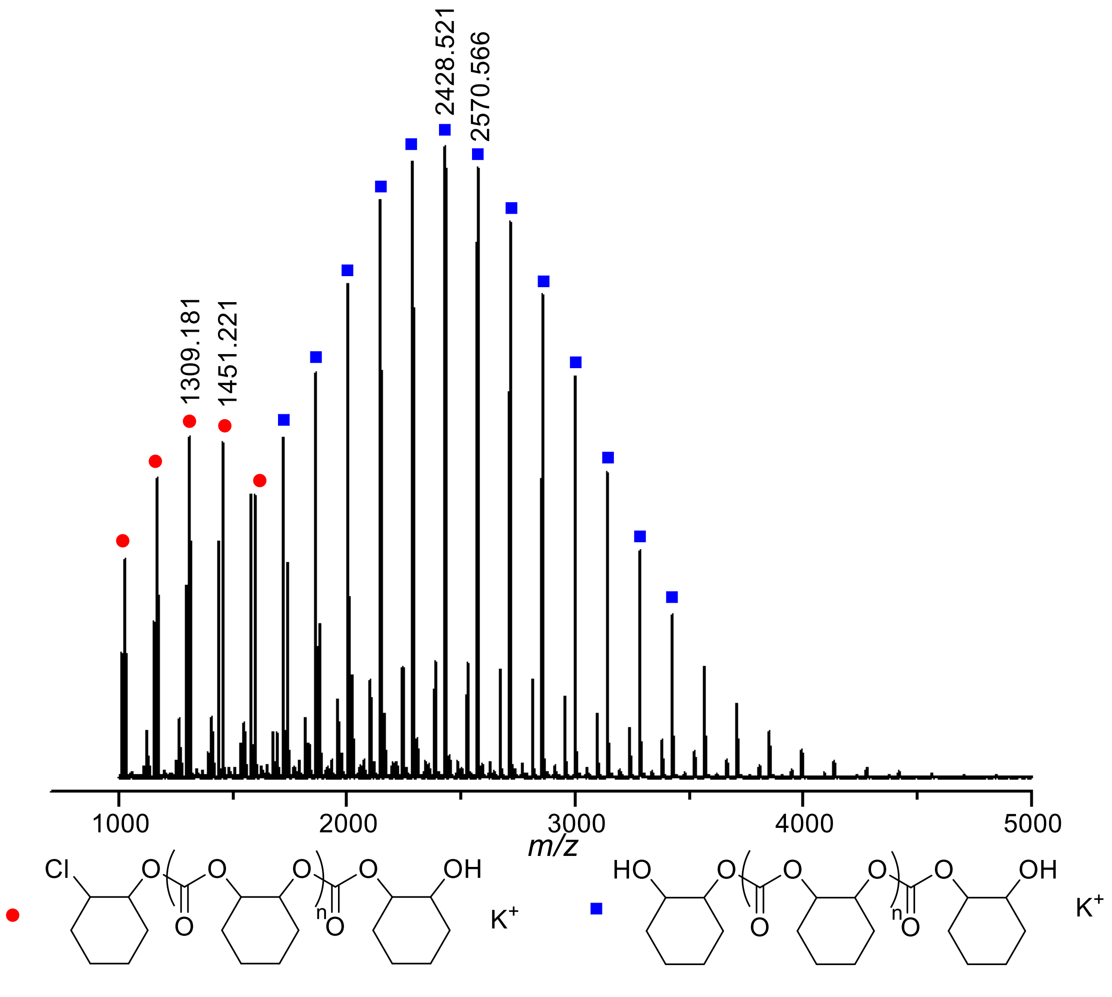

Figure S39. MALDI-TOF spectrum collected for the prepared sample with P_2O_5-dried CO_2.

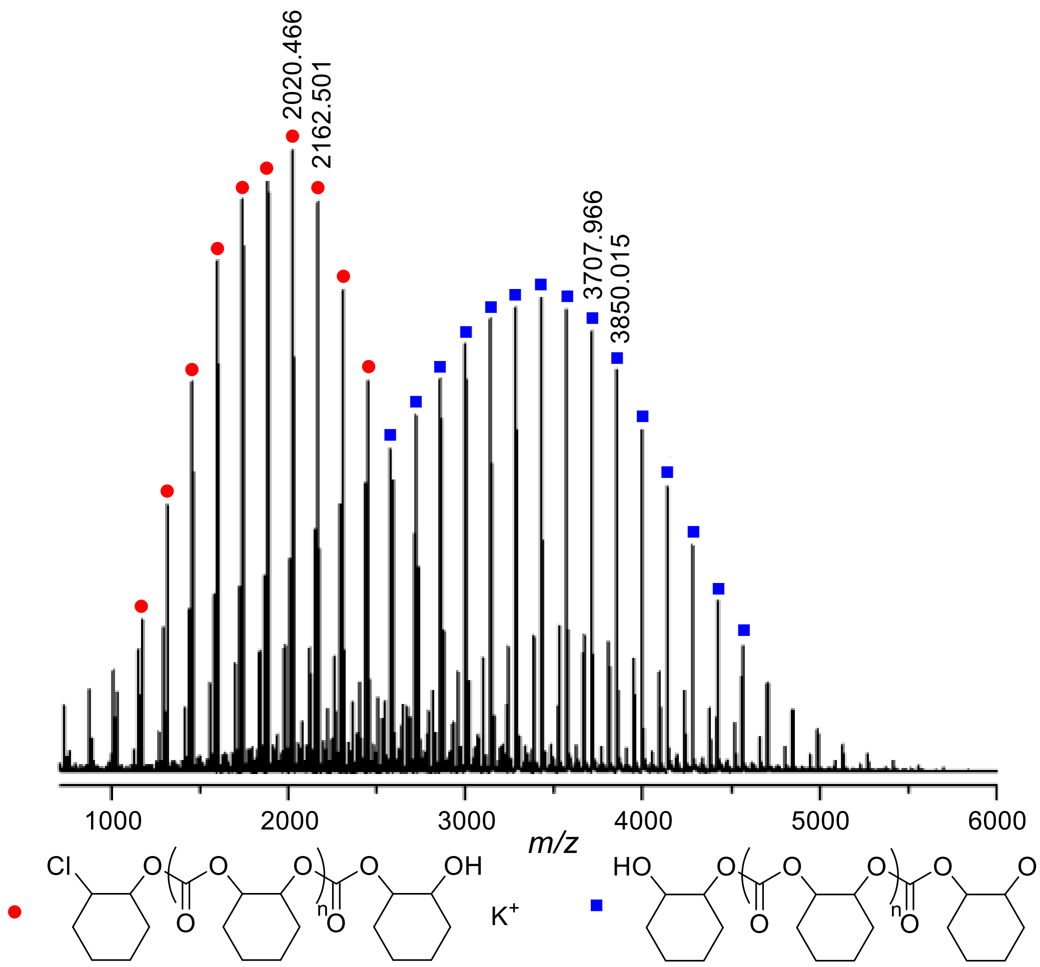

Figure S40. MALDI-TOF spectrum collected for the prepared sample with P_2O_5 and $Al(^iBu)_3$ dried CO_2.

The comparison experiments were carried out using each catalyst under identical conditions to allow for a direct performance evaluation. The copolymerization of CO_2 and CHO was conducted with a [CHO]/[catalyst]/[PPNCl] ratio of 100/1/1 in 1 mL neat CHO, 0.122 MPa CO_2 at 80 °C for 3 h. A small aliquot of the reaction mixture was removed for ^{1}H NMR analysis to determine the CHO conversion. The resulting polymers were dried under vacuum and subsequently analyzed by MALDI-TOF mass spectrometry. Average molecular weights (M_n, M_w) were calculated from the following equation[14] 13, 14:

$$M_n = \frac{\Sigma(N_i M_i)}{\Sigma N_i} \quad (13)$$

$$M_w = \frac{\Sigma(N_i M_i^2)}{\Sigma(N_i M_i)} \quad (14)$$

Where N_i and M_i represent signal intensity in peak area and mass for the oligomer containing *i* monomers, respectively.

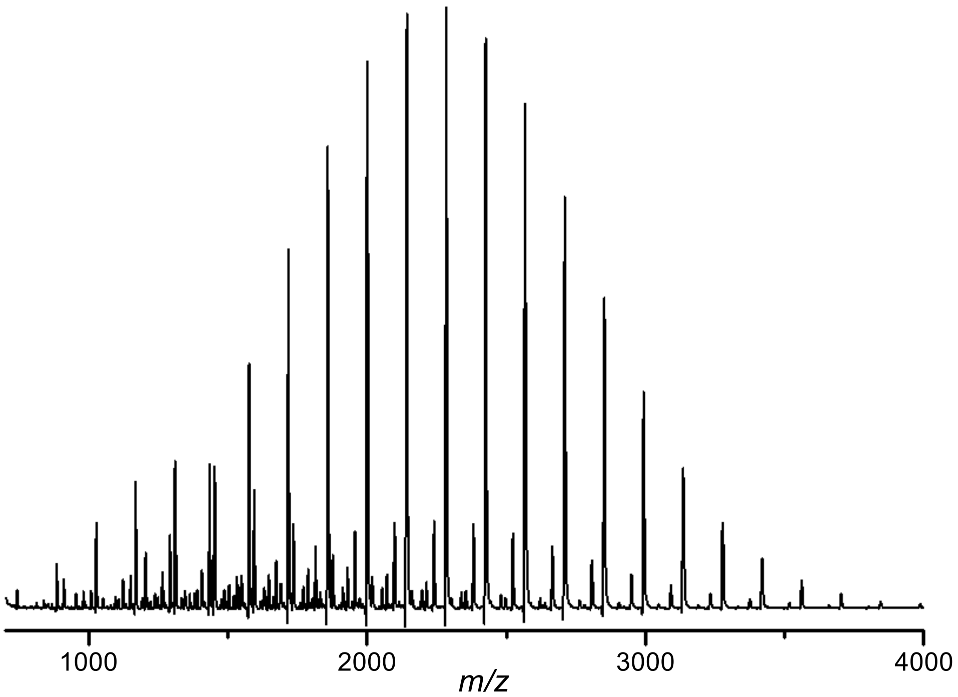

Figure S41. MALDI-TOF spectrum collected for the polymer sample prepared with compound **5**.

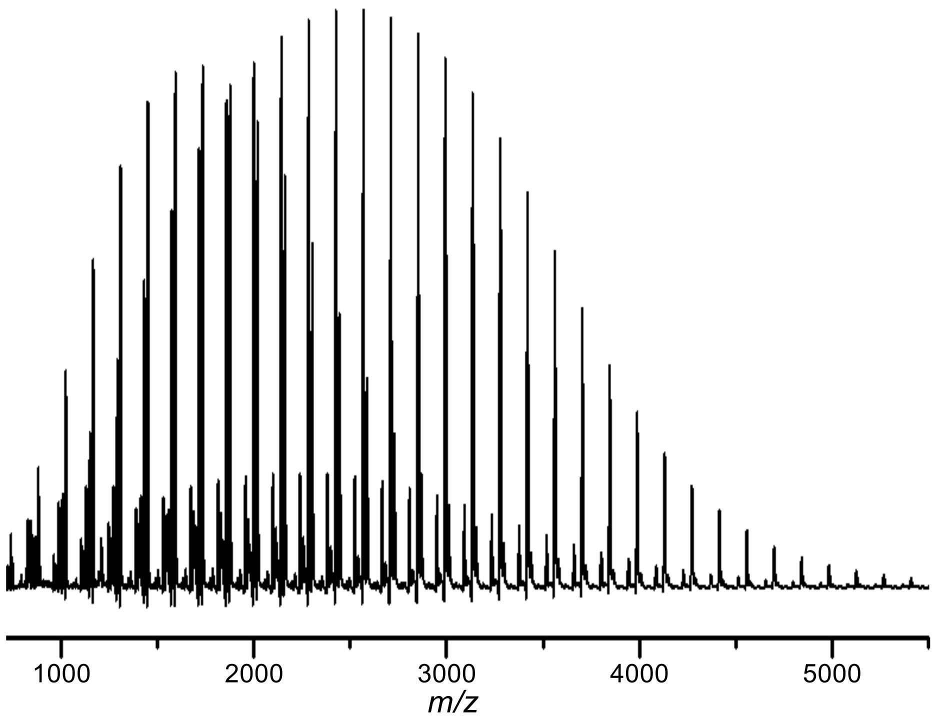

Figure S42. MALDI-TOF spectrum collected for the polymer sample prepared with Cataylst **12**.

To determine whether water present in the polymerization system originates from the CHO, copolymerization experiments were performed using compound **5** as the catalyst under the following conditions: [CHO]/[catalyst]/[PPNCl] = 1000/1/1, 40 mL neat CHO, CO_2 pressure of 2.76 MPa, 80 °C, 12 h. In the control experiment, CHO was dried by distillation over CaH_2. In the second experiment, CHO was further purified by distillation first over CaH_2 and subsequently over *t*-butyllithium.

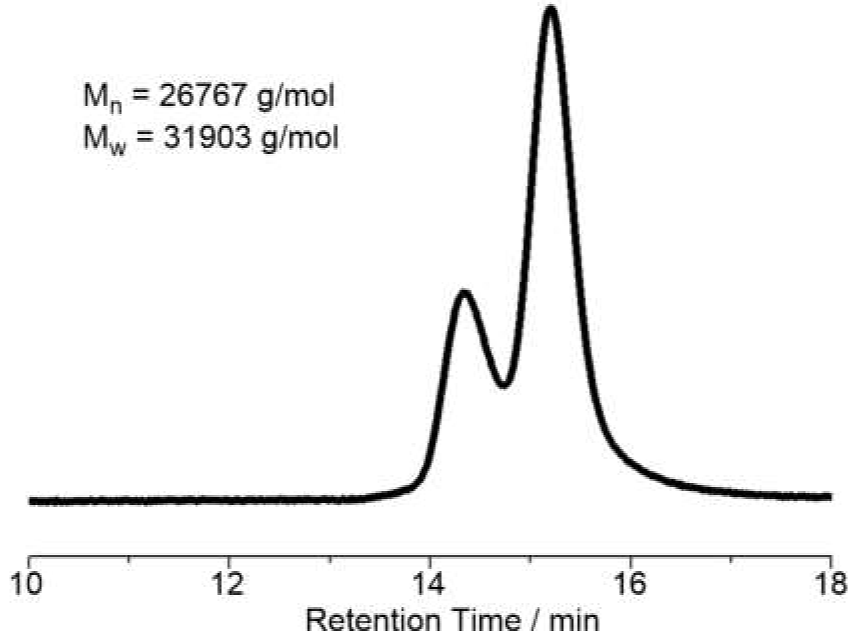

Figure S43. GPC trace of the polymer sample prepared with CaH_2 dried CHO.

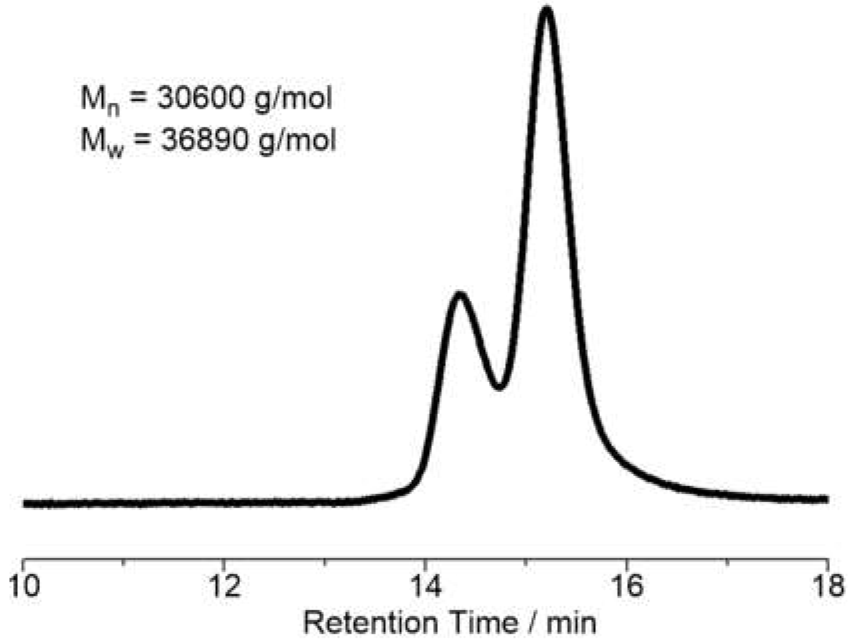

Figure S44. GPC trace of the polymer sample prepared with CaH_2 and ^{t}BuLi dried CHO.

4 Computation Studies

4.1 Methods

Density functional theory (DFT) structural optimizations were performed with the Gaussian 16 program.[15] In all cases, the crystal structure geometries were optimized using the B3LYP functional[16, 17] and the following mixed basis sets: aug-cc-pVTZ-PP[17-19] for Sb, 6-31G(d')[20, 21] for F, and 6-31G[22, 23] for C, O, N, and H. For all optimized structures, frequency calculations were performed in order to confirm the absence of imaginary frequencies. Single point calculations carried out at the optimized geometry with the B3LYP functional and the following mixed basis sets: aug-cc-pVTZ-pp for Sb and 6-311+g(2d,p) for C, H, O, and N. The enthalpies used to derive the FIA were obtained through single-point calculations performed at the optimized geometry using the B3LYP functional and the following mixed basis sets: aug-cc-pVTZ-pp for Sb and 6-311+g(2d,p) for C, H, O, N, and F. The enthalpy correction term was obtained from the above-mentioned frequency calculations.

4.2 Percent Volume Buried (%V_{Bur}).

The percent volume buried (%V_{Bur}) of these stiboranes in their fluoride adducts serves as the parameter that characterizes the accessibility of the Lewis acids. This concept was initially demonstrated by Radius in 2023.[24] The stiborane-fluoride adducts were first optimized with the fluoride trans to one of the aryl substituents on the antimony center, using above-mentioned DFT methods. The optimized geometries were then subjected to SambVca 2.1[25] with the following parameters: the fluoride atom was selected as the center of the sphere, the antimony atom was selected to define the *z*-axis, and the two oxygen atoms on the catechol moiety were selected to define the *xz*-plane. The fluoride atoms were then deleted from the geometries. Bondi radii was scaled by 1.17, sphere radius was set at 3.5 Å, mesh spacing for numerical integration was set at 0.10, and hydrogen atoms were included in the calculation.

4.3 Electrostatic Potential Maps and $V_{S,max}$ Calculations.

Electrostatic potential (ESP) maps were generated based on the gas-phase optimized geometries of the stiborane-fluoride and determined at an isodensity value of 0.0015 electrons/Bohr^3. In all cases, the bound fluorides were removed, and the resulting structures were subjected to a single-point calculation. ESP maps were generated and analyzed using Multiwfn[26] and visualized in VMD software.[27] Multiwfn was also used to identify areas of maximum electrostatic potential ($V_{S,max}$).

4.4 Optimized Structures and Coordinates of Stiborane Compounds

Table S3. XYZ coordinates of the optimized geometry of **1**.

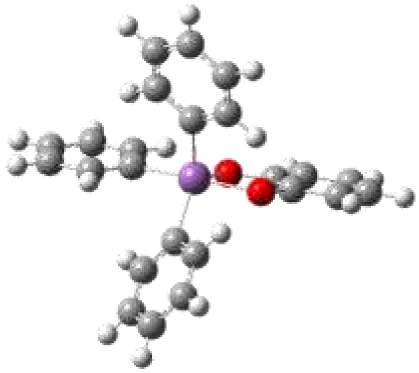

Sb	0.03553102	-0.01820514	0.03109395	C	-0.69101079	2.01826843	-2.1891873
O	-1.06618339	0.09648238	1.73195089	C	1.35085338	2.59082797	-1.00546749
O	-1.89215606	-0.29177496	-0.73451686	C	1.4943834	3.72694048	-1.80785318
C	0.56739485	-1.86315755	-0.92798232	C	-0.53783884	3.15364417	-2.98953996
C	0.26046816	1.72998681	-1.19782414	H	1.82291256	-2.55025966	0.69654569
C	-2.41607959	0.00226578	1.53860194	H	3.14764188	-0.2670809	-0.32179626
C	1.78681536	0.1982928	1.30301471	H	0.72334818	0.69843589	3.11226067
C	-2.8526728	-0.20618604	0.21228807	H	1.45603998	-5.23586815	-2.64350115
C	1.41014523	-2.77810277	-0.28342589	H	2.78004427	1.005741	4.46589224
C	3.05475338	0.01666807	0.72627333	H	-0.64912701	-1.48302133	-2.67736976
C	1.69809528	0.55907496	2.65644144	H	-2.9667874	0.26011244	3.5962651
C	1.20734436	-4.29187684	-2.16263505	H	5.01938273	0.68051875	3.429639
C	2.86048438	0.72939	3.41611389	H	5.1907944	0.04501506	1.02677385
C	0.03331727	-2.17226197	-2.18852425	H	2.37223865	-4.70462048	-0.39087883
C	-3.32966345	0.10021976	2.58341686	H	-0.04533923	-3.61333961	-3.78727584
C	4.11787419	0.54673651	2.83480624	H	0.66631914	4.89184312	-3.42442496
C	4.21524232	0.19026825	1.48719308	H	-5.41810387	0.0612998	3.11998568
C	1.72435421	-3.99385357	-0.90008462	H	-4.54485056	-0.48386887	-1.07950848
C	0.36418984	-3.38052582	-2.80629517	H	-6.20142358	-0.31029384	0.78620757
C	0.55316438	4.00678943	-2.80137394	H	-1.5487168	1.36509709	-2.31540122
C	-4.69940973	-0.01355051	2.30673849	H	2.08565699	2.38563063	-0.2315079
C	-4.21766254	-0.31862712	-0.05529866	H	2.34059602	4.39299351	-1.6510022
C	-5.13760687	-0.2213991	0.99738991	H	-1.27760468	3.37347558	-3.75688313

Table S4. XYZ coordinates of the optimized geometry of **1-F**.

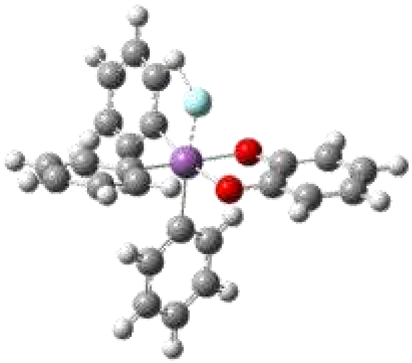

Sb	0.03022472	-0.01750548	-0.37425482
O	-1.57708285	-1.35164698	-0.38419113
O	-1.56458072	1.31725779	-0.52050246
C	-0.07161001	0.07873001	1.83129557
C	-0.79596324	1.10391306	2.46257559
C	0.46593464	-0.82479494	4.03453164
C	0.55409015	-0.88357688	2.63823172
C	-0.25347825	0.20358092	4.64823754
C	-5.15727627	0.67053164	-0.96633392
C	-2.75461901	-0.73282319	-0.5751819
C	1.42844532	1.65036665	-0.61001711
C	-0.88527199	1.16794168	3.85722347
C	3.20696412	-3.87171003	-0.68439618
C	3.68675081	-2.58332533	-0.43711136
C	2.13088004	2.17688997	0.4839919
C	1.41224219	-1.70574996	-0.48528262
C	0.94127717	-3.00437033	-0.73652094
C	-3.94843479	1.37580596	-0.85183485
C	3.02922647	3.23903058	0.31981136
C	2.79324196	-1.50964029	-0.34005617
C	1.83208305	-4.07824502	-0.83493742
C	-2.74838614	0.69095698	-0.651394
C	-5.16432522	-0.72203887	-0.88757517
C	-3.96299593	-1.42261436	-0.69458027
C	1.6432493	2.21451865	-1.87820742
C	2.53373572	3.27978129	-2.04355794
C	3.23117371	3.79404475	-0.94569502
H	-1.29636136	1.85082223	1.85044748
H	0.95817915	-1.58544695	4.64082348
H	1.11371558	-1.69501536	2.17600111
H	-0.32459761	0.25155654	5.73463659
H	-6.08712108	1.21839945	-1.11801625
H	-1.45384753	1.97100796	4.32636921
H	3.89970057	-4.70938339	-0.76189896
H	4.75678421	-2.40978717	-0.3227996
H	1.97325652	1.76229031	1.47815784
H	-0.12687117	-3.16219272	-0.85890922
H	-3.92178863	2.46277883	-0.9135504
H	3.56602106	3.63277191	1.18292547
H	3.18268331	-0.50944381	-0.15389016
H	1.45024829	-5.07996455	-1.03238765
H	-6.10007312	-1.27382658	-0.97538566
H	-3.94849211	-2.50976525	-0.63203574
H	1.10477877	1.80632946	-2.72856599
H	2.68413032	3.70929651	-3.03407309
H	3.92652636	4.62297282	-1.0763684
F	-0.0718474	-0.11430273	-2.37513718

Table S5. XYZ coordinates of the optimized geometry of **2**.

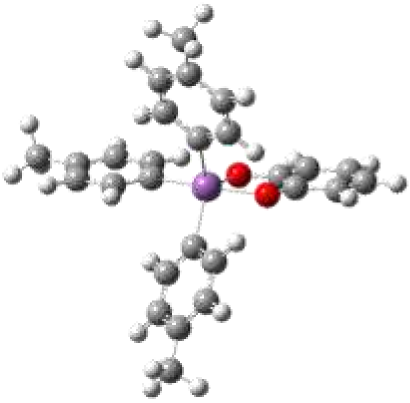

Sb	-0.17133962	0.04587952	-0.16631785	C	-0.85265543	-5.23381012	3.53405353
O	-0.83939488	-0.00530417	-2.08362082	C	1.05391174	5.55933917	3.01185993
O	-2.20352181	0.47090149	0.113881	H	-4.83833609	0.86654521	-0.18920063
C	-2.18405055	0.19439156	-2.22055853	H	2.7711607	-0.05290992	0.94586158
C	-2.91048998	0.44902375	-1.03659693	H	5.03786113	-0.526261	0.12034213
C	1.80874604	-0.32677582	-0.97958247	H	2.00162989	2.35791505	-0.27970973
C	-4.28722978	0.6673141	-1.10575262	H	-6.00463129	0.80115011	-2.40075322
C	2.90880208	-0.29469255	-0.10799939	H	-0.97782045	3.7796663	3.42605614
C	4.42953803	-0.868536	-1.92214959	H	3.48185095	-1.12349136	-3.84301567
C	4.19920247	-0.56125876	-0.57422967	H	1.19822582	-0.65563199	-3.02284264
C	1.34375128	2.65862322	0.53212726	H	-2.34811423	-1.14528094	1.7873843
C	-4.93113869	0.62975547	-2.35001558	H	-4.71215162	0.34803975	-4.48000605
C	-0.31977016	3.46787954	2.61564439	H	-1.46298277	1.69297214	2.20347052
C	3.32732609	-0.89494466	-2.78882936	H	2.44112842	4.47172521	0.9106555
C	2.03368102	-0.63240601	-2.33081357	H	1.25945453	-4.55220676	1.92654226
C	0.252844	1.86027003	0.89441827	H	-2.5880225	-3.14222147	3.23052081
C	-0.39037631	-1.69770891	1.06460085	H	1.51970093	-2.55495843	0.50928092
C	-1.55121772	-1.88086808	1.83349158	H	-2.24077923	-0.04162761	-4.3522472
C	-4.20731205	0.3762364	-3.51670429	H	6.59057894	-0.75233494	-1.77372607
C	-0.58931792	2.28033763	1.93721901	H	5.97771136	-0.79851532	-3.43919893
C	0.77465511	4.27561367	2.26482866	H	5.99286075	-2.26882925	-2.46018832
C	1.59434218	3.85591927	1.21057819	H	-1.48918295	-5.9738173	3.02787419
C	0.46802405	-3.80401955	1.90782407	H	-1.33080282	-5.00074761	4.49357325
C	-1.68408667	-3.01445058	2.6359263	H	0.10945003	-5.7165853	3.74121134
C	0.61715361	-2.67088661	1.10354248	H	0.15811581	6.19145816	3.06941054
C	-0.68121152	-3.99487359	2.68588465	H	1.84709211	6.14131201	2.52905054
C	-2.82388641	0.15595227	-3.45549512	H	1.37080179	5.35743968	4.04476657
C	5.82024133	-1.18333124	-2.42410259				

Table S6. XYZ coordinates of the optimized geometry of **2-F**.

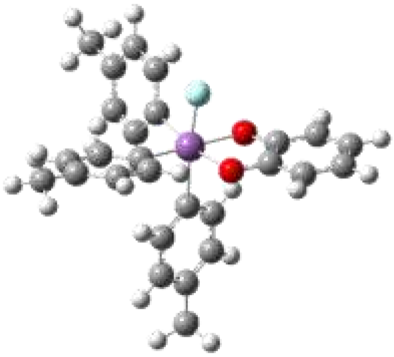

Sb	0.12971492	0.0270839	-0.53919527	C	-1.76463911	-2.20227715	0.64761048
F	-0.11234769	0.13318586	-2.52792296	C	-4.08148487	5.01868364	-0.23415439
O	1.69789833	1.37963137	-0.81897856	C	-4.06286597	-4.99710395	-0.60513689
O	1.69007148	-1.28919794	-0.96539508	H	-1.43905461	5.07007754	-0.94383239
C	-1.77139837	4.06578942	-0.6758657	H	-4.52242737	2.38432522	0.38425399
C	-3.49488576	2.56745659	0.0664968	H	6.05795225	1.35334764	-2.16129466
C	0.61155475	-0.08008828	1.61306581	H	6.04657996	-1.13841967	-2.31348121
C	5.15591084	0.7909572	-1.92063945	H	2.41289119	-1.97356164	3.82226239
C	-3.10082556	3.8666891	-0.27379224	H	3.95908488	-2.4070819	-1.75500938
C	5.14992579	-0.60127548	-2.00473429	H	-2.93369665	0.5007982	0.26856037
C	1.76789727	-1.1689418	3.46530498	H	-0.51449401	1.67465119	2.1806894
C	3.98463945	-1.32008905	-1.69265135	H	0.15841711	3.17584374	-1.05765985
C	2.83256527	0.7743657	-1.20800931	H	0.06599247	1.54926122	4.57321408
C	-2.59119199	1.49997043	0.00155167	H	1.83501624	-1.8434606	1.41890514
C	0.12824951	0.86687835	2.5268298	H	3.98204543	2.56426834	-1.45480075
C	1.28475905	-0.21934051	4.37726103	H	-3.0417029	-3.67768482	1.57077658
C	-1.26435269	1.70053178	-0.4003012	H	0.94710642	0.25940068	6.46765064
C	-0.8666108	3.00358647	-0.74051737	H	2.65571397	0.17763826	6.01777044
C	2.82822401	-0.64937355	-1.28974249	H	1.72618054	-1.3132026	6.2019345
C	0.45789239	0.79798859	3.88561897	H	-1.34761313	-1.77980306	-2.68729502
C	1.44157403	-1.09985457	2.10829876	H	-2.94018003	-3.68435275	-2.72733446
C	-1.27244022	-1.65131619	-0.54276821	H	-1.43840043	-1.80221527	1.60610692
C	3.99688956	1.47754928	-1.52479093	H	-3.62279594	5.92667771	0.18056116
C	-3.10651822	-3.82453253	-0.57952307	H	-4.96040808	4.77721092	0.37720841
C	-2.67138888	-3.26985172	0.6290481	H	-4.44564143	5.27614656	-1.24043084
C	1.6695587	-0.27786229	5.83965478	H	-3.54540159	-5.93679458	-0.85103429
C	-1.7148941	-2.20298793	-1.75684779	H	-4.85048314	-4.86100302	-1.35899406
C	-2.61319407	-3.27166341	-1.77147049	H	-4.55047139	-5.1388152	0.36775117

Table S7. XYZ coordinates of the optimized geometry of **3**.

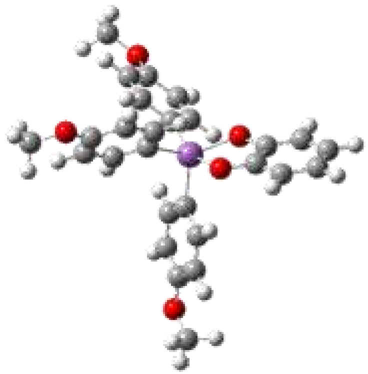

Sb	0.17466165	-0.08897401	-0.49195738	H	-4.74623177	-1.80301092	0.31872023
O	0.89716312	-1.50921775	-1.78915935	H	2.38369298	-2.97186338	-3.46424319
O	1.46557922	1.04409638	-1.65996865	H	-3.00628712	-0.13890465	-0.12915148
C	-3.36110572	-3.4713132	0.42376286	H	2.83911514	1.24855017	0.42681666
C	1.40576555	-0.14333579	1.23956116	H	-1.76277372	-4.9063741	0.44463416
C	-3.71142722	-2.12570436	0.25792323	H	0.00229117	-3.22746856	-0.04084147
C	-1.36692928	-1.54646308	-0.09189059	H	1.63791888	-1.55463395	4.34705404
C	2.63863256	-1.9153916	-3.4202072	H	-2.03913634	4.38049478	-2.2705654
C	-2.71212339	-1.17761874	0.00223056	H	0.17021435	-1.54741274	2.33980367
C	-1.01406797	1.70307601	-0.4065194	H	4.29611991	1.22944635	2.41274737
C	2.57110124	0.63176316	1.28098507	H	-0.64069524	2.327935	-2.44392072
C	-2.01201233	-3.85506784	0.32297825	H	-5.81752575	-3.4546476	1.59454985
C	-1.03115298	-2.90708729	0.06090426	H	-6.13321983	-5.1017626	0.97220817
C	1.88328013	-0.94740073	3.4793515	H	-5.99973984	-3.72959113	-0.16773257
C	-1.92361501	3.71086504	-1.42188595	H	-1.55557546	1.40262836	1.66898239
C	1.06922037	-0.93456461	2.35206353	H	4.23326317	-2.03192748	-4.86653602
C	3.39643582	0.62210149	2.40919534	H	-2.89523166	3.44527861	1.84593582
C	-1.15394185	2.55657346	-1.51411881	H	3.47440868	1.89300316	-3.21864819
C	-5.6207894	-4.15720591	0.77168398	H	4.77720976	0.39409464	-4.74319867
C	-1.65325617	2.04051198	0.79110509	H	5.70503547	0.1956825	3.98105702
C	1.92151505	-1.07693863	-2.56943625	H	5.39664981	0.26918282	5.74166121
C	-2.56165457	4.04499151	-0.21525022	H	4.79257664	1.57351415	4.67698069
C	3.05365238	-0.17033473	3.51477054	H	-4.68479645	4.85501935	1.28301178
C	2.22756534	0.30063525	-2.49907076	H	-4.44703831	6.5371558	0.72345384
C	3.67052007	-1.37868289	-4.20277392	H	-3.22413318	5.76891638	1.77894441
C	-2.42153137	3.20623884	0.89886537	O	-3.28466699	5.19774107	-0.23011174
C	3.25384106	0.82948771	-3.28088793	O	-4.24160452	-4.47666408	0.67857047
C	3.9754802	-0.01775048	-4.13343557	O	3.78082399	-0.25539962	4.66010914
C	4.98335473	0.49356184	4.75492499				
C	-3.94045958	5.59837382	0.96226815				

Table S8. XYZ coordinates of the optimized geometry of **3-F**.

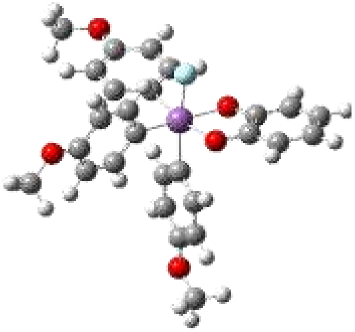

Sb	0.20312354	-0.2523615	-0.73630212	H	-4.56282407	-1.65648763	1.06896236
O	1.52252442	-1.85844389	-0.95631347	H	3.51061636	-3.47150624	-1.64315528
O	1.84575151	0.71955928	-1.58065583	H	-2.77454887	-0.1109129	0.41327748
C	-3.40865085	-3.4402236	0.62242738	H	2.46628513	1.55815888	0.6067091
C	1.00400593	0.1333571	1.28254274	H	-2.03793334	-5.00159777	0.08633896
C	-3.61795994	-2.06104674	0.71617214	H	-0.21932888	-3.41940557	-0.57878962
C	-1.35561581	-1.64627737	-0.12221637	H	0.65504729	-0.8106144	4.5677615
C	3.65834677	-2.42598323	-1.90979987	H	-2.69590599	3.4773432	-3.15805948
C	-2.58991761	-1.18207428	0.34109654	H	-0.30128266	-1.24767872	2.3166851
C	-0.95537878	1.59388657	-0.87809811	H	3.41918142	1.99717834	2.83423871
C	2.05654182	1.03877662	1.46990426	H	-1.36893027	1.36154224	-2.97678576
C	-2.17799597	-3.92447318	0.15574261	H	-5.50842808	-3.36108249	2.34736333
C	-1.16808848	-3.03801676	-0.21133132	H	-6.18128531	-4.85498775	1.62832411
C	1.03759292	-0.29384018	3.68946325	H	-6.12108537	-3.34696365	0.66686939
C	-2.26171505	3.16506584	-2.2100785	H	-0.73511728	2.14688394	1.19501416
C	0.50939195	-0.5284181	2.41984946	H	5.60067431	-2.72840946	-2.80663879
C	2.60061075	1.2887996	2.73763512	H	-2.05207424	4.21107976	1.03469241
C	-1.52099418	1.99069933	-2.10470637	H	4.10011352	1.32856465	-2.83368306
C	-5.59632955	-3.95253236	1.42233502	H	5.89623986	-0.32280196	-3.40499813
C	-1.16536085	2.41715287	0.23230607	H	4.52039324	1.35757576	4.79323751
C	2.65681807	-1.50839877	-1.58488777	H	3.83548963	1.64681803	6.4211367
C	-2.46357684	3.97664876	-1.08396851	H	3.36852421	2.70085227	5.05291693
C	2.08809935	0.61836104	3.85384643	H	-3.97816136	5.45789001	0.62082791
C	2.82850925	-0.13333477	-1.92203671	H	-4.03069914	6.79999311	-0.56132324
C	4.82655143	-2.00103474	-2.56263627	H	-2.48388812	6.3560788	0.22129012
C	-1.91450474	3.60143128	0.14565941	O	-3.21116494	5.11329673	-1.29113532
C	3.99106401	0.27537021	-2.5785708	O	-4.33954873	-4.39620547	0.96014956
C	4.99220948	-0.65686109	-2.89606998	O	2.5431501	0.78373452	5.14261084
C	3.62180468	1.6701652	5.34737272	F	-0.33856247	-0.63850264	-2.62921051
C	-3.42862604	5.96512085	-0.18781188				

Table S9. XYZ coordinates of the optimized geometry of **4**.

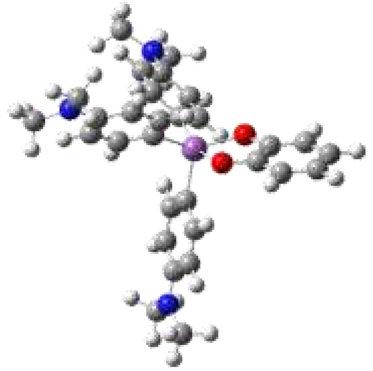

Sb	-0.11996575	0.13354433	0.66813574	C	-2.8526988	1.95354817	4.79414042
O	-0.82994148	-0.75076271	2.38817561	C	-5.87047374	1.18150673	-3.50191193
O	-0.80962417	1.78596821	1.73816561	C	4.8946278	5.15601862	-0.66590328
N	2.80030523	-5.49431535	-0.1757904	C	4.76430911	4.04775022	-2.89861408
N	4.45642636	4.03335801	-1.47863272	C	-5.06454938	-0.996715	-4.42801977
N	-4.83017989	0.17023577	-3.59443221	H	3.88505485	-3.05393818	-0.56390602
C	2.16886631	-4.27208205	-0.01066705	H	-2.18119644	-1.39587389	4.60309861
C	-1.69656006	0.12267652	-0.75065987	H	2.78000232	-0.91820977	-0.23071679
C	2.84910458	-3.0532759	-0.24071643	H	-2.52778959	1.99729809	-0.07522453
C	0.8749579	-1.75296611	0.36407758	H	0.24638728	-5.09520788	0.59580845
C	-2.18990384	-0.33883678	4.34614763	H	-0.83310178	-2.94632372	0.94868539
C	2.21141071	-1.82793156	-0.05089016	H	-2.95914752	-1.75054123	-3.29682232
C	1.42195628	1.45851685	-0.02020697	H	3.37797895	4.04973364	0.99561223
C	-2.61992207	1.17927663	-0.78541222	H	-1.15091638	-1.76077453	-1.66782663
C	0.81724048	-4.19169785	0.40747908	H	-4.35426396	2.02478974	-1.69526976
C	0.19623127	-2.96150115	0.59895047	H	1.62938257	2.56763365	1.8198718
C	-2.87871963	-0.91678752	-2.60718185	H	4.17184414	-5.12949949	-1.77115885
C	2.97896047	3.29640185	0.32443166	H	4.46950959	-6.58272357	-0.78983277
C	-1.84473256	-0.92215371	-1.6740499	H	4.85288589	-4.98609297	-0.13239148
C	-3.65735656	1.19343352	-1.71201669	H	1.44956619	0.62308013	-2.02047501
C	1.98617689	2.44393026	0.80101311	H	-3.38844678	0.26854981	6.03320665
C	4.13377069	-5.54306675	-0.74919381	H	3.16172502	2.11729191	-2.8736987
C	1.8746935	1.36396916	-1.34377606	H	1.56955587	-6.8235914	0.91600021
C	-1.51631953	0.08833488	3.20362791	H	2.69968421	-7.57656972	-0.21680573
C	3.45140272	3.20084485	-1.00702106	H	1.23022043	-6.7855335	-0.8314289
C	-3.81131644	0.14795248	-2.65629393	H	-2.15320146	3.43854747	3.3642446
C	-1.50519886	1.45776419	2.85100708	H	-3.37470155	2.6801034	5.41401106
C	-2.86070351	0.60131463	5.14150418	H	-6.43702265	1.1238306	-2.55755762
C	2.85964605	2.21805397	-1.83647175	H	-6.57216717	1.04993383	-4.32990714
C	2.03148784	-6.72277975	-0.07588913	H	-5.44831836	2.19233006	-3.58316427
C	-2.17371324	2.38797361	3.64668577	H	4.08649047	5.87929566	-0.46338223

H	5.70104521	5.68062357	-1.1856481	H	-4.1818222	-1.23112868	-5.03868738
H	5.29203385	4.81282537	0.2985481	H	-5.891973	-0.78855045	-5.11160072
H	5.09114558	3.05678089	-3.24164862	H	-5.32059905	-1.89479191	-3.84101297
H	5.58655602	4.74538796	-3.07970094				
H	3.90647005	4.35712345	-3.51989864				

Table S10. XYZ coordinates of the optimized geometry of **4-F**.

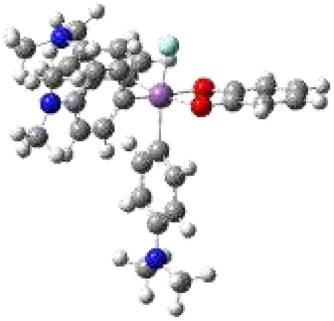

Sb	-0.08971603	-0.09868334	-0.95456416	C	-4.20995218	-2.74316723	4.42477485
O	-1.86319681	0.48424061	-1.90538664	C	6.39006553	-3.24173202	-0.11754561
O	-0.69414745	-1.91679801	-1.79143406	C	5.51933575	-3.48709105	2.16966634
N	1.17455591	6.08152863	0.38674312	C	-4.06709024	-0.39517838	5.13625777
N	5.48572066	-2.76188362	0.91255553	H	2.4511988	3.89811609	1.36807444
N	-3.47016527	-1.49344494	4.39763894	H	-4.03025616	0.64247656	-3.42226122
C	0.87498175	4.72912507	0.12087547	H	1.97214052	1.57531274	0.83769135
C	-1.22082191	-0.564209	0.87680672	H	-1.4684097	-2.65222079	0.41469305
C	1.63239339	3.68331946	0.68761392	H	-0.80851291	5.12738541	-1.19657246
C	0.30939938	1.98705946	-0.47811918	H	-1.26726289	2.78097939	-1.70612081
C	-3.56563269	-0.34218421	-3.39152049	H	-2.53099117	0.94013741	3.64874895
C	1.35263072	2.34786357	0.38285848	H	4.824911	-1.90273567	-1.57247399
C	1.78280632	-1.01301042	-0.31230699	H	-1.23156657	1.44719061	1.66045014
C	-1.68498245	-1.86360714	1.13201715	H	-2.76971101	-3.19549919	2.40475788
C	-0.18253154	4.36560848	-0.74092103	H	2.71080255	-0.86871607	-2.24354026
C	-0.4500052	3.02695587	-1.03313112	H	1.60893748	6.02156253	2.4892811
C	-2.2985932	0.12903067	2.96461526	H	2.16503491	7.46216128	1.59405752
C	4.04643404	-1.779086	-0.82516041	H	3.01391673	5.92538546	1.40145332
C	-1.55081876	0.41824111	1.81931902	H	1.18690887	-1.35034355	1.73485398
C	-2.43053473	-2.17224127	2.27173586	H	-5.00389345	-1.27444771	-4.70800221
C	2.84216755	-1.19189301	-1.21473723	H	3.2800247	-2.36431382	2.4417375
C	2.02246423	6.37801225	1.52668277	H	-0.05706803	7.0799519	-1.00073218
C	1.98609174	-1.45079932	1.00226267	H	0.54934525	8.06924691	0.33065052
C	-2.41644703	-0.51095949	-2.6154868	H	-0.788856	6.92238842	0.61347066
C	4.25543987	-2.20657864	0.50435378	H	-1.83673281	-3.82440138	-3.23154064
C	-2.74879817	-1.18170662	3.22531436	H	-3.90377324	-3.51390627	-4.6138745
C	-1.79101787	-1.79251622	-2.55783631	H	-4.97393552	-2.82062313	3.6287381
C	-4.10461414	-1.4219291	-4.10985471	H	-4.71082116	-2.84033142	5.39440224
C	3.18852969	-2.03664386	1.41028955	H	-3.53108435	-3.59839867	4.32088497
C	0.16452071	7.07623332	0.07349177	H	5.95447254	-4.03720542	-0.75072132
C	-2.33240271	-2.85588714	-3.28339657	H	7.29504192	-3.63624191	0.35753983
C	-3.49073912	-2.67307493	-4.05656248	H	6.6984423	-2.42114302	-0.77676849

H	5.25544708	-2.82730421	3.00550671
H	6.53862749	-3.84723688	2.34749899
H	4.83413337	-4.3552815	2.19675155
H	-3.29440679	0.29417144	5.49852421
H	-4.5851	-0.79528835	6.01491463
H	-4.79218011	0.1932248	4.54293817
F	0.77944398	0.23868134	-2.73337536

Table S111. XYZ coordinates of the optimized geometry of **5**.

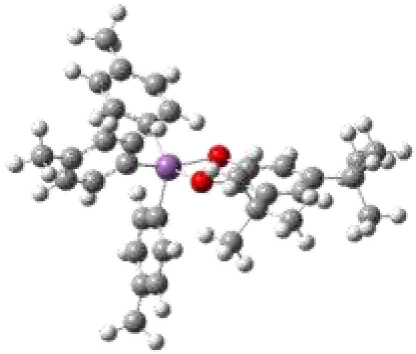

Sb	0.927721	0.186421	-0.080769	C	-6.522163	1.290912	0.690998
O	-0.640365	-1.126827	-0.332834	C	-6.423453	1.276334	-1.84025
O	-0.652957	1.483032	-0.357674	C	-5.482859	3.208817	-0.547354
C	1.135753	0.312358	2.030926	C	-3.136454	-2.793273	-0.440899
C	0.533986	-0.661673	2.84737	C	-4.575126	-3.358385	-0.497013
C	0.647812	-0.575381	4.23751	C	-2.461428	-3.336158	0.849042
C	1.36117	0.474155	4.844699	C	-2.367872	-3.317641	-1.68554
C	1.954401	1.442948	4.018257	H	-0.027738	-1.476062	2.401007
C	1.845642	1.368456	2.625033	H	0.173312	-1.328891	4.860088
C	1.497966	0.544901	6.34923	H	2.502357	2.26581	4.468668
C	2.156929	1.734987	-0.943346	H	2.30674	2.135751	2.010393
C	1.699475	3.063997	-1.046171	H	2.272088	-0.145873	6.710049
C	2.539141	4.054789	-1.56175	H	0.562185	0.271235	6.849079
C	3.843944	3.755719	-1.994884	H	1.776392	1.55086	6.678657
C	4.288597	2.428288	-1.892043	H	0.691584	3.307603	-0.736379
C	3.458044	1.426174	-1.375795	H	2.174754	5.076215	-1.63321
C	4.730429	4.832542	-2.579282	H	5.292	2.173082	-2.221679
C	2.142126	-1.520349	-0.617709	H	3.830095	0.408824	-1.317878
C	1.886632	-2.26253	-1.783887	H	4.456862	5.047883	-3.621062
C	2.699343	-3.348302	-2.121336	H	5.783135	4.532595	-2.57179
C	3.781684	-3.728087	-1.30662	H	4.640653	5.771569	-2.021618
C	4.029715	-2.982468	-0.143554	H	1.046093	-2.007038	-2.420461
C	3.222223	-1.890104	0.200268	H	2.488527	-3.912644	-3.025855
C	4.636901	-4.923156	-1.665255	H	4.859222	-3.258621	0.501787
C	-1.899966	0.872635	-0.383451	H	3.436848	-1.339302	1.112077
C	-1.90606	-0.531647	-0.377288	H	5.587795	-4.911672	-1.12307
C	-3.115264	-1.249669	-0.432258	H	4.858843	-4.94975	-2.738158
C	-4.294275	-0.481637	-0.483417	H	4.127661	-5.864095	-1.416959
C	-4.310078	0.92754	-0.487733	H	-5.241711	-1.002209	-0.521472
C	-3.082735	1.605988	-0.438926	H	-3.018469	2.685653	-0.443003
C	-5.662429	1.673141	-0.545283	H	-6.71827	0.214153	0.727711

H	-7.488538	1.810239	0.659062
H	-6.010848	1.571887	1.618978
H	-5.841465	1.546844	-2.728853
H	-7.389063	1.795709	-1.889479
H	-6.616994	0.199248	-1.880037
H	-4.976432	3.557072	0.360134
H	-6.464915	3.694176	-0.589732
H	-4.90524	3.546067	-1.415641
H	-5.10187	-3.042645	-1.40496
H	-5.167994	-3.051255	0.372437
H	-4.53151	-4.45367	-0.501004
H	-2.47752	-4.433644	0.849188
H	-2.996813	-2.986858	1.740317
H	-1.42174	-3.005357	0.909998
H	-2.841278	-2.961191	-2.608346
H	-2.377115	-4.41522	-1.698676
H	-1.329322	-2.97893	-1.670784

Table S12. XYZ coordinates of the optimized geometry of **5-F**.

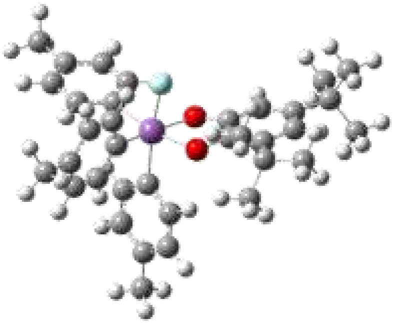

Sb	0.851806	0.141963	-0.325805	C	-6.61387	1.350799	0.826173
O	-0.766066	-1.167644	-0.090433	C	-6.490199	1.335734	-1.701795
O	-0.737841	1.48724	-0.147758	C	-5.533363	3.251477	-0.39959
F	0.378816	0.087024	-2.278937	C	-3.255069	-2.773882	-0.293188
C	1.151342	0.220319	1.856448	C	-4.695538	-3.330845	-0.384167
C	0.396715	-0.597444	2.720119	C	-2.605936	-3.345296	0.997583
C	0.571042	-0.536564	4.107116	C	-2.465111	-3.281619	-1.531823
C	1.501451	0.3445	4.684791	H	-0.330085	-1.281507	2.296684
C	2.257159	1.157791	3.826539	H	-0.024877	-1.180742	4.751111
C	2.08404	1.09849	2.436797	H	2.986927	1.846565	4.2482
C	1.662334	0.431064	6.18823	H	2.682171	1.747457	1.804297
C	2.187891	1.790066	-0.817915	H	1.570681	-0.555096	6.659677
C	1.692023	3.100499	-0.95928	H	0.89472	1.076468	6.639145
C	2.547463	4.153628	-1.298814	H	2.638862	0.846904	6.462317
C	3.920743	3.937684	-1.511563	H	0.634103	3.279573	-0.804559
C	4.412799	2.631521	-1.36974	H	2.143963	5.159263	-1.402346
C	3.559151	1.572197	-1.030877	H	5.472155	2.439168	-1.528456
C	4.834408	5.079551	-1.905864	H	3.970138	0.570667	-0.937186
C	2.170745	-1.561766	-0.685482	H	4.720048	5.338979	-2.968134
C	2.246234	-2.145355	-1.965512	H	5.88735	4.821885	-1.74412
C	3.084572	-3.238556	-2.205466	H	4.616812	5.986876	-1.328593
C	3.876342	-3.787613	-1.181112	H	1.641147	-1.730962	-2.763062
C	3.805059	-3.203029	0.092117	H	3.125866	-3.673469	-3.202411
C	2.963212	-2.108969	0.337937	H	4.410385	-3.607821	0.900874
C	4.760467	-4.98924	-1.442193	H	2.924319	-1.68552	1.336882
C	-1.969034	0.876352	-0.201445	H	5.525164	-5.098455	-0.664707
C	-1.994458	-0.539351	-0.182745	H	5.271622	-4.909977	-2.40969
C	-3.220463	-1.230114	-0.262642	H	4.177583	-5.921241	-1.461507
C	-4.39639	-0.454627	-0.325053	H	-5.349535	-0.966529	-0.375764
C	-4.391809	0.952507	-0.329514	H	-3.071982	2.691394	-0.285311
C	-3.152488	1.611954	-0.274124	H	-6.81833	0.275488	0.861741
C	-5.733053	1.718078	-0.400024	H	-7.575948	1.881088	0.785934

H	-6.105733	1.625751	1.757908
H	-5.892726	1.598701	-2.582384
H	-7.450804	1.866788	-1.762125
H	-6.692006	0.260225	-1.744281
H	-5.025702	3.588519	0.511253
H	-6.508394	3.753259	-0.450168
H	-4.939535	3.577351	-1.260926
H	-5.204742	-2.992158	-1.294434
H	-5.302026	-3.034817	0.480754
H	-4.657896	-4.427356	-0.408714
H	-2.624245	-4.444156	0.978079
H	-3.15549	-3.008866	1.88615
H	-1.56899	-3.012004	1.076815
H	-2.936356	-2.927431	-2.457012
H	-2.451959	-4.380771	-1.550227
H	-1.437306	-2.913216	-1.503224

Table S132. XYZ coordinates of the optimized geometry of **6**.

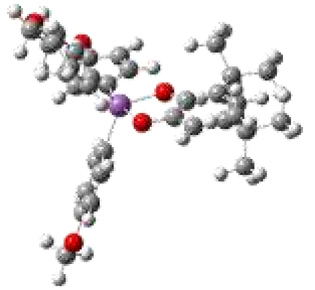

Sb	-0.61817079	0.13226054	0.15611273	C	-2.20558337	4.17472025	-0.68198561
O	0.70262736	-0.65597993	-1.17598842	C	-6.69460783	-1.44434905	-3.45682814
O	1.19682969	0.51263462	1.11840509	C	4.15963733	-2.17399386	-3.66078256
O	-2.31866249	-3.00478134	5.38709388	C	-3.28139576	-4.092893	5.37104943
O	-1.82005611	6.3505163	0.14623608	C	6.26690402	-0.77328173	2.02599351
O	-5.29664664	-1.6688217	-3.77963794	C	6.1243296	-0.04299415	0.66206023
C	-2.19342384	-0.50150135	-1.19240549	C	7.19418925	-0.61075368	-0.29881344
C	-3.5289664	-0.26665626	-0.82043834	C	6.41215947	1.47073189	0.86136403
C	2.05859849	-0.55351944	-0.81893317	H	-3.75978668	0.2118062	0.12826684
C	-1.00836934	2.23559597	0.17391128	H	0.46832525	2.6573762	1.68791516
C	-1.22894313	-0.94305925	1.90152032	H	-2.6209113	-2.23122223	0.8603937
C	2.30543015	0.07644377	0.41639134	H	-0.92419317	-1.31725313	-2.73839578
C	-0.29657937	3.07713683	1.04664384	H	0.11420596	0.11437612	3.22869332
C	-2.17803513	-1.9725968	1.81564283	H	-2.81684565	-1.98243928	-4.21984137
C	-1.94396525	-1.12646119	-2.4321266	H	-5.61208904	-0.44111237	-1.3337732
C	-0.65467391	-0.64427542	3.1569898	H	3.74665641	0.72737222	1.84536734
C	-2.9967264	-1.50138449	-3.26529171	H	0.01842118	5.08081294	1.75348064
C	-4.59525772	-0.63823376	-1.65094584	H	-0.61861732	-1.12706692	5.26609858
C	-4.32358764	-1.25810381	-2.8781111	H	5.24323619	-1.2146378	-1.69531806
C	3.60262258	0.23917411	0.88842359	H	-2.51040092	2.17865259	-1.39141101
C	3.11048404	-1.03498286	-1.61261447	H	-1.30211577	7.0438754	2.07450561
C	-0.54291064	4.45453816	1.07106177	H	-1.54053073	8.25771175	0.78406373
C	-1.50089802	5.00246286	0.20611244	H	-0.04502258	7.27910294	0.81910775
C	-1.04591266	-1.34403265	4.29411452	H	-3.29523574	-3.48567531	2.85854073
C	2.8506111	-1.72580797	-2.96864812	H	2.50136215	-3.71536379	-2.11611845
C	4.4162159	-0.85306948	-1.10419856	H	1.76335206	-3.47413437	-3.71362023
C	-2.00345558	-2.36767982	4.19635212	H	1.02836144	-2.73858764	-2.26928348
C	-1.96175341	2.80340383	-0.69621359	H	1.18575948	-0.38823633	-3.49294654
C	4.68767599	-0.22939856	0.12447742	H	1.90981538	-1.23133587	-4.88306834
C	-1.1225608	7.27987427	1.01929743	H	2.75804784	0.13363892	-4.12672711
C	-2.56710779	-2.68974194	2.95497713	H	-2.92949848	4.62756589	-1.34929049
C	1.97576731	-2.99171572	-2.75074253	H	-6.98279967	-1.97794222	-2.54342636
C	2.12541828	-0.73928874	-3.92579576	H	-7.24962565	-1.84196135	-4.30548596

H	-6.90965361	-0.37549043	-3.34185217	H	7.28042027	-0.64113718	2.42590549
H	4.81850947	-1.32496553	-3.87616537	H	6.07948881	-1.8471007	1.91082123
H	3.91486554	-2.65622808	-4.61436981	H	7.06490739	-1.68756029	-0.45683123
H	4.71439913	-2.89847989	-3.05355953	H	8.19148903	-0.45310818	0.12831103
H	-3.3514293	-4.42502194	6.40592783	H	7.16611137	-0.11285194	-1.27480414
H	-2.93571941	-4.91897327	4.73900977	H	6.32892227	2.00863538	-0.09012355
H	-4.26308673	-3.74920968	5.0248271	H	7.42673535	1.618151	1.25313618
H	5.55815926	-0.3859927	2.76552481	H	5.70800924	1.92433796	1.56684892

Table S14. XYZ coordinates of the optimized geometry of **6-F**.

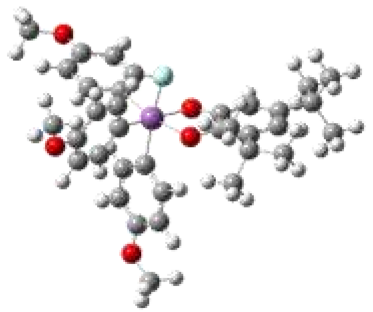

Sb	0.616986	0.152169	-0.336197	C	-4.602256	1.022641	-0.740065
O	-1.032119	-1.130732	-0.177713	C	4.624351	-5.625478	-2.150983
O	-0.962262	1.520133	-0.325412	C	-4.627212	-0.383338	-0.68798
F	0.288281	0.029691	-2.316604	C	0.178963	-0.101813	6.933141
O	4.605435	4.957286	-1.719314	C	-2.658693	-3.273382	-1.6615
O	1.036161	0.667354	6.05938	C	-4.962799	-3.255977	-0.66878
O	4.482914	-4.925604	-0.893824	C	-5.703529	3.331252	-0.96519
C	2.003617	1.768344	-0.778865	C	-6.593549	1.384363	-2.268665
C	1.935925	-1.573892	-0.543098	C	-5.9243	1.801149	-0.929828
C	0.756725	0.295279	1.855823	C	-6.894089	1.485508	0.242415
C	4.281202	2.553832	-1.222363	H	5.336895	2.326283	-1.318414
C	-0.013066	-0.391009	4.080353	H	-0.672823	-1.00664	4.681527
C	2.072289	-2.214311	-1.786377	H	1.515632	-1.830741	-2.633003
C	-2.242208	-0.491405	-0.376693	H	2.573944	-1.623018	1.524731
C	2.665189	-2.087768	0.548105	H	2.981763	-3.802734	-2.918798
C	-2.195368	0.922315	-0.442463	H	0.478826	3.287233	-0.887641
C	2.905338	-3.331378	-1.945349	H	-0.77829	-1.166918	2.224534
C	1.540425	3.087037	-0.974363	H	2.307231	1.806851	1.878764
C	-0.072306	-0.482708	2.681299	H	2.419343	1.967695	4.358116
C	1.657107	1.181506	2.482575	H	3.764123	0.516571	-0.780828
C	0.892173	0.496113	4.673197	H	6.480826	4.365282	-0.948384
C	1.729606	1.284191	3.87417	H	6.443903	5.718405	-2.116536
C	3.620732	-3.818973	-0.845346	H	6.240149	4.036509	-2.688758
C	3.377743	1.523483	-0.91165	H	4.065194	-3.597157	1.24052
C	6.025732	4.740498	-1.874294	H	2.080043	5.135874	-1.438826
C	3.79705	3.85238	-1.407145	H	-3.264064	2.746209	-0.664306
C	3.502825	-3.19695	0.403566	H	-1.938291	-2.925522	0.990564
C	-3.469551	-1.169997	-0.517601	H	-3.006419	-4.346268	0.86935
C	2.426313	4.119417	-1.284318	H	-3.575895	-2.873387	1.689712
C	-3.360463	1.668893	-0.621689	H	5.321107	-6.440794	-1.951905
C	-2.972593	-3.247752	0.852622	H	5.035059	-4.973291	-2.932516
C	-3.524105	-2.713404	-0.49806	H	3.665998	-6.03538	-2.494357

H	-5.581787	-0.885265	-0.787566	H	-5.047337	3.621494	-1.793359
H	0.341147	-1.180594	6.811216	H	-5.255191	3.692472	-0.032762
H	-0.880983	0.122374	6.757939	H	-6.665584	3.842509	-1.099487
H	0.45395	0.196872	7.945603	H	-7.53952	1.92502	-2.414129
H	-3.060666	-2.94622	-2.62838	H	-6.807832	0.310621	-2.290053
H	-2.659741	-4.372601	-1.641467	H	-5.931864	1.610083	-3.112916
H	-1.630223	-2.916052	-1.574603	H	-6.44922	1.786271	1.198229
H	-5.622605	-2.922507	0.141751	H	-7.115341	0.414409	0.298928
H	-4.939399	-4.353042	-0.653298	H	-7.843545	2.024982	0.116598
H	-5.403694	-2.943192	-1.622875				

Table S15. XYZ coordinates of the optimized geometry of **7**.

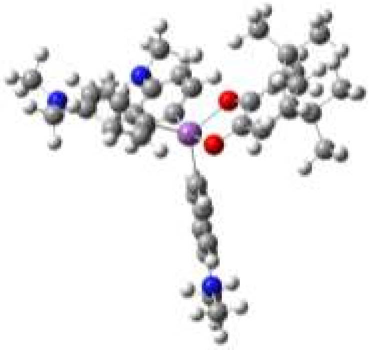

Sb	-0.400425	0.000063	0.2006	C	-2.633012	-6.342199	2.831295
O	0.814107	0.000635	-1.441801	C	6.654064	1.267956	0.909504
O	1.503003	0.001198	1.088177	C	-0.336931	3.349918	3.077928
N	-1.605222	-5.424564	3.319708	C	1.968482	-1.271145	-4.010718
N	-5.47819	-0.002624	-3.716336	C	1.968072	1.272918	-4.010883
N	-1.612092	5.423457	3.319233	C	4.042151	0.001178	-4.601038
C	-0.334352	-3.348261	3.079545	C	-0.073172	2.167957	2.391076
C	-1.343199	-4.243764	2.637954	C	-5.306148	-0.000555	-5.167849
C	-0.809732	-1.813328	1.244837	C	7.357133	0.002661	-1.138284
C	-0.072125	-2.166032	2.392562	C	-0.837239	5.774969	4.508468
C	-1.803785	-2.701214	0.795897	C	-2.642925	6.338417	2.832236
C	-2.067957	-3.892038	1.470962	C	-6.823554	-0.004452	-3.146765
C	4.894917	0.001839	-0.3985	H	0.256493	-3.580757	3.956275
C	2.544292	0.001334	0.184094	H	0.728905	-1.520054	2.730169
C	2.192449	0.001082	-1.181273	H	-2.378557	-2.470816	-0.094423
C	3.878745	0.00169	0.575391	H	-2.835865	-4.549589	1.083951
C	3.174009	0.00123	-2.183846	H	4.104295	0.001848	1.635773
C	-2.074373	-0.001034	-1.167851	H	5.29272	0.001749	-2.504151
C	4.519574	0.001616	-1.751467	H	-2.384443	2.466372	-0.092199
C	-1.808718	2.698459	0.797064	H	-5.488512	-0.005103	-1.016516
C	-4.365987	-0.002054	-2.881837	H	-2.890434	0.001274	-4.479456
C	-0.811874	1.813046	1.24473	H	0.23915	-5.882216	4.286063
C	-4.505572	-0.00355	-1.470615	H	-1.191874	-6.724942	4.904896
C	-3.049829	0.00003	-3.408494	H	-0.937212	-5.017151	5.29963
C	2.798056	0.001042	-3.681322	H	-3.540274	-0.00429	0.432826
C	6.372546	0.002118	0.053779	H	-0.946275	0.002075	-3.004815
C	-0.831363	-5.773884	4.510232	H	6.010581	-1.302219	1.793686
C	-3.382272	-0.003049	-0.643321	H	7.698667	-1.272237	1.247922
C	-1.936412	0.000515	-2.567913	H	6.475234	-2.173079	0.323157
C	-1.34855	4.242925	2.637619	H	-2.844601	4.544553	1.086046
C	6.654615	-1.264035	0.908883	H	-3.62229	-5.864904	2.800863
C	-2.074498	3.888972	1.472039	H	-2.693926	-7.201098	3.5012

H	-2.404944	-6.713319	1.822079	H	-4.761241	0.889176	-5.513396
H	6.474299	2.177225	0.324238	H	-6.287476	-0.001478	-5.64477
H	7.698118	1.276431	1.248524	H	-4.75857	-0.887793	-5.515527
H	6.010068	1.305459	1.79436	H	7.230144	-0.885824	-1.767352
H	0.254835	3.584163	3.95357	H	8.38762	0.002971	-0.763603
H	2.56462	-2.173741	-3.829715	H	7.229476	0.891255	-1.767063
H	1.67081	-1.26547	-5.067602	H	-0.940427	5.018465	5.298445
H	1.069398	-1.324591	-3.392593	H	-1.199066	6.725538	4.903115
H	1.069089	1.326269	-3.392593	H	0.232725	5.885319	4.282686
H	1.670192	1.266906	-5.067705	H	-2.417641	6.709381	1.822343
H	2.563983	2.175725	-3.830209	H	-2.704487	7.197628	3.501683
H	4.663843	0.890505	-4.445709	H	-3.631188	5.858871	2.804025
H	3.715617	0.001047	-5.647762	H	-7.003907	-0.893615	-2.52594
H	4.664126	-0.887928	-4.445569	H	-7.555364	-0.004947	-3.956003
H	0.729818	1.523879	2.727646	H	-7.006068	0.883666	-2.525042

Table S16. XYZ coordinates of the optimized geometry of **7-F**.

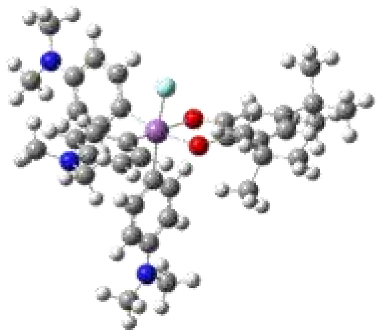

Sb	0.384637	-0.111495	0.474606	C	4.546119	-5.588479	2.84187
O	-1.270646	1.193437	0.387393	C	-6.29666	-2.751204	-0.216248
O	-1.210656	-1.462618	0.453389	C	-0.263829	-1.153001	-3.844568
N	4.493867	-4.958516	1.529356	C	-2.936466	3.338121	1.8211
N	4.300787	4.938944	1.269025	C	-3.189496	3.296957	-0.706201
N	0.605142	-0.321715	-5.986727	C	-5.211661	3.318091	0.770722
C	3.463682	-3.32921	0.009658	C	-0.298686	-1.102128	-2.448613
C	3.599641	-3.901751	1.294827	C	3.761809	6.291986	1.234117
C	1.78212	-1.742047	0.822203	C	-7.413546	-0.947796	1.118614
C	2.571034	-2.274601	-0.211074	C	-0.292228	-1.213451	-6.709396
C	1.91908	-2.316543	2.099552	C	1.382144	0.66622	-6.722299
C	2.801108	-3.373069	2.336822	C	5.67994	4.723989	1.684413
C	-4.856051	-0.957729	0.846783	H	4.044795	-3.705206	-0.824539
C	-2.441029	-0.862774	0.581135	H	2.486215	-1.869709	-1.215281
C	-2.482587	0.557122	0.542943	H	1.317948	-1.925451	2.911996
C	-3.608343	-1.603806	0.73605	H	2.862346	-3.786089	3.33716
C	-3.713439	1.230066	0.661487	H	-3.52093	-2.685052	0.767007
C	1.698794	1.602027	0.721756	H	-5.829591	0.953118	0.891642
C	-4.880361	0.44427	0.806438	H	1.89306	1.435337	-1.942059
C	1.286926	0.697474	-2.45972	H	4.953939	2.322225	1.474773
C	3.443891	3.83984	1.094071	H	1.661365	5.017333	0.689201
C	0.474385	-0.178602	-1.719326	H	4.581539	-5.973223	-0.348461
C	3.917171	2.514117	1.223435	H	5.890333	-6.329775	0.799267
C	2.075246	4.020078	0.784343	H	5.874093	-4.788685	-0.074624
C	-3.76977	2.773061	0.636989	H	3.456153	0.423876	1.155766
C	-6.134525	-1.810045	1.009553	H	0.182688	3.091011	0.37888
C	5.239771	-5.537668	0.420771	H	-5.152369	-3.327513	2.265982
C	3.056122	1.427188	1.03852	H	-6.923102	-3.299251	2.420992
C	1.22941	2.922551	0.603947	H	-5.931243	-2.029212	3.180773
C	0.56027	-0.271635	-4.583349	H	1.974144	1.367281	-4.376656
C	-6.028564	-2.672021	2.298086	H	4.816747	-4.86722	3.626008
C	1.335264	0.661596	-3.857973	H	5.304988	-6.374622	2.833438

H	3.584568	-6.043348	3.129308	H	4.576323	7.007064	1.37285
H	-6.393307	-2.165373	-1.138084	H	3.012117	6.468685	2.022345
H	-7.192149	-3.379787	-0.108414	H	-7.373019	-0.279782	1.986651
H	-5.428536	-3.408815	-0.328301	H	-8.290867	-1.597861	1.232226
H	-0.8858	-1.879273	-4.355173	H	-7.560903	-0.334462	0.222115
H	-3.377384	3.030835	2.777645	H	-0.129416	-2.263183	-6.427931
H	-2.919896	4.437169	1.787239	H	-0.103284	-1.121663	-7.781772
H	-1.911918	2.962533	1.78035	H	-1.354516	-0.983374	-6.526933
H	-2.170289	2.931265	-0.848132	H	1.026647	1.696273	-6.554357
H	-3.182851	4.396453	-0.716673	H	1.314764	0.453251	-7.791975
H	-3.802274	2.950004	-1.548091	H	2.443724	0.634279	-6.439495
H	-5.852667	2.978922	-0.052335	H	5.753902	4.232046	2.668152
H	-5.186847	4.415275	0.747333	H	6.191314	5.687617	1.746944
H	-5.675162	3.013057	1.716726	H	6.225391	4.099926	0.962418
H	-0.941679	-1.788408	-1.907897	F	0.085897	-0.055914	2.463488
H	3.283073	6.51028	0.269224				

Table S17. XYZ coordinates of the optimized geometry of **8**.

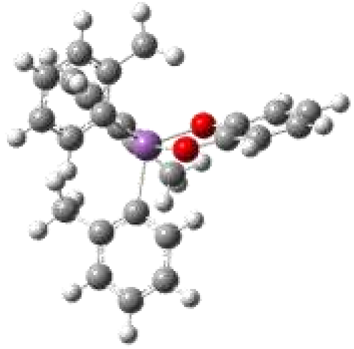

Sb	-0.01994	0.019606	-0.028208	H	1.407753	5.012884	-3.109335
O	-1.877432	0.192121	-0.984536	H	-1.266316	-4.136541	-2.873223
O	-1.324991	-0.27816	1.527358	H	-4.508826	0.290007	-1.631421
C	0.498073	1.831277	-1.073326	H	1.058918	-4.923043	-3.334835
C	1.483604	0.051913	1.549964	H	2.987034	-3.63825	-2.470694
C	-2.971285	0.016765	-0.157704	H	2.149117	2.770862	-3.911536
C	0.48087	-1.703319	-1.224101	H	0.065189	5.204209	-1.037369
C	-2.678699	-0.241016	1.196771	H	4.248036	0.334768	4.282246
C	1.243971	1.721462	-2.257297	H	-6.359093	-0.04977	0.020126
C	1.806825	-2.137013	-1.482217	H	-3.448406	-0.619324	3.16476
C	-0.612329	-2.42744	-1.735686	H	-5.83343	-0.502934	2.407416
C	1.155499	4.119608	-2.546654	H	2.136446	2.02335	0.947566
C	-0.412513	-3.589235	-2.487683	H	2.860463	-1.70756	4.119096
C	0.049168	3.093724	-0.620705	H	3.878107	2.218852	2.685005
C	-4.295834	0.08728	-0.58783	C	3.046701	-1.428272	-0.973942
C	0.888708	-4.026992	-2.74622	H	2.984833	-0.340855	-1.080203
C	1.977018	-3.301616	-2.254476	H	3.225304	-1.634235	0.087254
C	1.575077	2.865045	-2.995417	H	3.927413	-1.763884	-1.530166
C	0.399334	4.227006	-1.374083	H	1.554857	0.747496	-2.622745
C	3.48091	0.264311	3.517298	C	-0.812784	3.240091	0.614882
C	-5.325587	-0.102986	0.347297	H	-1.803967	2.796922	0.456705
C	-3.696542	-0.427285	2.126898	H	-0.370364	2.748086	1.490231
C	-5.030352	-0.358228	1.692127	H	-0.958003	4.294212	0.868752
C	2.280848	1.204497	1.645211	C	0.871283	-2.29899	2.412577
C	1.685156	-1.022968	2.453797	H	-0.186238	-2.089942	2.598412
C	2.694137	-0.889031	3.424349	H	0.937828	-2.79352	1.435227
C	3.275663	1.317972	2.625097	H	1.226149	-3.008187	3.166358
H	-1.618016	-2.064563	-1.565117				

Table S18. XYZ coordinates of the optimized geometry of **8-F**.

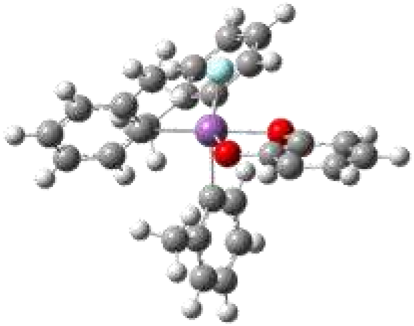

Sb	-0.00655573	-0.04459948	-0.35639192	H	0.14760853	-0.81151748	5.72793901
O	1.56431499	-1.44422488	-0.31235366	H	-0.96993031	-4.61472595	-2.61708219
O	1.67486294	1.22078508	-0.4144611	H	3.91756064	-2.70825592	-0.46641981
C	0.01066429	-0.13035516	1.87019116	H	-3.2828175	-5.03986015	-1.75701298
C	-1.25768652	1.76420737	-0.58327667	H	-4.33217846	-3.39353848	-0.22989367
C	2.80686547	-0.87679364	-0.44203057	H	-0.37180225	-2.67812852	4.14278807
C	-1.35236769	-1.78901041	-0.70436738	H	0.61014617	1.45298925	4.83605096
C	2.86905481	0.54170033	-0.50052902	H	-3.6568433	4.86564789	-0.65451328
C	-0.2273292	-1.4149341	2.39744607	H	6.13412424	-1.56413477	-0.69510463
C	-2.66071975	-2.03577313	-0.21451313	H	4.12739288	2.26624505	-0.68103443
C	-0.77293545	-2.74114218	-1.5696503	H	6.23990524	0.92457656	-0.80397043
C	0.10624	-0.63163109	4.65652132	H	-2.22478232	1.32035458	1.29007003
C	-1.4507112	-3.90357258	-1.95055678	H	-1.97980306	4.53159609	-2.44480574
C	0.32699737	0.92390317	2.76860185	H	-3.7653434	3.23921719	1.24817388
C	3.98272738	-1.62588269	-0.5141917	C	-3.39863777	-1.10118863	0.72054323
C	-2.74097124	-4.14185309	-1.47051418	H	-2.79451119	-0.84929282	1.59840317
C	-3.32923959	-3.21288008	-0.61120289	H	-3.66517259	-0.16216419	0.22420683
C	-0.18716794	-1.67321162	3.77225466	H	-4.32571282	-1.56675192	1.07440644
C	0.36367597	0.64464985	4.15062289	H	-0.43267063	-2.2343398	1.71582924
C	-2.99378918	4.00471309	-0.62268161	C	0.63229863	2.34045624	2.32367274
C	5.22202964	-0.97491117	-0.64263873	H	1.23268496	2.34283762	1.4104268
C	4.10128055	1.181941	-0.63432281	H	-0.28646497	2.90076444	2.10867007
C	5.28180741	0.42104341	-0.70312619	H	1.1731904	2.87815724	3.11146132
C	-2.18984098	1.99768157	0.44401619	C	-0.15055522	2.63212802	-2.76075967
C	-1.16767101	2.70934089	-1.64250791	H	0.85290116	2.47982932	-2.35409666
C	-2.05063194	3.8089808	-1.63498595	H	-0.34265486	1.78217225	-3.41998919
C	-3.0586226	3.09636573	0.43487015	H	-0.16708138	3.55650181	-3.35040709
H	0.22825586	-2.56311969	-1.93734203	F	0.22959245	-0.13075548	-2.34528406

Table S19. Fluoride anion affinities (FIA) calculation details.

Compound	HF (Hartree)	H_{corr}(Hartree)	H	FIA (Hartree)	FIA (kJ/mol)
**1**	-1317.0515430	0.387179	-1316.6643640	0.1084313	284.7
**[1-F]$^-$**	-1417.0479565	0.388828	-1416.6591285		
**2**	-1435.0392624	0.475674	-1434.5635884	0.1047524	275.0
**[2-F]$^-$**	-1535.0319470	0.477273	-1534.5546740		
**3**	-1660.7301756	0.492166	-1660.2380096	0.1041931	273.6
**[3-F]$^-$**	-1760.7225669	0.494031	-1760.2285359		
**4**	-1719.0727826	0.621140	-1718.4516426	0.0929418	244.0
**[4-F]$^-$**	-1819.0533166	0.622399	-1818.4309176		
**5**	-1749.6318147	0.713510	-1748.9183047	0.1071978	281.5
**[5-F]$^-$**	-1849.6270867	0.715251	-1848.9118357		
**6**	-1975.3248383	0.730406	-1974.5944323	0.1050460	275.8
**[6-F]$^-$**	-2075.3178475	0.732036	-2074.5858115		
**7**	-2033.6714804	0.859424	-2032.8120564	0.0900890	236.5
**[7-F]$^-$**	-2133.6488636	0.860385	-2132.7884786		
**8**	-1435.0254892	0.475546	-1434.5499432	0.0988804	259.6
**[8-F]$^-$**	-1535.0129708	0.477814	-1534.5351568		

The enthalpy of the fluoride anion in the gas phase was determined as -99.8863332 Hartree

4.5 Topographic Steric Maps of Stiborane Catalyst

Topographic steric maps of the stiborane were under consideration in their fluoride adducts [Sb-F]$^-$ (R = 3.50 Å). The isocontour scheme is displayed in Å; red and blue zones indicate the more- and less-hindered zones with respect to the origin. Topographic steric maps have been obtained from the SambVca 2.1.

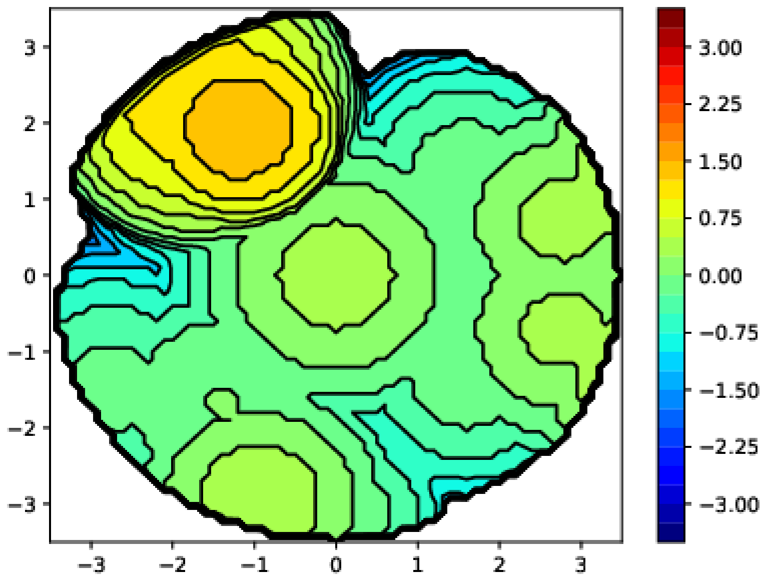

Figure S45. Topographic steric maps of **1**.

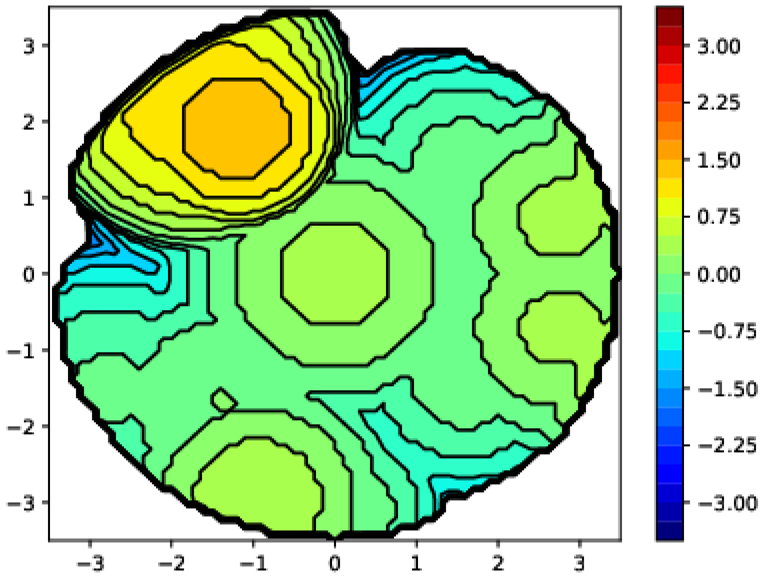

Figure S46. Topographic steric maps of **2**.

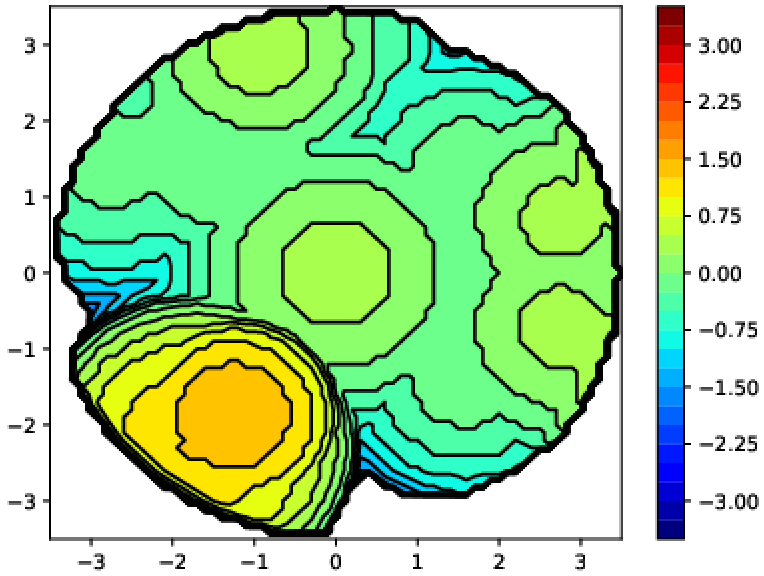

Figure S47. Topographic steric maps of **3**.

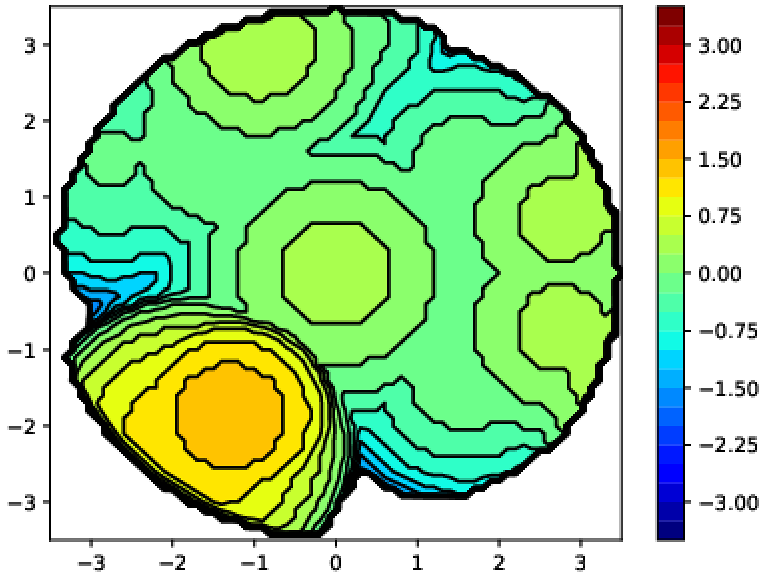

Figure S48. Topographic steric maps of **4**.

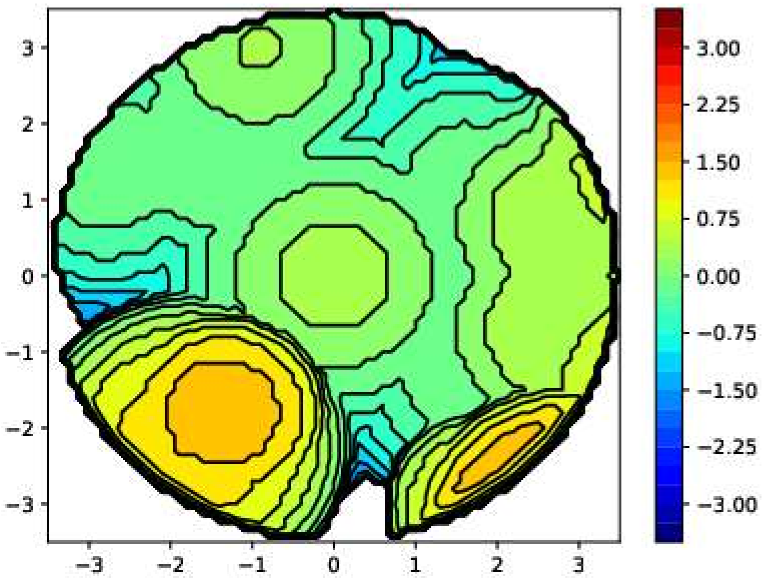

Figure S49. Topographic steric maps of **5**.

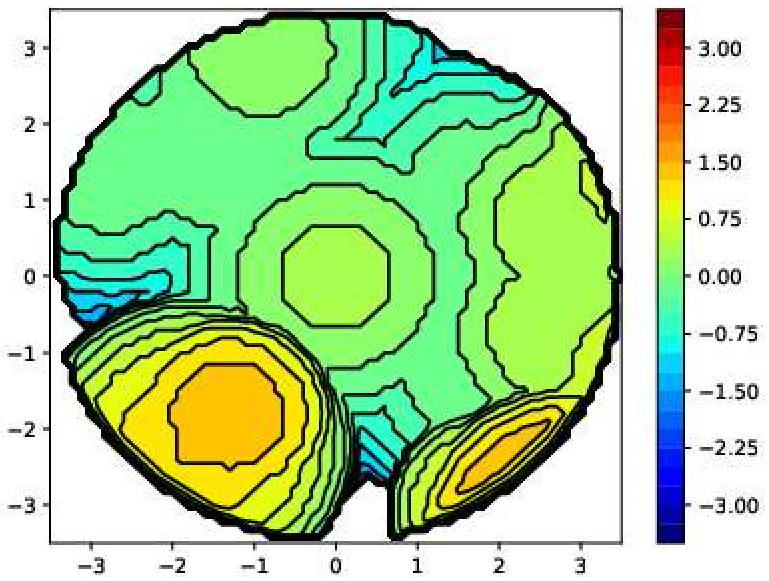

Figure S50. Topographic steric maps of **6**.

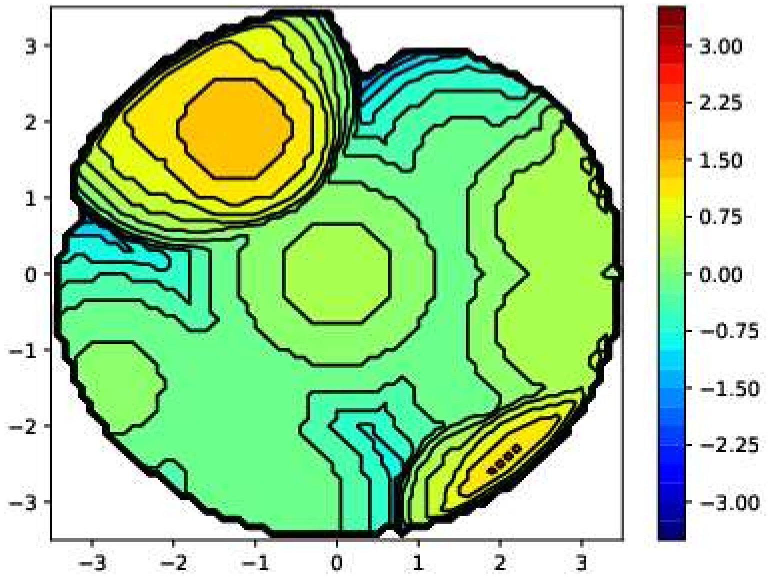

Figure S51. Topographic steric maps of **7**.

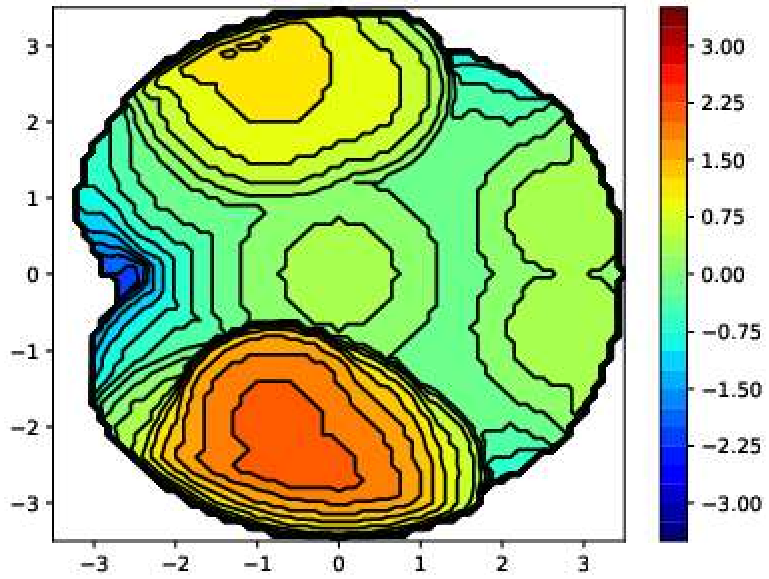

Figure S52. Topographic steric maps of **8**.

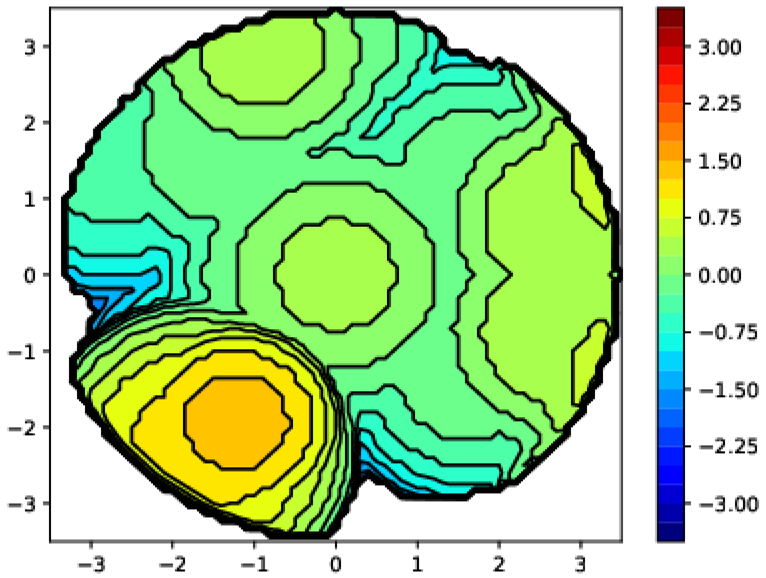

Figure S53. Topographic steric maps of **9**.

4.6 Visualized Electrostatic Potential Maps.

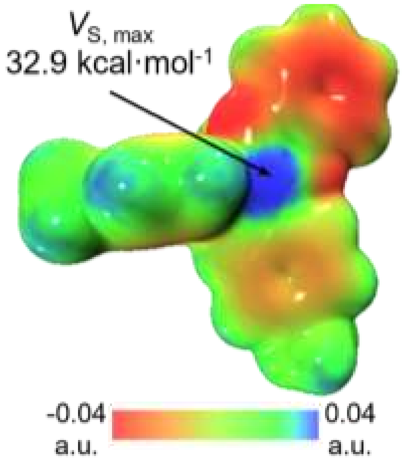

Figure S54. Electrostatic potential maps of **2**.

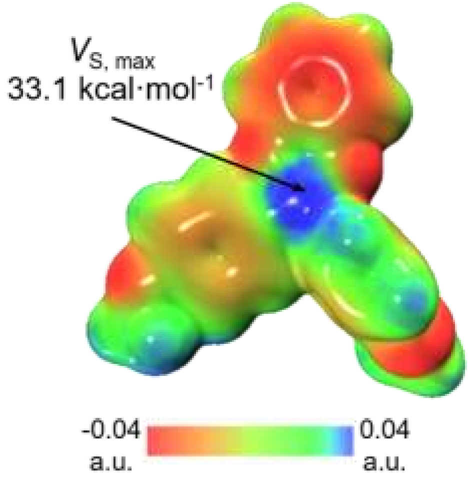

Figure S55. Electrostatic potential maps of **3**.

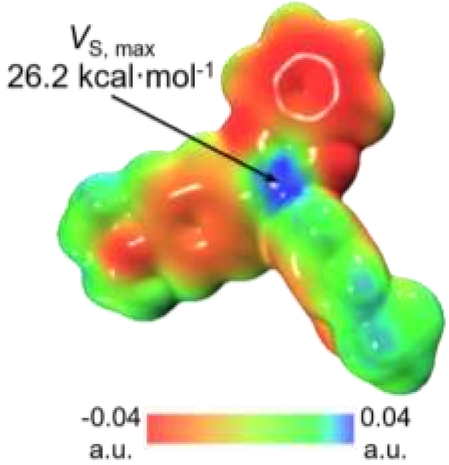

Figure S56. Electrostatic potential maps of **4**.

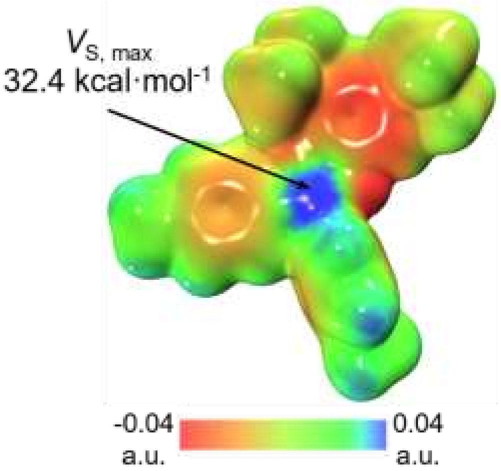

Figure S57. Electrostatic potential maps of **5**.

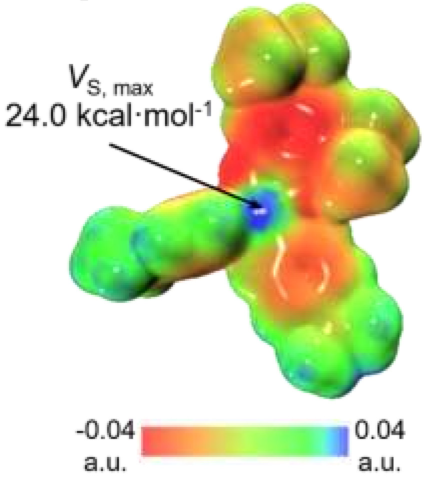

Figure S58. Electrostatic potential maps of **7**.

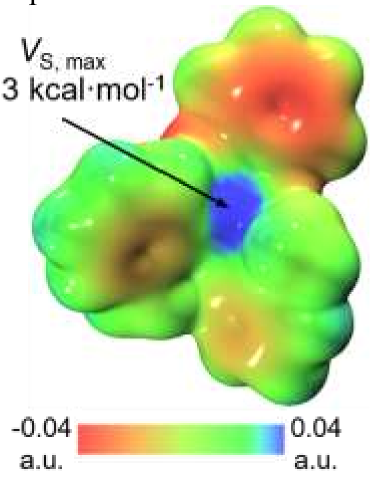

Figure S59. Electrostatic potential maps of **8**.

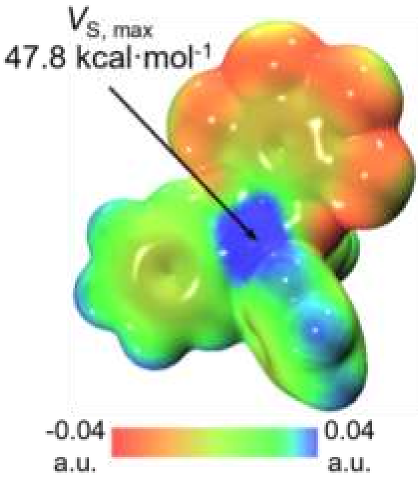

Figure S60. Electrostatic potential maps of **9**.

5 References

1 Beckmann, J. L.; Krieft, J.; Vishnevskiy, Y. V.; Neumann, B.; Stammler, H.-G.; Mitzel, N. W. A Bidentate Antimony Pnictogen Bonding Host System. *Angew. Chem. Int. Ed.* **2023**, *62*, e202310439.

2 Simpson, Q.; Sinclair, M. J. G.; Lupton, D. W.; Chaplin, A. B.; Hooper, J. F. Oxidative Cross-Coupling of Boron and Antimony Nucleophiles via Palladium(I). *Org. Lett.* **2018**, *20*, 5537-5540.

3 Egorova, I. V.; Zhidkov, V. V.; Grinishak, I. P.; Rodionova, N. A.; Bagryanskaya, I. Y.; Pervukhina, N. V. Synthesis and Structure of Antimony Complex Compounds [(4-*N,N*-$Me_2C_6H_4)_3MeSb$]I and [(4-N,N-$Me_2C_6H_4)_3MeSb]_2[Hg_2I_6]\cdot$2DMSO. *Russ. J. Gen. Chem.* **2021**, *91*, 1361-1367.

4 Murphy, B. L.; Gabbaï, F. P. Tunable Pnictogen Bonding at the Service of Hydroxide Transport across Phospholipid Bilayers. *J. Am. Chem. Soc.* **2024**, *146*, 7146-7151.

5 Sheldrick, G. Crystal structure refinement with SHELXL. *Acta Crystallogr. Sect. C.* **2015**, *71*, 3-8.

6 Dolomanov, O. V.; Bourhis, L. J.; Gildea, R. J.; Howard, J. A. K.; Puschmann, H. OLEX2: a complete structure solution, refinement and analysis program. *J. Appl. Crystallogr.* **2009**, *42*, 339-341.

7 Smith, B. The infrared spectra of polymers V: epoxies. *Spectroscopy* **2022**, *37*, 17-19.

8 Nandiyanto, A. B. D.; Ragadhita, R.; Fiandini, M. Interpretation of Fourier Transform Infrared Spectra (FTIR): A Practical Approach in the Polymer/Plastic Thermal Decomposition. *Indonesian Journal of Science and Technology* **2022**, *8*, 113-126.

9 Baek, S.-J.; Park, A.; Ahn, Y.-J.; Choo, J. Baseline correction using asymmetrically reweighted penalized least squares smoothing. *Analyst* **2015**, *140*, 250-257.

10 Malinowski, E. R. Window factor analysis: Theoretical derivation and application to flow injection analysis data. *J. Chemom.* **1992**, *6*, 29-40.

11 Shao, X.; Shao, L.; Li, M.; Lin, X. An improvement of window factor analysis for resolution of noisy HPLC-DAD data. *Sci. China, Ser. B: Chem.* **2002**, *45*, 289-298.

12 Li, P.; Cai, W.; Shao, X. Generalized window factor analysis for selective analysis of the target component in real samples with complex matrices. *J. Chromatogr. A* **2015**, *1407*, 203-207.

13 Rao, C. B.; Rao, D. C.; Venkateswara, M.; Venkateswarlu, Y. Protective opening of epoxide using pivaloyl halides under catalyst-free conditions. *Green Chem.* **2011**, *13*, 2704-2707.

14 Zhu, H.; Yalcin, T.; Li, L. Analysis of the Accuracy of Determining Average Molecular Weights of Narrow Polydispersity Polymers by Matrix-Assisted Laser Desorption Ionization Time-of-Flight Mass Spectrometry. *J. Am. Soc. Mass Spectrom.* **1998**, *9*, 275-281.

15 *Gaussian 16 Rev. C.01*; Wallingford, CT, 2016. (accessed.

16 Lee, C.; Yang, W.; Parr, R. G. Development of the Colle-Salvetti correlation-energy formula into a functional of the electron density. *Phys. Rev. B* **1988**, *37*, 785-789.

17 Becke, A. D. Density-functional thermochemistry. III. The role of exact exchange. *J. Chem. Phys.* **1993**, *98*, 5648-5652.

18 Peterson, K. A.; Figgen, D.; Goll, E.; Stoll, H.; Dolg, M. Systematically convergent basis sets with relativistic pseudopotentials. II. Small-core pseudopotentials and correlation consistent basis sets for the post-d group 16–18 elements. *J. Chem. Phys.* **2003**, *119*, 11113-11123.

19 Peterson, K. A.; Shepler, B. C.; Figgen, D.; Stoll, H. On the Spectroscopic and Thermochemical Properties of ClO, BrO, IO, and Their Anions. *J. Phys. Chem. A* **2006**, *110*, 13877-13883.

20 Petersson, G. A.; Bennett, A.; Tensfeldt, T. G.; Al-Laham, M. A.; Shirley, W. A.; Mantzaris, J. A complete basis set model chemistry. I. The total energies of closed-shell atoms and hydrides of the first-row elements. *J. Chem. Phys.* **1988**, *89*, 2193-2218.

21 Petersson, G. A.; Al-Laham, M. A. A complete basis set model chemistry. II. Open-shell systems and the total energies of the first-row atoms. *J. Chem. Phys.* **1991**, *94*, 6081-6090.

22 Ditchfield, R.; Hehre, W. J.; Pople, J. A. Self-Consistent Molecular-Orbital Methods. IX. An Extended Gaussian-Type Basis for Molecular-Orbital Studies of Organic Molecules. *J. Chem. Phys.* **1971**, *54*, 724-728.

23 Hehre, W. J.; Ditchfield, R.; Pople, J. A. Self—Consistent Molecular Orbital Methods. XII. Further Extensions of Gaussian—Type Basis Sets for Use in Molecular Orbital Studies of Organic Molecules. *J. Chem. Phys.* **1972**, *56*, 2257-2261.
24 Zapf, L.; Riethmann, M.; Föhrenbacher, S. A.; Finze, M.; Radius, U. An easy-to-perform evaluation of steric properties of Lewis acids. *Chem. Sci.* **2023**, *14*, 2275-2288.
25 Falivene, L.; Cao, Z.; Petta, A.; Serra, L.; Poater, A.; Oliva, R.; Scarano, V.; Cavallo, L. Towards the online computer-aided design of catalytic pockets. *Nat. Chem.* **2019**, *11*, 872-879.
26 Lu, T.; Chen, F. Multiwfn: A multifunctional wavefunction analyzer. *J. Comput. Chem.* **2012**, *33*, 580-592.
27 Humphrey, W.; Dalke, A.; Schulten, K. VMD: Visual molecular dynamics. *J. Mol. Graphics* **1996**, *14*, 33-38.
